# EXOSC9 depletion attenuates P-body formation, stress resistance, and tumorigenicity of cancer cells

**DOI:** 10.1038/s41598-020-66455-2

**Published:** 2020-06-09

**Authors:** Seiko Yoshino, Yusuke Matsui, Yuya Fukui, Masahide Seki, Kiyoshi Yamaguchi, Akane Kanamori, Yurika Saitoh, Teppei Shimamura, Yutaka Suzuki, Yoichi Furukawa, Shuichi Kaneko, Motoharu Seiki, Yoshinori Murakami, Jun-ichiro Inoue, Takeharu Sakamoto

**Affiliations:** 1 Division of Molecular Pathology, the Institute of Medical Science, The University of Tokyo, Shirokanedai, Minato-ku, Tokyo Japan; 2 Biomedical and Health Informatics Unit, Department of Integrated Health Science, Nagoya University Graduate School of Medicine, Daiko-Minami, Higashi-ku, Nagoya, Aichi Japan; 3 Division of Cellular and Molecular Biology, the Institute of Medical Science, The University of Tokyo, Shirokanedai, Minato-ku, Tokyo Japan; 4 Department of Computational Biology and Medical Sciences, Graduate School of Frontier Sciences, the University of Tokyo, Chiba, Japan; 5 Division of Clinical Genome Research, the Institute of Medical Science, The University of Tokyo, Shirokanedai, Minato-ku, Tokyo Japan; 6 Center for Medical Education, Teikyo University of Science, Senjusakuragi, Adachi-ku, Tokyo Japan; 7 Division of Systems Biology, Nagoya University Graduate School of Medicine, Tsurumai-cho, Nagoya, Japan; 8 Department of System Biology, Institute of Medical, Pharmaceutical and Health Sciences, Kanazawa University, Takaramachi, Kanazawa, Ishikawa Japan; 9 Division of Cancer Cell Research, the Institute of Medical Science, The University of Tokyo, Shirokanedai, Minato-ku, Tokyo Japan

**Keywords:** Cancer, Cancer therapeutic resistance

## Abstract

Cancer cells adapt to various stress conditions by optimizing gene expression profiles via transcriptional and translational regulation. However, whether and how EXOSC9, a component of the RNA exosome complex, regulates adaptation to stress conditions and tumorigenicity in cancer cells remain unclear. Here, we examined the effects of EXOSC9 depletion on cancer cell growth under various stress conditions. EXOSC9 depletion attenuated growth and survival under various stress conditions in cancer cells. Interestingly, this also decreased the number of P-bodies, which are messenger ribonucleoprotein particles (mRNPs) required for stress adaptation. Meanwhile, EXOSC2/EXOSC4 depletion also attenuated P-body formation and stress resistance with decreased EXOSC9 protein. EXOSC9-mediated stress resistance and P-body formation were found to depend on the intact RNA-binding motif of this protein. Further, RNA-seq analyses identified 343 EXOSC9-target genes, among which, APOBEC3G contributed to defects in stress resistance and P-body formation in MDA-MB-231 cells. Finally, EXOSC9 also promoted xenografted tumor growth of MDA-MB-231 cells in an intact RNA-binding motif-dependent manner. Database analyses further showed that higher EXOSC9 activity, estimated based on the expression of 343 target genes, was correlated with poorer prognosis in some cancer patients. Thus, drugs targeting activity of the RNA exosome complex or EXOSC9 might be useful for cancer treatment.

## Introduction

In tumor tissues, cancer cells adapt to various stress conditions such as hypoxia, nutrient starvation, oxidative stress, and chemotherapy by optimizing gene expression profiles via transcriptional and translational regulation. For example, cancer cells control the translation of some mRNAs by forming messenger ribonucleoprotein particles (mRNPs) such as processing-bodies (P-bodies) and stress granules (SGs) in the cytosol^1–3^. In addition, mRNA levels are well-balanced through transcription and degradation in cancer cells, especially under conditions of stress. Stress-type specific transcriptional factors such as HIF-1, NRF2, ATF4, and HSF1 are activated and promote their target gene expression under such conditions in tumor tissues^4–8^. Meanwhile, mRNA degradation is primed by particular motifs in the mRNA itself, such as AU-rich elements (AREs) and their binding proteins, following interaction with the 5′-3′ RNA degradation machinery including the decapping complex and the 5′-3′ exoribonuclease XRN1 and the 3′-5′ RNA degradation machinery, namely the RNA exosome complex^9–11^.

The RNA exosome is an evolutionally-conserved ribonuclease complex that consists of three cap proteins and six core proteins (EXOSC1–3 and EXOSC4–9 respectively in eukaryotes)^12,13^. Further, the RNA exosome complex in prokaryotes has RNase activity, whereas that in eukaryotes does not have RNase activity but rather functions as a pathway for recruited RNAs to be effectively degraded/processed by RNA exosome-binding RNases such as DIS3, DIS3L, and EXOSC10^14–16^. The RNA exosome complex functions in the turnover and quality control of various RNAs including mRNAs with AREs in the nucleus and cytosol by interacting with other cofactors^12,17^. Nine RNA exosome components are thought to function in an integrated manner. However, interestingly, mutations in EXOSC3 and EXOSC8 cause similar pontocerebellar hypoplasia, whereas those in EXOSC2 cause defects in various tissues, such as retinitis pigmentosa, progressive sensorineural hearing loss, hypothyroidism, premature aging, and mild intellectual disability in humans, indicating the possibility that each RNA exosome component has different effect in tissue- and cell-type dependent manners^13,18–20^. However, although the RNA exosome complex plays an essential role in RNA metabolism, its contribution to other diseases including cancer is poorly understood.

EXOSC9 is one of the core proteins of the RNA exosome complex and was originally identified as a polymyositis/scleroderma autoantigen in humans^21^. There are still few reports on the physiological and pathological functions of this protein; however, it is known that it is necessary for the normal differentiation of epidermal tissues^22^ and that patients with mutations in the *EXOSC9* gene show cerebellar hypoplasia and abnormalities in motor neurons, which are also caused by similar mutations in other RNA exosome component genes^23^. Previously, we identified *EXOSC9* as an essential gene for lung and cancer cell growth during hypoxia based on genome-wide shRNA library screening^24^. However, whether and how EXOSC9 regulates adaptation to other stress conditions and tumorigenicity in cancer cells remain unclear. To address this, here, we examined cell growth under different stress conditions such as nutrient starvation, genotoxic stress, endoplasmic reticulum (ER) stress, and oxidative stress, as well as tumorigenicity, using EXOSC9-depleted cancer cells.

## Results

### EXOSC9 is necessary for stress resistance

**Figure 1:**
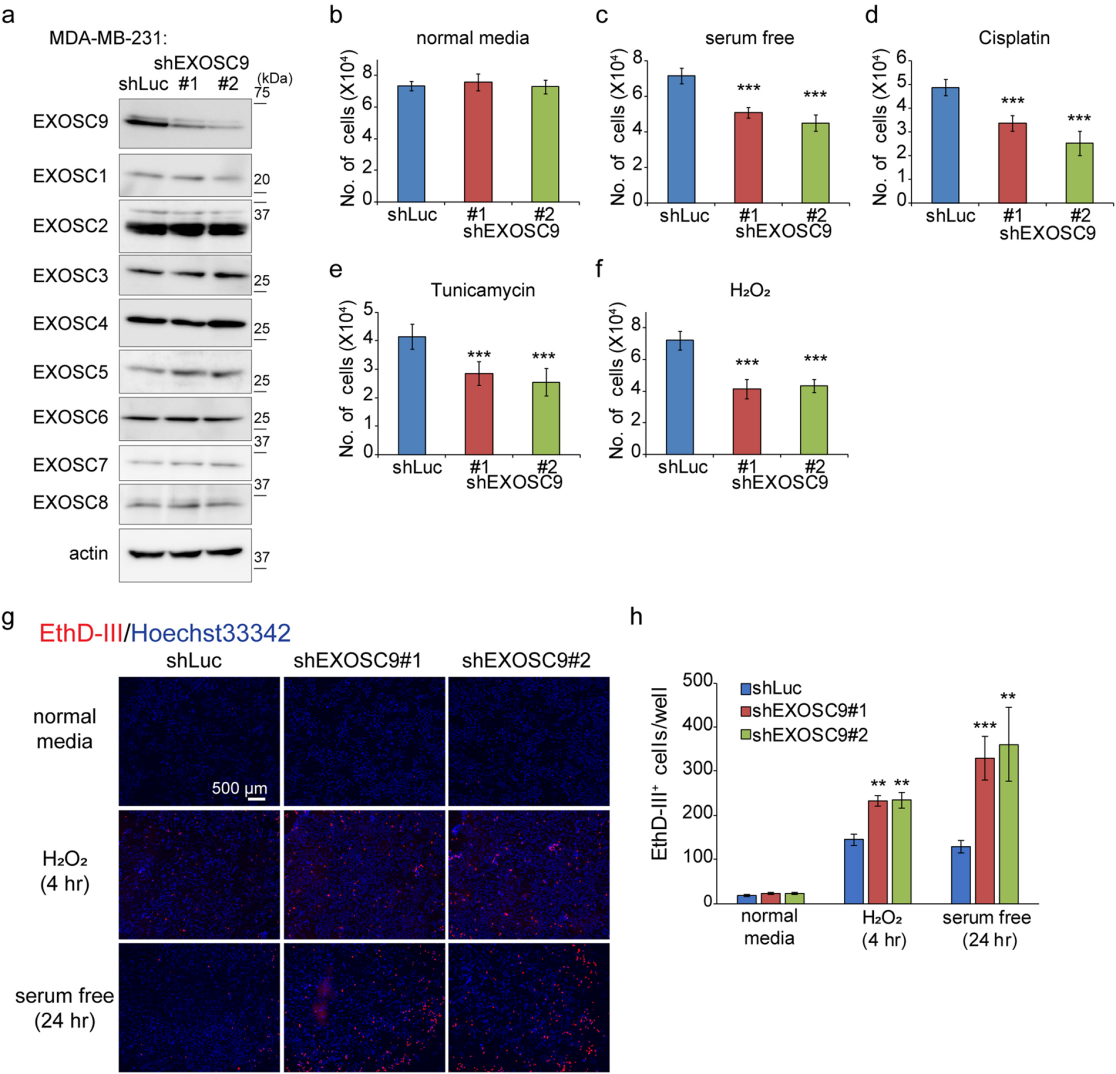
EXOSC9 is necessary for stress resistance. **(a)** Expression of EXOSC9 and other RNA exosome components in control (shLuc) and EXOSC9-depleted (shEXOSC9#1, #2) MDA-MB-231 cells. **(b–f)** Cell number of control and EXOSC9-depleted MDA-MB-231 cells cultured in normal media (**b**), serum free media (**c**), or normal media in the presence of cisplatin (40 μM) (**d**), tunicamycin (10 μg/mL) (**e**), or H_2_O_2_ (100 μM) (**f**) for 24 h. **(g**,**h)** Dying or dead cells were stained with EthD-III dye (red) and nuclei were stained with Hoechst33342 dye. (**g)** Representative photos of EthD-III- and Hoechst33342-stained MDA-MB-231 cells cultured under indicated conditions. (**h)** EthD-III-positive cells were counted. In (**b**–**f**,**h)**, n = 9 from three independent experiments. Data represent mean ± SD. **p < 0.01, ***p < 0.001 by Student’s t-test.

To evaluate the function of EXOSC9 in stress resistance in cancer cells, we first established stable EXOSC9-depleted breast cancer MDA-MB-231 cells using shRNA-expressing lentiviral vectors. EXOSC9 depletion in MDA-MB-231 cells did not affect the expression of other RNA exosome components (EXOSC1-8), exosome-associated 5ʹ-3ʹ exoribonucleases (EXOSC10, DIS3, DIS3L), or exosome cofactors (HBS1L, MPHOSPH6, C1D, RBM7; Fig. 1a and Supplementary Fig. S1a), as previously reported^25^.

RNA exosome depletion has also been reported to result in the accumulation of promoter upstream transcripts (PROMPTs) that are produced ~0.5 to 2.5 kilobases upstream of the active transcription start sites in human cells^26^. Thus, we next examined the levels of PROMPTs in control, EXOSC9-, EXOSC2-, and EXOSC4-depleted MDA-MB-231 cells, and found that EXOSC9 depletion significantly increased the level of PROMPTs; however, this increase was moderate compared to that observed following EXOSC2 or EXOSC4 depletion (Supplementary Fig. S1b). Control and EXOSC9-depleted MDA-MB-231 cells were then subjected to various stress conditions. While downregulating this marker did not affect cell proliferation when cells were cultured in normal culture media (Fig. 1b), EXOSC9-depleted MDA-MB-231 cells showed decreased cell numbers upon exposure to serum starvation (Fig. 1c), cisplatin-induced genotoxic stress (Fig. 1d), tunicamycin-induced ER stress (Fig. 1e), and oxidative stress mediated by H_2_O_2_ (Fig. 1f), as compared to control cell numbers. EXOSC9 depletion also affected the number of breast cancer MCF-7 and cervical cancer HeLa cells upon exposure to conditions of stress (Supplementary Fig. S2). The number of EthD-III positive dying or dead cells^27^ also increased in EXOSC9-depleted MDA-MB-231 cells compared to that in control cells after serum starvation or H_2_O_2_ treatment (Fig. 1g,h). Taken together, EXOSC9 is indispensable for the survival of cancer cells under various conditions of stress.

### EXOSC9 is necessary for P-body formation

**Figure 2:**
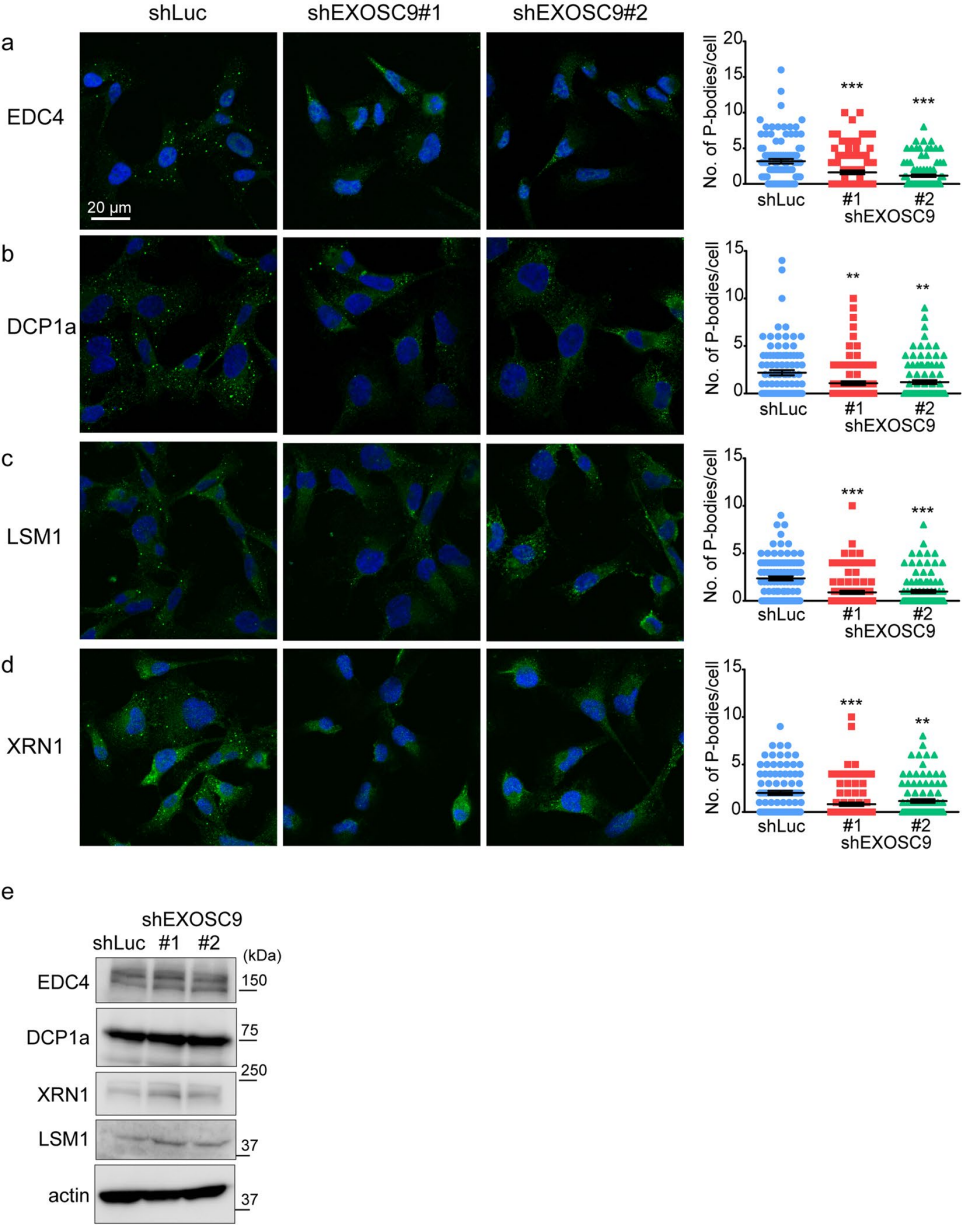
EXOSC9 is necessary for P-body formation. **(a–d)** Immunostaining of P-body markers EDC4 (**a**), DCP1a (**b**), LSM1 (**c**), and XRN1 (**d**) in control (shLuc) and EXOSC9-depleted (shEXOSC9#1, #2) MDA-MB-231 cells. (Left) representative photos. (Right) the number of indicated P-body marker-positive granules in a cell was counted. **(e)** Expression of p-body marker proteins in control and EXOSC9-depleted cells. In **(a**–**d**), n = 100 per group. Data represent mean ± SEM. **p < 0.01, ***p < 0.001 by Mann-Whitney U-test.

Because EXOSC9 depletion affected resistances to various stressors, we hypothesized that it controls cellular machineries involved in the general stress response. P-bodies are known as mRNPs that are required for the stress response, wherein translation from sequestered mRNAs is paused and the decay of these mRNAs is controlled in response to cellular conditions^2,28,29^. Indeed, P-body depletion by knockdown of a P-body component EDC4 attenuated resistances to various stressors in MDA-MB-231 cells (Supplementary Fig. S3). Although the RNA exosome complex does not exist in P-bodies^9,29^, intriguingly, EXOSC9 depletion decreased the number of foci comprising P-body markers such as EDC4 (Fig. 2a), DCP1a (Fig. 2b), LSM1 (Fig. 2c), and XRN1 (Fig. 2d) in MDA-MB-231 cells under normal culture conditions without decreasing the levels of these proteins (Fig. 2e). Even in MCF-7 and HeLa cells, EXOSC9 depletion significantly decreased P-bodies (Supplementary Figs. S4, S5). These results indicate that EXOSC9 is necessary for P-body formation in cancer cells under steady-state conditions.

**Figure 3:**
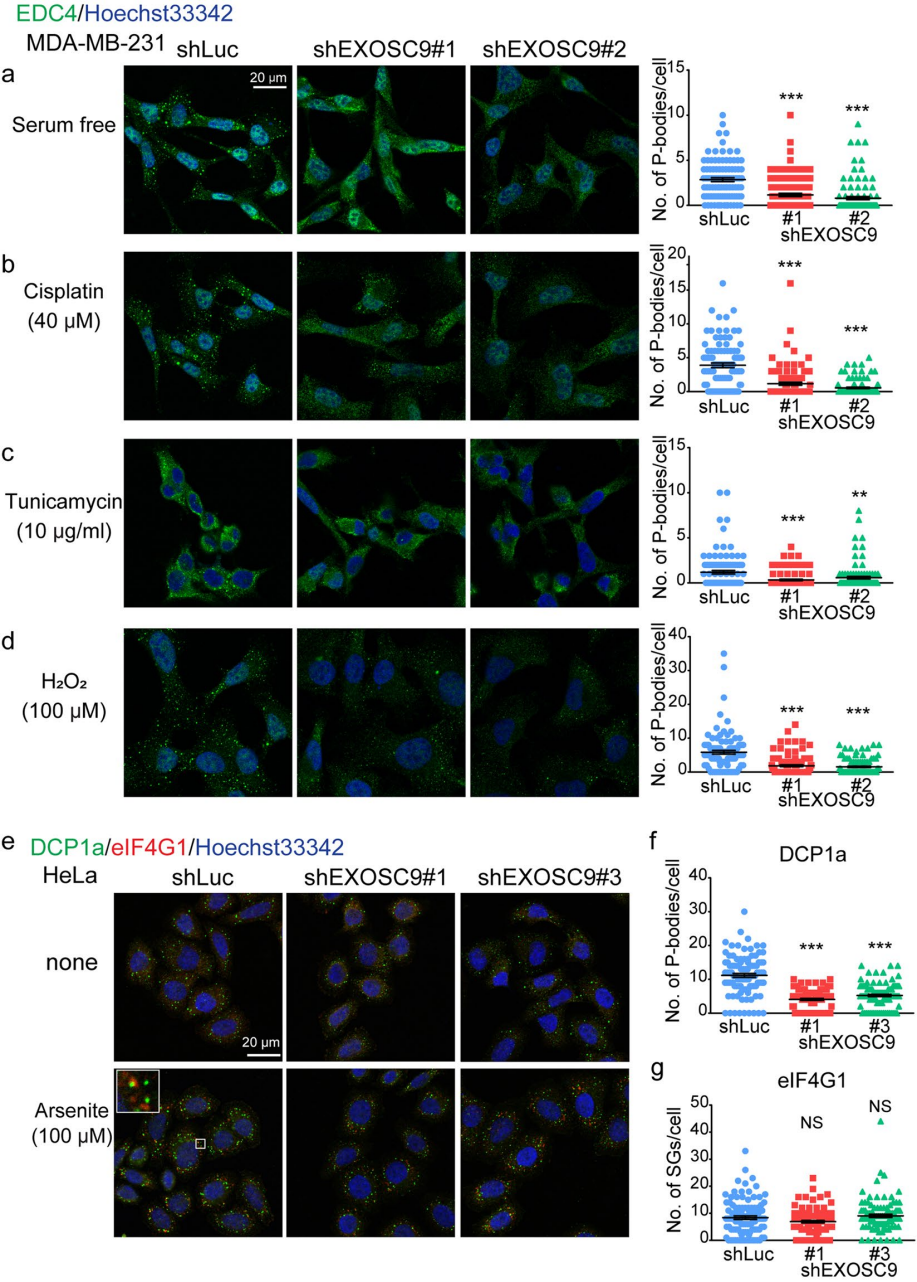
EXOSC9 depletion attenuates P-body formation under stress conditions. **(a–d)** Immunostaining of EDC4 in control (shLuc) and EXOSC9-depleted (shEXOSC9#1, #2) MDA-MB-231 cells cultured in serum free media (**a**) or normal media with cisplatin (40 μM) (**b**), tunicamycin (10 μg/mL) (**c**), or H_2_O_2_ (10 μM) (**d**) for 24 h. (Left) representative photos. (Right) the number of indicated P-body marker-positive granules in a cell was counted. **(e–g)** Immunostaining of the P-body marker DCP1a and the stress granule (SG) marker eIF4G1 in control (shLuc) and EXOSC9-depleted (shEXOSC9#1, #3) HeLa cells treated with or without arsenite (100 μM) for 30 min. (**e**) Representative photos of immunostaining. Inset shows P-bodies and SGs with higher magnification. (**f**,**g)** The number of DCP1a- (**f**) or eIF4G1- (**g**) positive granules in a cell was counted after arsenite treatment. In **(a–d**,**f**,**g)**, n = 100 per group. Data represent mean ± SEM. **p < 0.01, **p < 0.001 by Mann-Whitney U-test. NS, not significant.

The number of P-bodies increases or decreases in response to the type and duration of stress^29^. Thus, we next examined whether EXOSC9 depletion also affects P-body formation under various stress conditions in MDA-MB-231 cells. Even when cells were subjected to serum starvation (Fig. 3a), cisplatin (Fig. 3b), tunicamycin (Fig. 3c), and H_2_O_2_ (Fig. 3d), EXOSC9-depleted MDA-MB-231 cells showed fewer EDC4-positive P-bodies compared to numbers in control cells. Whereas P-bodies are constitutively present, SGs, which are other mRNPs closely related to P-bodies, are formed in response to particular stress in some types of cells^1,2^. We could not detect SG formation in MDA-MB-231 and MCF-7 cells in response to serum starvation or cisplatin, tunicamycin, H_2_O_2_, and arsenite treatment; however, arsenite treatment induced eIF4G1-positive SG formation adjacent to P-bodies in HeLa cells (Fig. 3e, inset). EXOSC9 depletion did not affect the number of eIF4G1-positive SGs but still decreased DCP1a-positive P-bodies in HeLa cells treated with arsenite (Fig. 3e–g). Thus, EXOSC9 controls P-body formation but not SG formation even under conditions of stress.

### RNA exosome complex components are necessary for stress resistance and P-body formation

**Figure 4:**
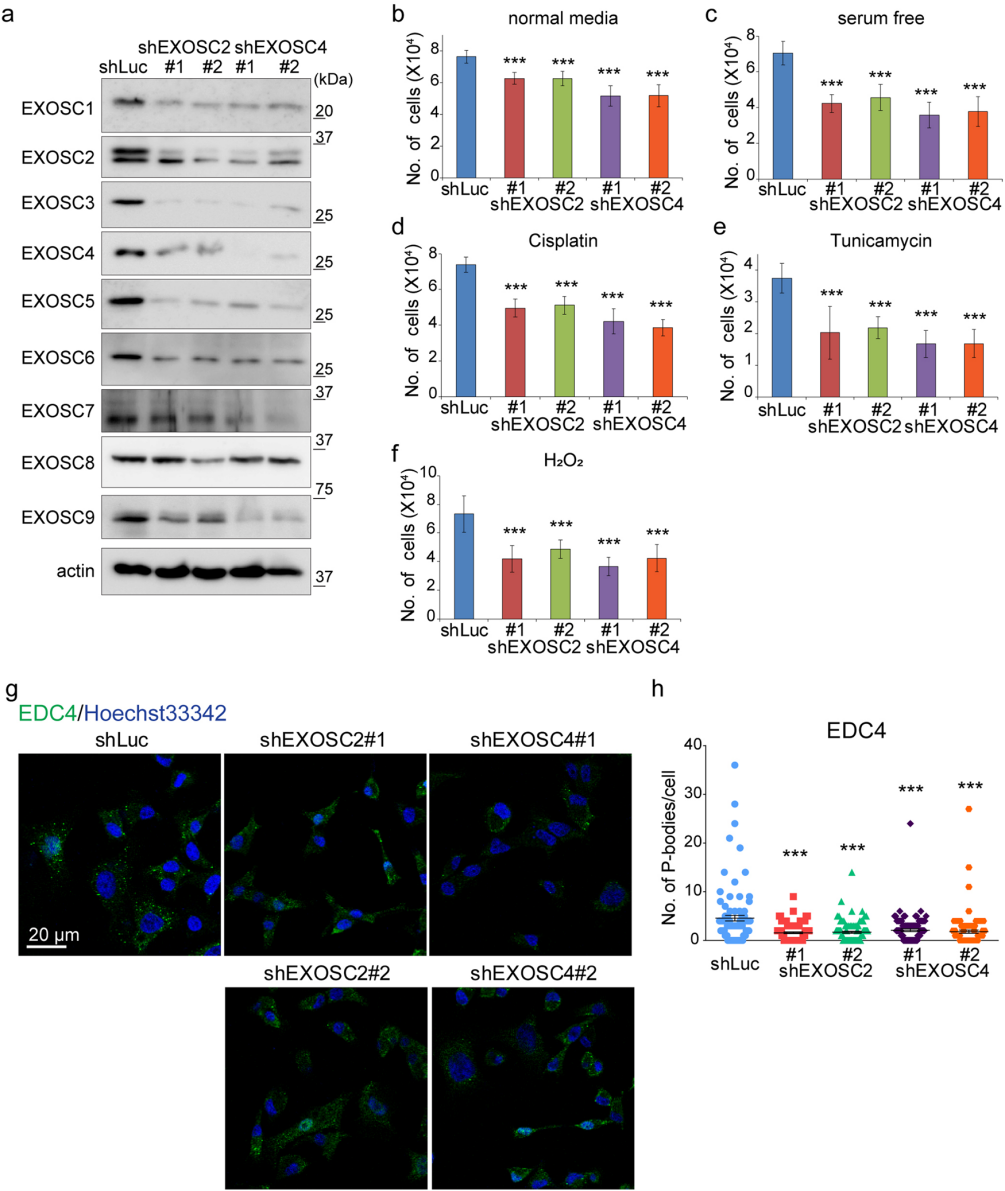
RNA exosome components are necessary for stress resistance and P-body formation. **(a)** Expression of the RNA exosome components in control (shLuc), EXOSC2-depleted (shEXOSC2#1, #2), and EXOSC4-depleted (shEXOSC4#1, #2) MDA-MB-231 cells. **(b–f)** Cell numbers for control, EXOSC2-depleted, and EXOSC4-depleted MDA-MB-231 cells cultured in normal media (**b**), serum free media (**c**), or normal media in the presence of cisplatin (40 μM) (**d**), tunicamycin (10 μg/mL) (**e**), or H_2_O_2_ (100 μM) (**f**) for 24 h. n = 9 from three independent experiments. Data represent mean ± SD. ***p < 0.001 by Student t-test. **(g,h)** Immunostaining for the P-body marker EDC4 in control, EXOSC2-depleted, and EXOSC4-depleted MDA-MB-231 cells. **(g**) Representative photos. **(h)** The numbers of indicated P-body marker-positive granules in a cell were counted. n = 100 per group. Data represent mean ± SEM. ***p < 0.001 by Mann-Whitney U-test.

The RNA exosome core complex consists of nine subunits^12,13^. To examine whether depletion of other RNA exosome components also causes defects in stress resistance and P-body formation, we selected EXOSC2, a cap protein, and EXOSC4, a ring protein, and knocked down the genes encoding these proteins in MDA-MB-231 cells. Unlike EXOSC9 depletion, EXOSC2 or EXOSC4 knockdown resulted in the downregulation of most RNA exosome components at the protein level, including EXOSC9 (Fig. 4a). These results corresponded to previous reports indicating that the depletion or mutation of one RNA exosome component, except EXOSC9, causes instability of other RNA exosome protein components^23,25^. Depletion of EXOSC2 or EXOSC4 decreased the number of MDA-MB-231 cells under not only stress conditions but also normal culture conditions (Fig. 4b–f). Depletion of EXOSC2 or EXOSC4 also decreased EDC4-positive P-body formation, similar to that observed with EXOSC9 depletion (Fig. 4g,h). Taken together, defects in stress resistance and P-body formation are common with the downregulation of RNA exosome components, namely EXOSC2, EXOSC4, and EXOSC9. Meanwhile, the depletion of EXOSC2 or EXOSC4 caused more severe effects, as compared to those with EXOSC9 knockdown, such as the instability of RNA exosome components and growth retardation in normal culture media in MDA-MB-231 cells, indicating that this protein has unique features among RNA exosome components.

### The intact RNA-binding motif is necessary for EXOSC9-mediated stress resistance and P-body formation

**Figure 5:**
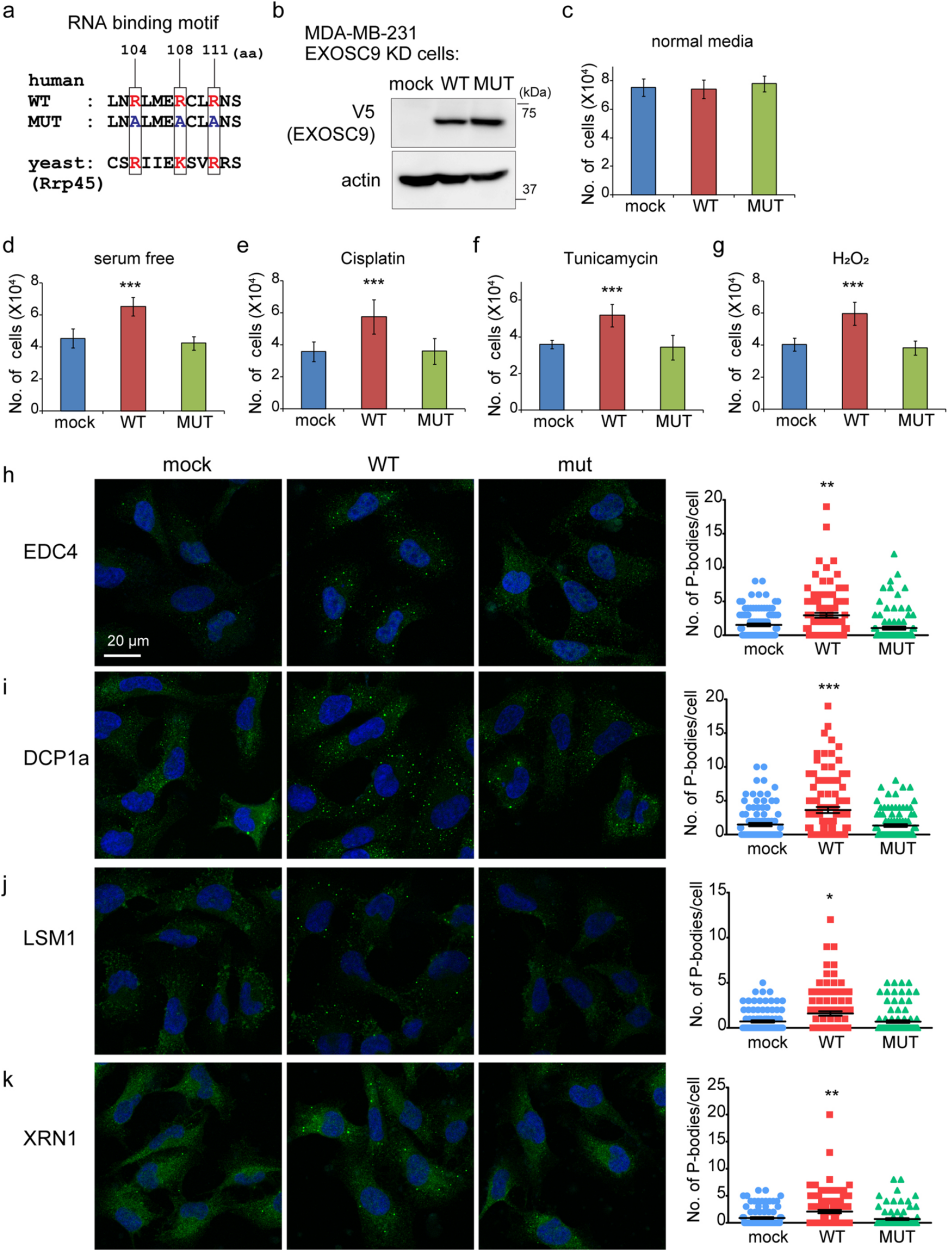
Intact RNA-binding motif is necessary for EXOSC9-mediated stress resistance and P-body formation. **(a)** Amino acid sequences of the RNA binding motif from human EXOSC9 and its yeast homologue. Positively charged residues in red are essential for RNA binding. **(b)** Expression of mock and V5-tagged wild-type (WT) and mutant (MUT) EXOSC9 in EXOSC9-knockdown (KD) MDA-MB-231 cells. **(c–g)** Cell number of EXOSC9 KD MDA-MB-231 cells expressing mock and WT and MUT EXOSC9 cultured under indicated conditions for 24 h. n = 9 from three independent experiments. Data represent mean ± SD. ***p < 0.001 by Student’s t-test. **(h–k)** Immunostaining for indicated P-body markers in EXOSC9 KD MDA-MB-231 cells expressing mock and WT and MUT EXOSC9. (Left) representative photos. (Right) the number of indicated P-body marker-positive granules in a cell was counted. n = 100 per group. Data represent mean ± SEM. *p < 0.05, **p < 0.01, ***p < 0.001 by Mann-Whitney U-test.

EXOSC9 has an RNA-binding motif that is conserved among species^14^. Arginine residues in the RNA-binding motif are essential for RNA binding, therefore, mutating these residues in EXOSC9 abolishes the RNase activity of the RNA exosome complex *in vitro*^14^. Thus, to clarify whether the RNA-binding motif is involved in EXOSC9-mediated stress resistance and P-body formation, mock, V5-tagged wild-type (WT) EXOSC9, or a mutant with R104A, R108A, and R111A substitutions in the RNA-binding motif (MUT) were re-expressed in EXOSC9 knockdown (KD) MDA-MB-231 cells (Fig. 5a,b). WT EXOSC9 decreased expression of PROMPTs, while MUT EXOSC9 did not (Supplementary Fig. S6a), indicating that the RNA-binding motif of EXOSC9 is necessary for PROMPT regulation. These cells were then subjected to stress conditions where WT EXOSC9 was found to restore stress resistance in EXOSC9 KD MDA-MB-231 cells, however, MUT EXOSC9 did not have the same effect (Fig. 5c–g). In parallel with these results, WT EXOSC9, and not MUT EXOSC9, also restored P-body formation in EXOSC9 KD MDA-MB-231 cells (Fig. 5h–k). Thus, an intact RNA-binding motif is necessary for EXOSC9-mediated stress resistance and P-body formation.

### EXOSC9 targets *APOBEC3G* mRNA

**Figure 6:**
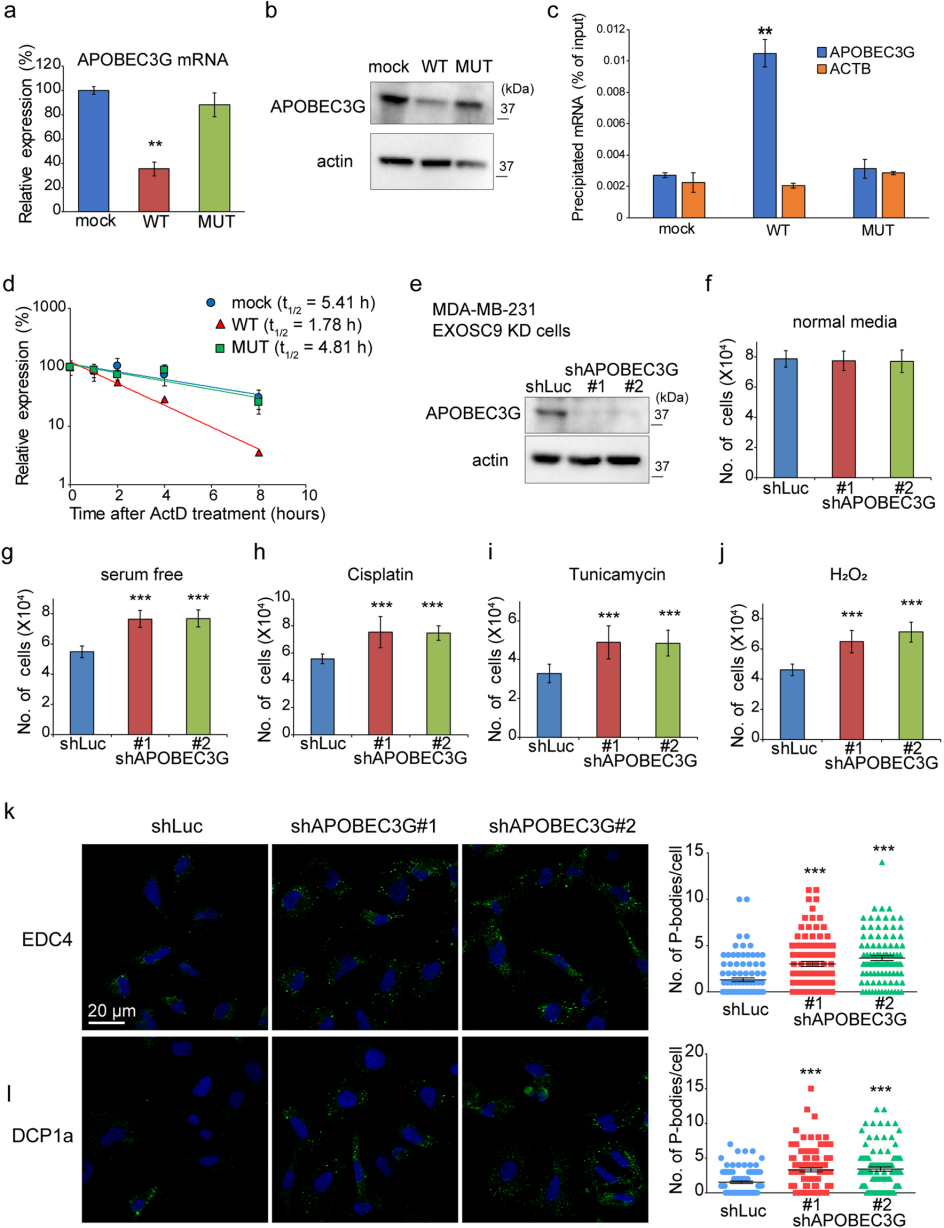
EXOSC9 targets *APOBEC3G* mRNA to control stress resistance and P-body formation. **(a,b)** Expression of *APOBEC3G* mRNA (**b**) and protein (**c**) in mock and WT and MUT EXOSC9 expressing EXOSC9 KD MDA-MB-231 cells. In **(b**), n = 3. Data represent mean ± SD. **p < 0.01 by Student’s t-test. **(c)** RNA immunoprecipitation assay using anti-V5 antibodies. V5-tagged WT EXOSC9 bound to APOBEC3G mRNA, while V5-tagged MUT EXOSC9 did not. No significant difference was observed in binding to ACTB mRNA among the mock, WT, and MUT EXOSC9 cells. n = 3. Data represent mean ± SD. **p < 0.01 as determined by Student’s *t*-tests. **(d)** *APOBEC3G* mRNA levels after actinomycin D (5 μg/mL) treatment were analyzed by qRT-PCR. **(e)** Expression of APOBEC3G protein in control (shLuc) and APOBEC3G-depleted (shAPOBEC3G#1, #2) EXOSC9 KD MDA-MB-231 cells. **(f–j)** APOBEC3G depletion restored cell proliferation under indicated stress conditions in EXOSC9 KD MDA-MB-231 cells. n = 9 from three independent experiments. Data represent mean ± SD. ***p < 0.001 by Student’s t-test. **(k**,**l)** Indicated P-body markers were stained in control and APOBEC3G-depleted EXOSC9 KD MDA-MB-231 cells (left). The number of indicated P-body marker foci in a cell was counted (right). n = 100. Data represent mean ± SEM. ***p < 0.001 by the Mann-Whitney U-test.

EXOSC9 and other RNA exosome components are involved in stress resistance and P-body formation in cancer cells. However, the RNA exosome complex does not exist in P-bodies^9,29^. Indeed, both WT and MUT EXOSC9 localized mainly to the nucleus and partially to the cytosol but did not form P-body-like foci in EXOSC9 KD MDA-MB-231 cells (Supplementary Fig. S6b). Thus, we hypothesized that EXOSC9 might indirectly support P-body formation by controlling the mRNA levels of P-body related genes. To address this, the mRNA expression profiles among mock, WT EXOSC9-, and MUT EXOSC9-expressing EXSOSC9 KD MDA-MB-231 cells were compared by RNA-seq. We selected genes showed 1.5-fold or more increase/decrease in WT EXOSC9-expressing cells, but not in MUT EXOSC9-expressing cells, compared with the mock-expressing cells, and the difference in expression levels between mock and WT EXOSC9-expressing cells was statistically significant (adjusted p value <0.05) by the DESeq2 analysis^30^. Using these criteria, 196 overrepresented and 343 underrepresented genes specific to WT-EXOSC9-expressing cells were identified (Supplementary Tables S1, S2). Since the RNA exosome complex functions as machinery for mRNA decay, these underrepresented 343 genes were considered possible EXOSC9-target genes. We then confirmed, via RT-qPCR, that the mRNA levels of stress/cell death-related genes, including *DAPK1*, *PYCARD*, *TNFRSF1B*, and *TNFRSF21*, among the underrepresented genes were significantly downregulated in WT EXOSC9 expressing cells (Supplementary Fig. S6c). The relationship between each candidate gene and P-body formation was then examined resulting in the identification of APOBEC3G as a possible regulator of EXOSC9-mediated P-body formation. APOBEC3G is a single-strand DNA/RNA deaminase that is involved in host defense against HIV infection^31–34^. APOBEC3G localizes to P-bodies^35–37^ and its overexpression was found to decrease P-body formation in HeLa cells^38^. Thus, we first examined mRNA levels of *APOBEC3G* in EXOSC9 KD MDA-MB-231 cells expressing mock, WT, and MUT EXOSC9, and found that WT EXOSC9, but not MUT EXOSC9, decreased *APOBEC3G* mRNA levels in these cells (Fig. 6a). APOBEC3G protein was also inversely correlated with EXOSC9 expression (Fig. 6b and Supplementary Fig. S6d,e). Further, RNA immunoprecipitation assays with anti-V5 antibodies, showed that WT EXOSC9, and not MUT EXOSC9, bound to APOBEC3G mRNA (Fig. 6c). Next, the half-life of *APOBEC3G* mRNA was assessed in these cells by suppressing transcription using actinomycin D. WT EXOSC9-expressing cells showed a shorter *APOBEC3G* half-life (1.78 h) compared to that with mock (5.41 h)- and MUT EXOSC9 (4.81 h)-expressing cells (Fig. 6d). These results indicate that EXOSC9 promotes the decay of *APOBEC3G* mRNA.

### APOBEC3G depletion restores stress resistance and P-body formation in EXOSC9-depleted MDA-MB-231 cells

Subsequently, APOBEC3G was stably knocked down in EXOSC9 KD MDA-MB-231 cells to examine whether increased expression contributes to the observed defects in stress resistance and P-body formation upon EXOSC9 depletion (Fig. 6e). APOBEC3G knockdown restored stress resistance in EXOSC9 KD MDA-MB-231 cells without affecting proliferation in normal culture media (Fig. 6f–j). Similarly, APOBEC3G knockdown also restored P-body formation in EXOSC9 KD MDA-MB-231 cells under steady-state conditions (Fig. 6k,l and Supplementary Fig. S6f,g). Taken together, increased APOBEC3G was determined to be responsible for the defects in stress resistance and P-body formation observed with EXOSC9 depletion in MDA-MB-231 cells.

### EXOSC9 promotes tumor malignancy

**Figure 7:**
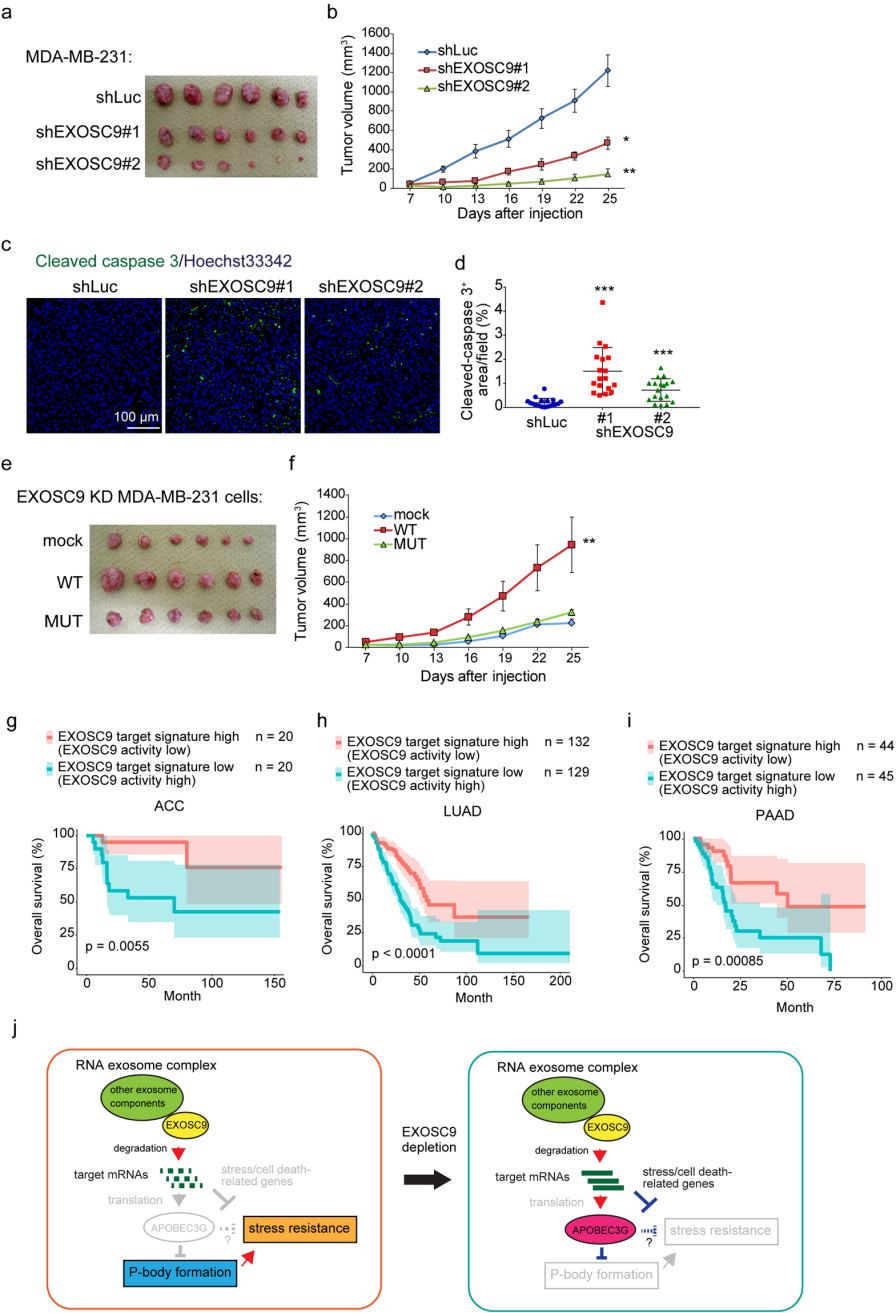
EXOSC9 promotes tumorigenicity. **(a,b)** Control (shLuc) and EXOSC9-depleted (shEXOSC9#1, #2) MDA-MB-231 cells were injected subcutaneously into nude mice. **(a**) Photos of the tumors at day 25. **(b)** Tumor growth was analyzed at the indicated day. n = 6 per group. **(c,d)** Immunostaining for cleaved caspase-3 in day-25 tumors from control and EXOSC9-depleted MDA-MB-231 cells. **c** Representative photos. **(d)** Cleaved caspase-3-positive areas were counted in tumor sections. n = 18 from six tumors per group. **(e,f)** EXOSC9-depleted MDA-MB-231 cells expressing mock or WT or MUT EXOSC9 were injected subcutaneously into nude mice. **e** Photos of tumors at day 25. **(f**) Tumor growth was analyzed at the indicated day. n = 6 per group. In (**b**,**d**,**f)**, data represent mean ± SEM. *p < 0.05, **p < 0.01, ***p < 0.001 by the Mann-Whitney U-test. **(g–i)**. A low EXOSC9-target signature was found to correlate with poor prognosis in adrenocortical carcinoma (ACC) (**g**), lung adenocarcinoma (LUAD) (**h**), and pancreatic adenocarcinoma (PAAD) (**i**) patients. The data were retrieved from combined datasets from the TCGA database and analyzed by the Log-rank test. **(j)** Graphical illustration of EXOSC9-mediated P-body formation and stress resistance in cancer cells.

Cancer cells need to overcome various stresses such as nutrient starvation, hypoxia, and oxidative stress to form tumors *in vivo*. Thus, the role of EXOSC9 in tumor formation was evaluated using xenograft assays. EXOSC9 depletion markedly attenuated MDA-MB-231 cell tumor growth in immunodeficient mice (Fig. 7a,b). Further, 25 days after inoculation, more apoptotic cells were detected in tumors formed by EXOSC9 KD MDA-MB-231 cells, compared to numbers in control tumors (Fig. 7c,d), which was concomitant with smaller tumor volumes. Moreover, the re-expression of WT EXOSC9, but not MUT EXOSC9, restored the tumor growth of EXOSC9-depleted MDA-MB-231 cells (Fig. 7e,f). Thus, EXOSC9 supports the tumorigenicity of MDA-MB-231 cells in an intact RNA-binding motif-dependent manner, as observed for vitro stress resistance and P-body formation.

EXOSC9 is a core component of the RNA exosome, but the expression of only this marker is insufficient to estimate RNA exosome complex activity in tumor tissues. Thus, we set 343 EXOSC9-target gene candidates including *APOBEC3G* in MDA-MB-231 cells as the “EXOSC9 target signature” and evaluated the correlation between this signature and cancer patient prognosis using the TCGA datasets. The low-EXOSC9 target signature, indicating high EXOSC9 activity, was significantly correlated with poor prognosis in adrenocortical carcinoma, lung adenocarcinoma, and pancreatic adenocarcinoma, among various cancers from the TCGA datasets (Fig. 7g–i). Thus, high EXOSC9 activity might result in a worse prognosis for these cancers.

## Discussion

In this study, we revealed that EXOSC9, a component of the 3′-5′ mRNA degradation machinery RNA exosome complex, promotes the formation of P-bodies, which is likely associated with the translational regulation of some mRNAs, stress resistance, and tumorigenicity in cancers (Fig. 7j). Indeed, EXOSC9 depletion induced excess *APOBEC3G* mRNA expression and knockdown of APOBEC3G restored P-body formation and stress resistance in EXOSC9-depleted MDA-MB-231 cells. However, these results cannot exclude the possibility that the degradation of other EXOSC9-target mRNAs or the processing of small RNAs in the nucleus by EXOSC9 also contribute to P-body formation and/or stress resistance in cancer cells. In fact, in addition to APOBEC3G, stress/cell death-related genes were found to be downregulated in EXOSC9-expressing MDA-MB-231 cells (Supplementary Fig. S6c), suggesting that EXOSC9 can control stress resistance in both P-body formation-dependent and independent manners.

A recent paper reported that human patients with mutations that decrease EXOSC9 expression show cerebellar hypoplasia similar to that observed with mutations in other RNA exosome components such as *EXOSC3* and *EXOSC8* genes^18,20,23^. We checked the data in this study and found that *APOBEC3G* mRNA levels were also increased in muscle cells from a patient with an *EXOSC9* mutation compared to those in tissue from a healthy donor^23^, supporting our findings in cancer cells. The RNA exosome complex is thought to promote mRNA degradation with AREs in the 3′UTR^15,39,40^, and *APOBEC3G* mRNA indeed contains such motifs in its 3′UTR^23^. However, all mRNAs with AREs were not upregulated by EXOSC9 depletion in MDA-MB-231 cells, and only 17 genes including those without AREs were commonly upregulated in muscle cells and fibroblasts from a patient with an EXOSC9 mutation and a healthy donor^23^. In parallel, lower expression of the EXOSC9-target signature was also found to correlate with poor prognosis in some cancers among various cancers from the TCGA datasets. This indicates that genes targeted by EXOSC9 and the RNA exosome complex are defined in a cell-type specific manner and by other factor(s) such as the expression patterns of RNA-binding proteins. *APOBEC3G* mRNA regulation by EXOSC9 might also depend on the type of cancer.

Increased levels of this protein were also found to attenuate P-body formation in EXOSC9-depleted MDA-MB-231 cells (Fig. 6k,l and Supplementary Fig. S6f,g). Many P-body component proteins influence P-body formation itself^28,29,41^. In parallel with this, several studies have reported that APOBEC3G exists in P-bodies and that its overexpression decreases P-body formation^35–38^. APOBEC3G was originally characterized as an anti-HIV infection molecule in P-bodies but further research revealed that this function is independent of its localization to P-bodies^38,42^. Thus, APOBEC3G might also regulate P-body formation independently from its localization in P-bodies. Future studies will reveal the detailed mechanism through which APOBEC3G controls P-body formation.

Although EXOSC9 depletion effectively increased APOBEC3G protein levels in MDA-MB-231 cells, this increase was relatively modest. To address whether this moderate increase in APOBEC3G was sufficient to impact P-body formation and stress resistance, we prepared MDA-MB-231 cells expressing V5-tagged APOBEC3G at a level comparable to endogenous APOBEC3G. Results showed that exogenous APOBEC3G-expressing MDA-MB-231 cells reduced P-body formation and stress resistance compared with control cells (Supplementary Fig. S7), thus demonstrating that a moderate increase in APOBEC3G protein is sufficient to affect P-body formation and stress resistance. However, the difference in P-body formation and stress resistance between control and exogenous APOBEC3G-expressing MDA-MB-231 cells was smaller than that observed between control and EXOSC9-depleted MDA-MB-231 cells, as well as between control and APOBEC3G-depleted EXOSC9 KD MDA-MB-231 cells. Therefore, EXOSC9-depletion may serve to enhance the effect elicited by APOBEC3G on P-body formation and stress resistance.

In addition to P-bodies, cells have other RNA–protein granules such as SGs and cell-type specific granules^1,2^. EXOSC9 depletion did not affect arsenite-induced SG formation in HeLa cells (Fig. 4e–g). Thus, decreased P-body formation induced by EXOSC9 depletion is likely attributed to P-body directional regulation by the RNA exosome complex rather than a non-specific effect on RNA–protein granule formation caused by aberrant RNA metabolism. P-bodies were originally considered sites of RNA degradation because of the abundant existence of molecules related to 5′-3′ mRNA degradation such as XRN1, but accumulating reports suggest that P-bodies are sites for mRNA storage apart from translation machineries^29^. Comprehensive analyses of P-body constituent molecules by fluorescence-associated sorting of RNA–protein granules have revealed that more proteins related to functional regulation than constitutive proteins exist in P-bodies^43^. Meanwhile, the RNA exosome complex preferentially targets short-lived mRNAs with AREs, which usually encode cytokines and transcriptional factors and are transcribed in response to stimuli^15,39,40,44^. Coupling the RNA exosome complex-mediated degradation of mRNAs for outside stimuli with the storage of mRNAs for the regulation of cellular function might support the adaptation of such cells to stress conditions. P-bodies have also been reported to promote epithelial–mesenchymal transition (EMT) in cancer cells^45^. Since EMT promotes drug resistance in such cells^46,47^, it might also partially contribute to the EXOSC9-mediated stress resistance in cancer cells observed in this study.

Previously, depletion of one exosome component, save for EXOSC9, has been shown to affect protein levels of other exosome components^23,25^. Here, we confirmed that depletion of EXOSC2/EXOSC4 also impacted expression of other exosome components, including EXOSC9 in MDA-MB-231 cells (Fig. 4a). Interestingly, EXOSC9-depletion caused a moderate accumulation of PROMPTs, while decreasing P-body formation and stress resistance, to levels comparable to that observed following EXOSC2/EXOSC4 depletion, without significantly affecting the expression of other exosome components or cell growth under normal culture conditions. Thus, EXOSC9 might have unique roles in P-body formation and stress resistance. Moreover, the resulting phenotypes of EXOSC2/EXOSC4-depletion in P-body formation and stress resistance may in fact be related to reduced EXOSC9 protein expression. Thus, further investigation is required to elucidate the precise contribution made by each exosome component to P-body formation and stress resistance.

In conclusion, we show that EXOSC9 depletion attenuates stress resistance and P-body formation in cancer cells and that higher EXOSC9 activity correlates with poor prognosis for patients with some types of cancers. Thus, drugs targeting the activity of the RNA exosome complex or EXOSC9 might also be useful for these cancers.

## Materials and methods

### Cell culture

The human breast cancer cell lines MDA-MB-231 and MCF-7 and the cervical cancer cell line HeLa were purchased from the American Type Culture Collection (Manassas, VA, USA). Cells were cultured in Dulbecco’s Modified Eagle Medium (Thermo Fisher Scientific, Waltham, MA, USA) containing 10% fetal bovine serum, 100 units/mL penicillin, and 100 µg/mL streptomycin (hereinafter called “normal media”) at 37 °C in a humidified incubator with 5% CO_2_.

### Vector construction

The DNA versions of the targeted shRNA sequences (Supplementary Table S3) were subcloned into the pENTR/U6 TOPO vector (Thermo Fisher Scientific) before being transferred via recombination into the lentivirus vector, pLenti6 BLOCK iT (Thermo Fisher Scientific). Human *EXOSC9* and *APOBEC3G* cDNA was amplified from MDA-MB-231 cells by RT-PCR. Constructs expressing mutant EXOSC9 with R104A, R108A, and R111A substitutions in the RNA-binding motif (MUT EXOSC9) were prepared using a PCR-based method. These cDNAs were then subcloned into pENTR/D-TOPO (Thermo Fisher Scientific) before being transferred into the lentivirus vector pLenti6, as described previously^24,48^. The lentiviral vectors were generated and used according to the manufacturer’s instructions.

### siRNA knockdown

Knockdown by siRNA was carried out by using Lipofectamine RNAiMAX (Thermo Fisher Scientific) as previously described^49^. The sequence of the siRNA for each gene is described in Supplementary Table S3.

### Cell growth assay

Cells (2.5 × 10^4^/well) were seeded in 24-well plates. The next day, culture media were replaced with normal media, serum-free media, or normal media containing cisplatin (40 μM; FUJIFILM Wako Pure Chemical Corporation, Osaka, Japan), tunicamycin (10 μg/mL; Merck, Kenilworth, NJ, USA), or H_2_O_2_ (100 μM; FUJIFILM Wako Pure Chemical Corporation) and cultured at 37 °C in a humidified CO_2_ incubator for 24 h. The cells were then counted using a hemocytometer. To detect cell death, cells were treated with cisplatin or H_2_O_2_ for 24 or 6 h, respectively, and stained with Hoechst33342 (1 μg/mL; Merck) for all cells and Ethidium Homodimer III (EthD-III; 2 μM; PromoCell, Heidelberg, Germany) for dying or dead cells 15 min before observation under a fluorescent microscope (Keyence, Osaka, Japan).

### Western blot analysis

Cell lysate were prepared and subjected to western blotting as previously described^48^. The detailed information on antibodies used in this study is listed in Supplementary Table S4.

### RNA isolation, reverse transcription, and quantitative PCR

Total RNA was isolated from cells using TRIzol (Thermo Fisher Scientific) and subjected to reverse transcription (RT) using Superscript III (Thermo Fisher Scientific) and random primers as previously described^48^. For the analysis of mRNA half-life, cells were treated with actinomycin D (5 μg/mL; Merck) for indicated times before RNA isolation. The RT products were then analyzed by quantitative PCR (qPCR) using a 7500 real-time PCR system (Thermo Fisher Scientific) and SYBR Green PCR Master Mix (Thermo Fisher Scientific), with the specific primers provided in Supplementary Table S5. The PCR products were sequenced, and homogeneity was confirmed through the dissociation temperature monitoring of SYBR Green I fluorescence.

### RNA immunoprecipitation

Cells (2 × 10^7^ cells per immunoprecipitation) were lysed in lysis buffer (150 mM NaCl, 50 mM Tris pH 8.0, 1% NP-40, 0.5 mM dithiothreitol, and 100 U/mL RNasin PLUS RNase inhibitor (Promega, Madison, WI, USA)), and centrifuged at 20,000 × *g* for 30 min at 4 °C. Supernatants were collected and incubated with beads conjugated to anti-V5 antibodies (A7345, Sigma-Aldrich, St Louis, MO, USA) for 3 h at 4 °C. Beads were washed with lysis buffer five times, and bound RNA was isolated using TRIzol, and subjected to RT-qPCR.

### Tumor growth assay

All experimental protocols were approved by the Animal Care and Use Committee for The Institute of Medical Science, University of Tokyo, and conducted according to the institutional ethical guidelines for animal experiments and the safety guidelines for gene manipulation experiments. The tumorigenicity of cells was examined using 6-week-old female BALB/c nude mice (Clea Japan, Tokyo, Japan) as previously described^48,49^. Briefly, 2 × 10^6^ MDA-MB-231 cells were injected subcutaneously into the dorsal side of mice. Subsequently, the implanted tumors were blindly measured with calipers on the indicated days and their volumes were calculated using the formula V = (L × W^2^) / 2, where V is the volume (mm^3^), L is the largest tumor diameter (mm), and W is the smallest tumor diameter (mm).

### Immunostaining of cells

Immunostaining was performed using specific antibodies (Supplementary Table S4) as previously described^50^. To detect SGs, cells were treated with arsenite (100 μM; Merck) for 30 min before immunostaining. Cells were counterstained with Hoechst33342, washed five times with PBS, mounted on slides, and imaged by confocal microscopy (Carl Zeiss, Oberkochen, Germany and Olympus, Tokyo, Japan).

### Frozen sections and immunostaining

Frozen sections of tumor tissues at day 25 were prepared and subjected to immunostaining using specific antibodies (Supplementary Table S4) as previously described^51,52^. The nuclei were counterstained with Hoechst 33342, and the sections were observed by confocal microscopy (Olympus).

### RNA-seq

Total RNA was isolated from cells (three biological replicates per group) using the RNeasy mini plus kit (Qiagen, Hilden, Germany) according to manufacturer’s instructions. From 1 μg of total RNA, strand-specific RNA-Seq libraries were prepared with the TruSeq Stranded mRNA HT Sample Prep Kit (Illumina, San Diego, CA, USA), following the manufacturer’s instructions. Next, 36-bp single-end sequencing of the libraries was performed using HiSeq3000 (Illumina). To remove reads derived from rRNA, the reads were first aligned to rRNA using bowtie2.2.4^53^. Then, unaligned reads were realigned to the human reference genome hg19 using tophat2.0.13 with the–no-coverage-search option and guide of RefSeq transcripts^54^. We used only reads that were uniquely mapped to the human genome, allowing a 2-base mismatch, and with a quality of mapping greater than 50. Using in-house perl scripts, we counted reads mapped to each RefSeq gene and calculated RPKM (reads per kilo-base million) values as the gene expression value. Gene expression between mock and WT EXOSC9-expressing cells was analyzed using the DESeq. 2 R package (1.26.0)^30^, and genes with a Benjamini-Hochberg adjusted p-value <0.05 were considered to have significantly different expression levels. RNA-seq data were deposited in the DDBJ databases (https://www.ddbj.nig.ac.jp/index-e.html) under Accession number: DRA009981.

### Informatics analysis

To identify wild-type (WT) EXOC9-dependent genes based on RNA-seq data, the WT-specific top 5% of underrepresented or overrepresented genes was stratified among the three conditions as follows: WT, mock, and mutant (MUT). To validate our experimental results in human clinical samples based on EXOC9-target genes, we performed Kaplan–Meier analysis of a compiled clinical dataset from The Cancer Genome Atlas (TCGA) database via NCI Genomic Data Commons (GDC; downloaded at Apr. 2019). We independently analyzed overall survival rates for the two datasets respectively based on EXOC9-target genes. We derived a gene expression signature of 343 EXOSC9-target genes for each sample based on principal component analysis, and stratified samples with respect to the expression signatures of EXOSC9-target genes; the upper/lower quartile for the TCGA dataset were used to characterize the two groups as “high” and “low” based on a value greater than the median raw expression value; we then performed Kaplan–Meier analysis based on these data.

### Statistical analysis

We compared two groups using a two-sided t test or the Mann–Whitney U test using GraphPad Prism software (GraphPad Software, Inc., La Jolla, CA, USA). For Kaplan–Meier analyses, the log-rank test was performed using the R package “survival” and “survminer”.

## Supplementary information

Supplementary information.

## Data Availability

All additional data from the experiments are provided in Supplementary Information.
