# Supplementary material for: EXOSC9 depletion attenuates P-body formation, stress resistance, and tumorigenicity of cancer cells
Source: Sci Rep. 2020 Jun 9;10:9275. doi: 10.1038/s41598-020-66455-2 (PMC7283315; doi:10.1038/s41598-020-66455-2)
Supplement: Supplementary file 1 — Supplementary information. [file 41598_2020_66455_MOESM1_ESM.pdf]

Supplementary Information

EXOSC9 depletion attenuates P-body formation, stress resistance, and tumorigenicity of cancer cells

Seiko Yoshino[1#], Yusuke Matsui[2#], Yuya Fukui[3], Masahide Seki[4], Kiyoshi Yamaguchi[5], Akane Kanamori[3], Yurika Saitoh[3, 6], Teppei Shimamura[7], Yutaka Suzuki[4], Yoichi Furukawa[5], Shuichi Kaneko[8], Motoharu Seiki[9], Yoshinori Murakami[1], Jun-ichiro Inoue[3], and Takeharu Sakamoto[8*]

[1]Division of Molecular Pathology, the Institute of Medical Science, The University of Tokyo, Shirokanedai, Minato-ku, Tokyo, Japan

[2]Biomedical and Health Informatics Unit, Department of Integrated Health Science, Nagoya University Graduate School of Medicine, Daiko-Minami, Higashi-ku, Nagoya, Aichi, Japan

[3]Division of Cellular and Molecular Biology, the Institute of Medical Science, The University of Tokyo, Shirokanedai, Minato-ku, Tokyo, Japan

[4]Department of Computational Biology and Medical Sciences, Graduate School of Frontier Sciences, the University of Tokyo, Chiba, Japan

[5]Division of Clinical Genome Research, the Institute of Medical Science, The University of Tokyo, Shirokanedai, Minato-ku, Tokyo, Japan

[6]Center for Medical Education, Teikyo University of Science, Senjusakuragi, Adachi-ku, Tokyo, Japan

[7]Division of Systems Biology, Nagoya University Graduate School of Medicine, Tsurumai-cho, Nagoya, Japan

[8]Department of System Biology, Institute of Medical, Pharmaceutical and Health Sciences, Kanazawa University, Takaramachi, Kanazawa, Ishikawa, Japan

[9]Division of Cancer Cell Research, the Institute of Medical Science, The University of Tokyo, Shirokanedai, Minato-ku, Tokyo, Japan

[#] These authors equally contributed to this study.

[*]**Address correspondence to:** Takeharu Sakamoto, Ph. D., 13-1 Takara-machi, Kanazawa, Ishikawa 920-8640, Japan. Tel: +81-76-265-2235; Fax: +81-76-234-4250; E-mail: t-saka@staff.kanazawa-u.ac.jp

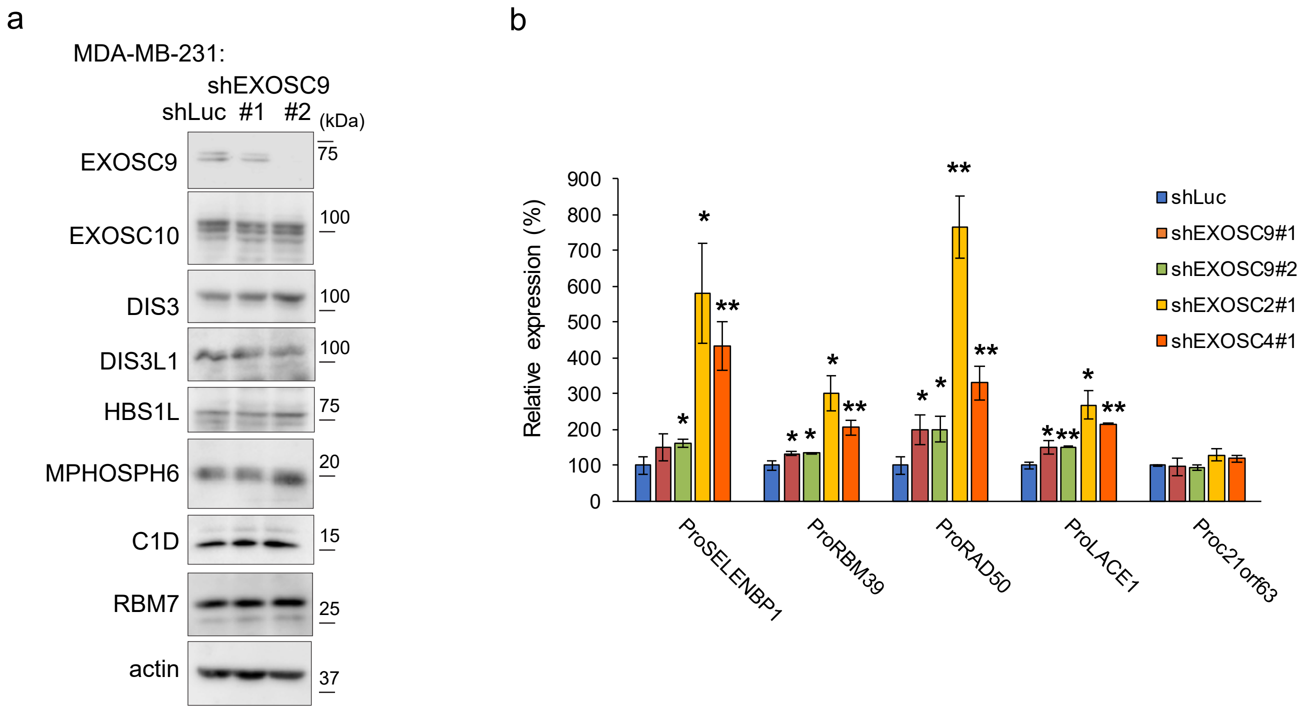

Supplementary Figure S1. EXOSC9 depletion affects RNA exosome activity

(**a**) Expression of the RNA exosome-associated 5′–3′ exoribonucleases and exosome cofactors in control (shLuc) and EXOSC9-depleted (shEXOSC9#1, #2) MDA-MB-231 cells.

(**b**) Expression of promoter upstream transcripts (PROMPTs; Pro) of indicated genes in control (shLuc), EXOSC9-depleted (shEXOSC9#1, #2), EXOSC2-depleted (shEXOSC2#1), and EXOSC4-depleted (shEXOSC4#1) MDA-MB-231 cells. n = 3. Data represent mean ± SD. * $p < 0.05$, ** $p < 0.01$ as determined by Student' s t-tests. Note that PROMPTs, save for Proc21orf63, were accumulated in EXOSC9-, EXOSC2-, and EXOSC4-depleted cells.

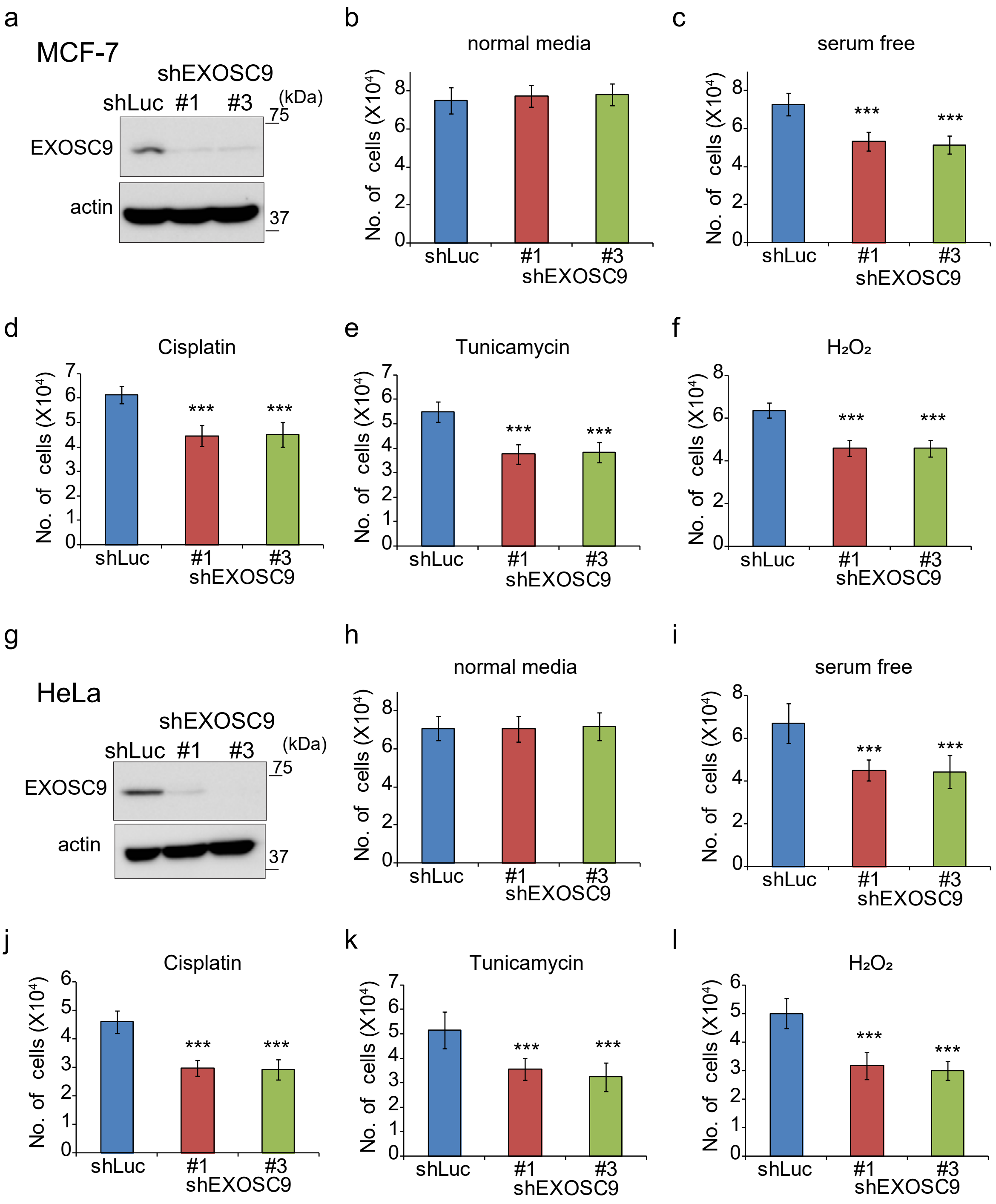

Supplementary Figure S2. EXOSC9 is necessary for stress resistance in MCF-7 and HeLa cells.
(**a, g**) Expression of EXOSC9 in control (shLuc) and EXOSC9-depleted (shEXOSC9#1, #3) MCF-7 (**a**) and HeLa cells (**g**).
(**b–f, h–l**) Cell number of control and EXOSC9-depleted MCF-7 (**b–f**) and HeLa cells (**h-l**) cultured in normal media (**b, h**), serum free media (**c, i**), or normal media in the presence of cisplatin (40 μM) (**d, j**), tunicamycin (10 μg/ml) (**e, k**), or H_2O_2 (100 μM) (**f, l**) for 24 h.
In **b–f** and **h–l**, n = 9 from three independent experiments. Data represent mean ± SD. *** p < 0.001 by Student’ s t-test.

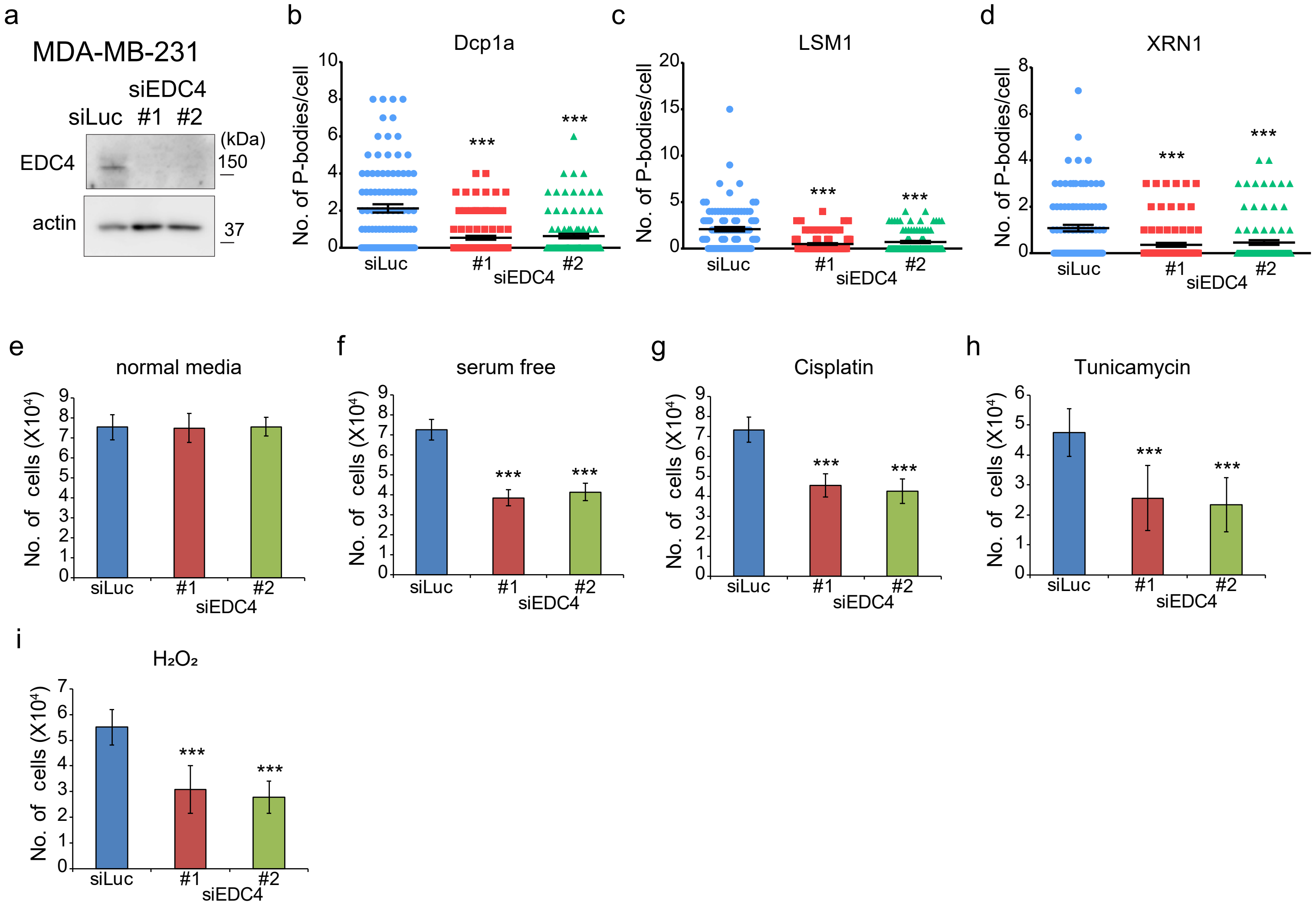

Supplementary Figure S3. EDC4 depletion attenuates P-body formation and stress resistance in MDA-MB-231 cells.
(**a**) Expression of EDC4 in control (siLuc) and EDC4-depleted (siEDC4#1, #2) MDA-MB-231 cells.
(**b-d**) The number of indicated P-body marker-positive granules in a cell was counted. n = 100 per group. Data represent mean ± SEM. *** $p < 0.001$ by Mann-Whitney U-test.
(**e-i**) Cell number of control and EDC4-depleted MDA-MB-231 cells cultured under indicated conditions for 24 h. n = 9 from three independent experiments. Data represent mean ± SD. *** $p < 0.001$ by Student’ s t-test.

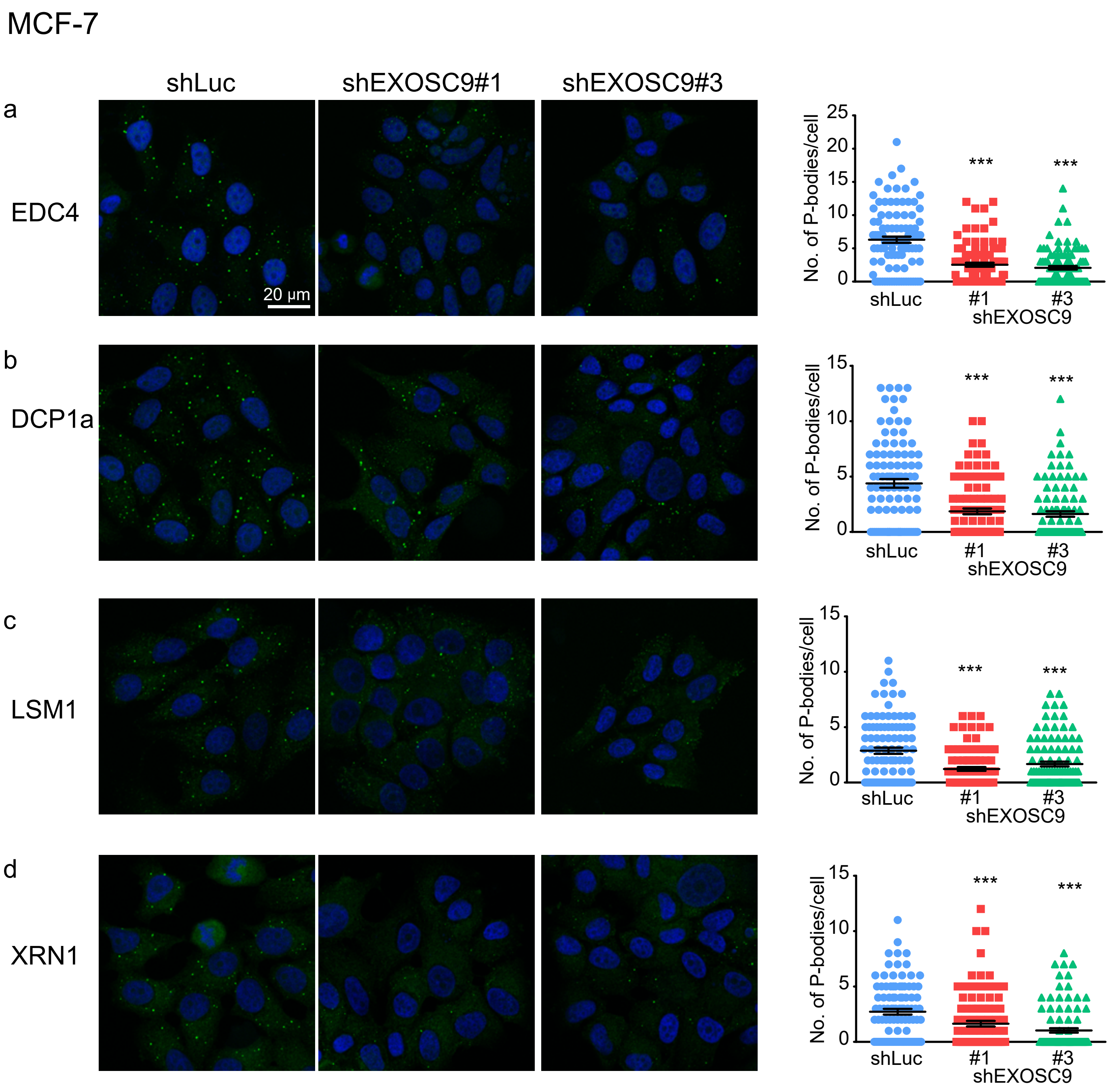

Supplementary Figure S4. EXOSC9 is necessary for P-body formation in MCF-7 cells.
(**a–d**) Immunostaining for the P-body markers EDC4 (**a**), DCP1a (**b**), LSM1 (**c**), and XRN1 (**d**) in control (shLuc) and EXOSC9-depleted (shEXOSC9#1, #3) MCF-7 cells. (Left) representative photos. (Right) the number of indicated P-body marker-positive granules in a cell was counted. n = 100 per group. Data represent mean ± SEM. *** $p < 0.001$ by Mann-Whitney U-test.

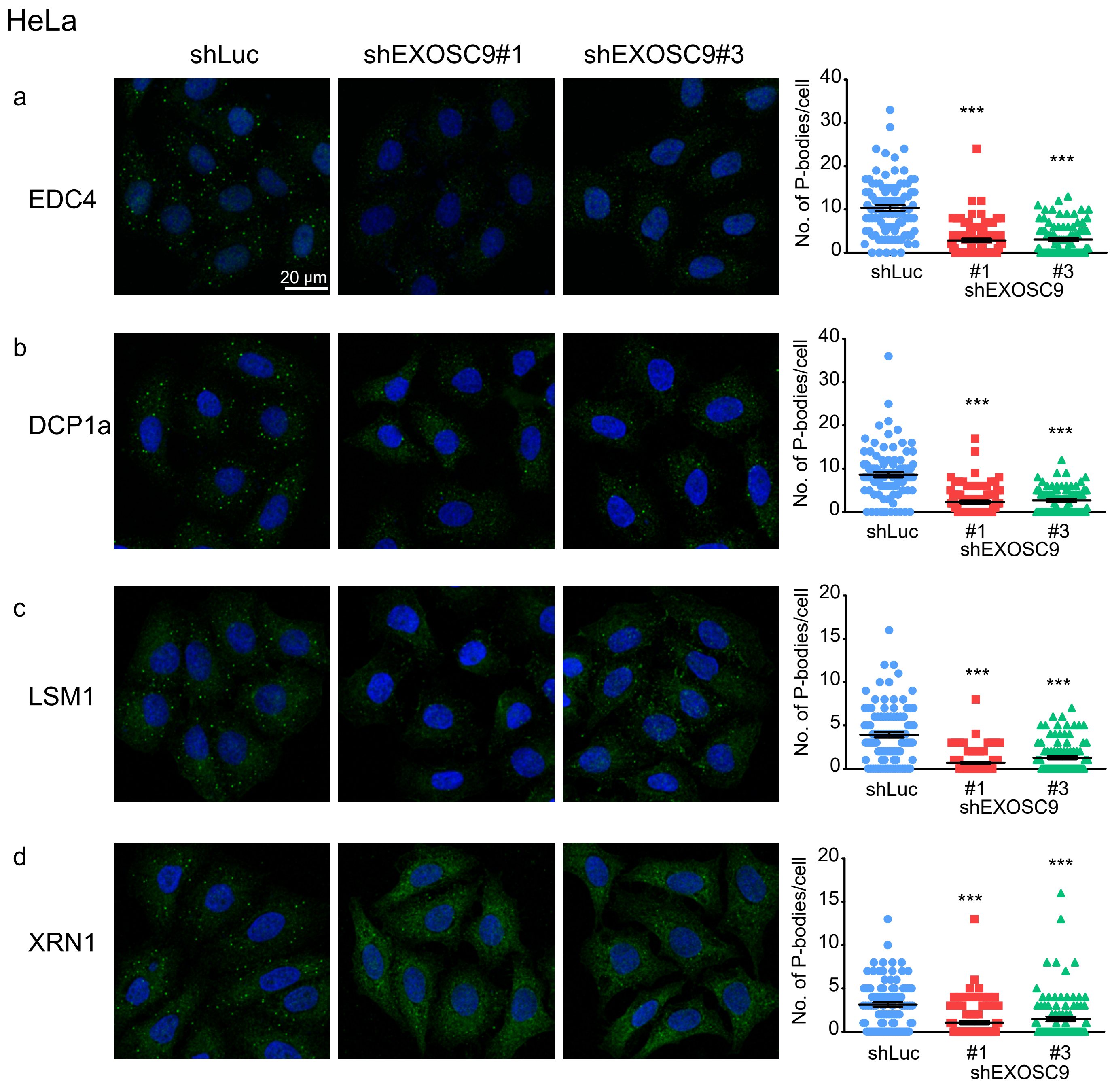

Supplementary Figure S5. EXOSC9 is necessary for P-body formation in HeLa cells.
(**a–d**) Immunostaining for the P-body markers EDC4 (**a**), DCP1a (**b**), LSM1 (**c**), and XRN1 (**d**) in control (shLuc) and EXOSC9-depleted (shEXOSC9#1, #3) HeLa cells. (Left) representative photos. (Right) the number of indicated P-body marker-positive granules in a cell was counted. n = 100 per group. Data represent mean ± SEM. *** $p < 0.001$ by Mann-Whitney U-test.

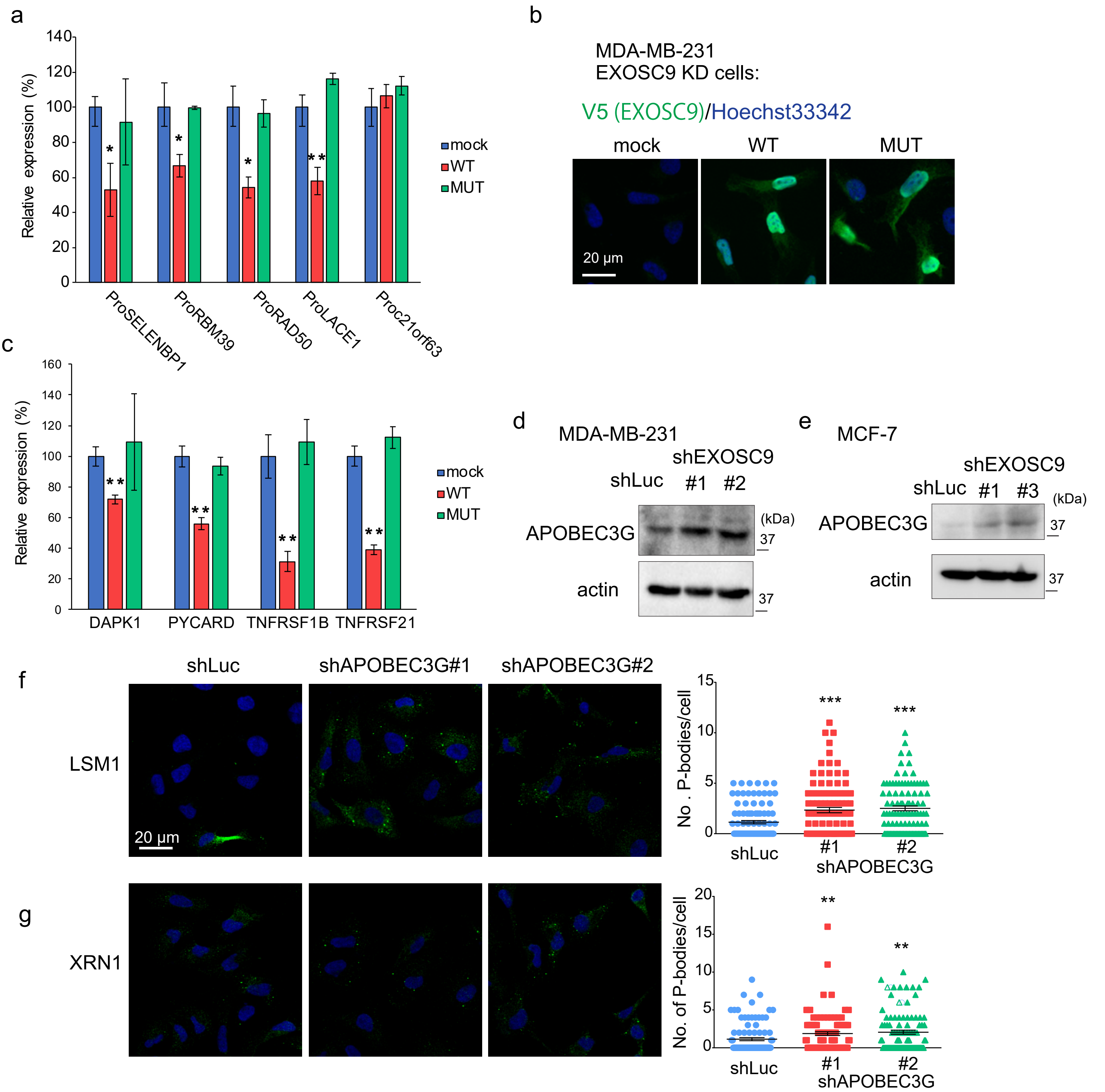

Supplementary Figure S6. APOBEC3G depletion restores P-body formation in EXOSC9-depleted MDA-MB-231 cells.
(**a**) Expression of PROMPTs in mock, WT and MUT EXOSC9-expressing EXOSC9 KD MDA-MB-231 cells.
(**b**) Immunostaining of V5-tagged wild-type (WT) and mutant (MUT) EXOSC9 in EXOSC9 knockdown (KD) cells.
(**c**) Expression of stress/cell death-related genes in mock, WT and MUT EXOSC9-expressing EXOSC9 KD MDA-MB-231 cells.
(**d**) APOBEC3G protein expression in control (shLuc) and EXOSC9-depleted (shEXOSC9#1, #2) MDA-MB-231 cells.
(**e**) APOBEC3G protein expression in control (shLuc) and EXOSC9-depleted (shEXOSC9#1, #3) MCF-7 cells.
(**f, g**) Immunostaining of P-body markers LSM1 (**f**), and XRN1 (**g**) in control (shLuc) and APOBEC3G-depleted (shAPOBEC3G#1, #2) EXOSC9 KD MDA-MB-231 cells. (Left) representative photos. (Right) the number of indicated P-body marker-positive granules in a cell was counted. n = 100 per group. Data represent mean ± SEM. ** $p < 0.01$, *** $p < 0.001$ by Mann-Whitney U-test.
In **a** and **c**, n = 3. Data represent mean ± SD. * $p < 0.05$, ** $p < 0.01$ as determined by Student' s t-test.

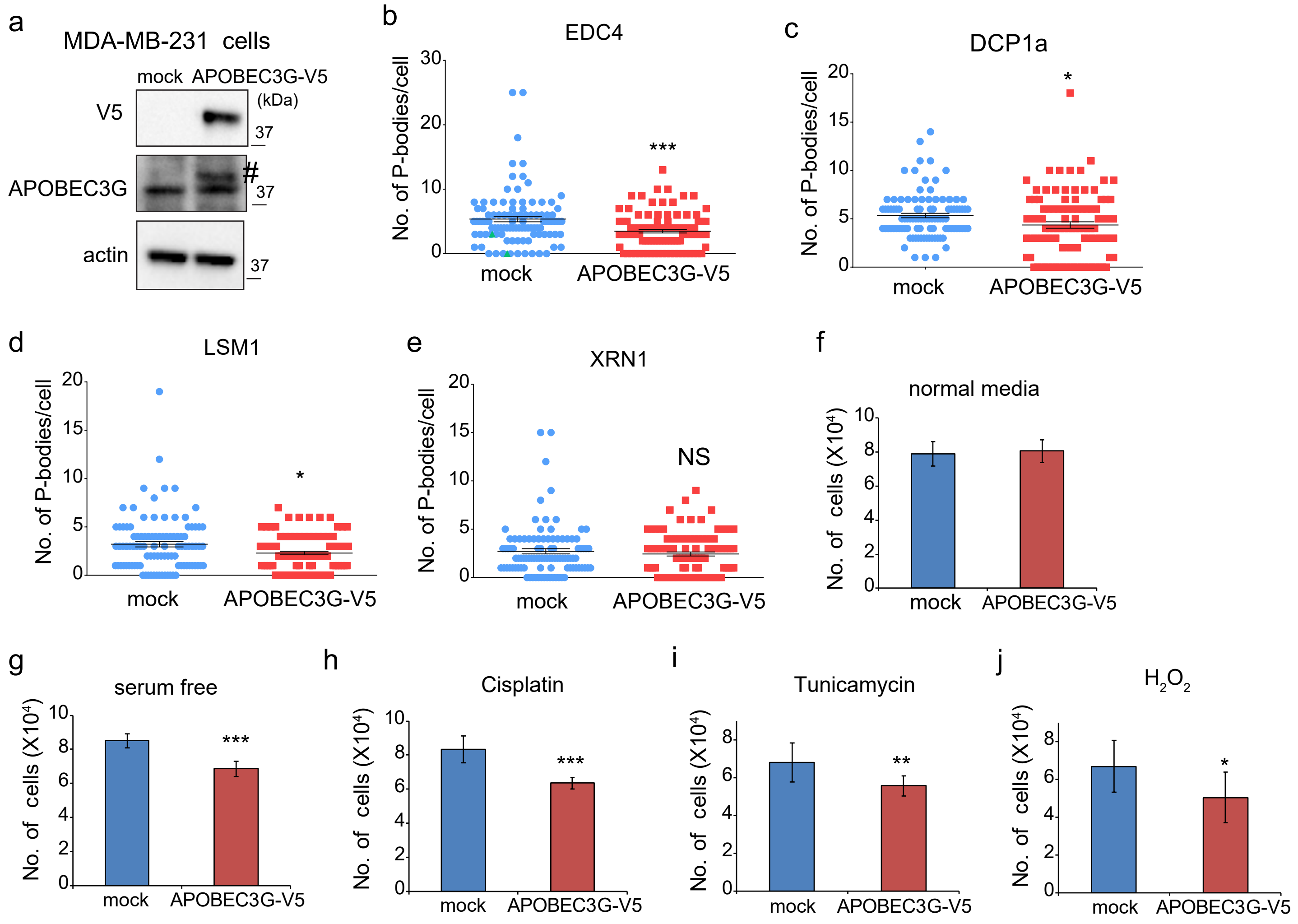

Supplementary Figure S7. Moderate expression of exogenous APOBEC3G affects P-body formation and stress resistance in MDA-MB-231 cells

(**a**) Expression of endogenous and exogenous APOBEC3G in mock and V5-tagged APOBEC3G-expressing MDA-MB-231 cells. # indicates V5-tagged exogenous APOBEC3G.

(**b-e**) Indicated P-body markers were stained in mock and V5-tagged APOBEC3G expressing MDA-MB-231 cells. The number of P-body marker foci in a cell was counted. n = 100. Data represent mean ± SEM. * $p < 0.05$, *** $p < 0.001$ as determined by Mann-Whitney U-test. NS, not significant.

(**f–j**) APOBEC3G depletion restored cell proliferation under indicated stress conditions in EXOSC9 KD MDA-MB-231 cells. n = 9 from three independent experiments. Data represent mean ± SD. * $p < 0.05$, ** $p < 0.01$, *** $p < 0.001$ as determined by Student' s t-tests.

Supplementary Figure S8

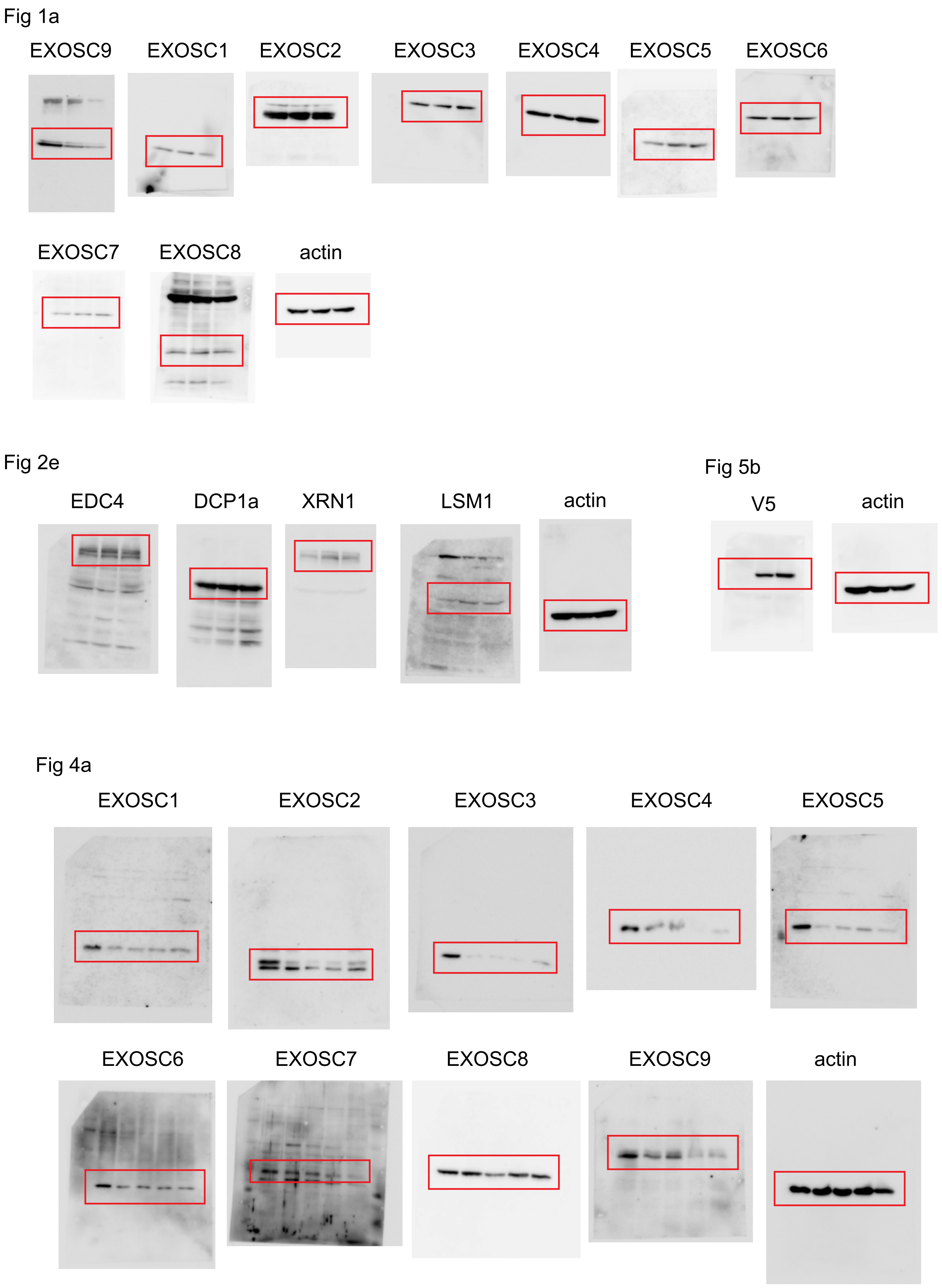

Fig 6c

APOBEC3G actin

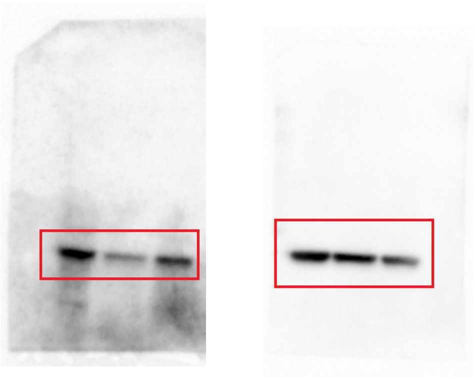

Fig 6e

APOBEC3G actin

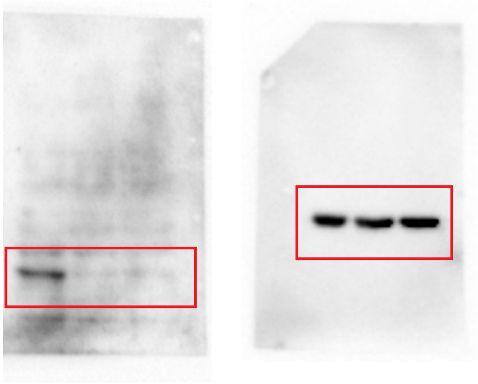

Fig. S1a

EXOSC9 EXOSC10 DIS3 DIS3L HBS1L MPHOSPH6 C1D RBM7 actin

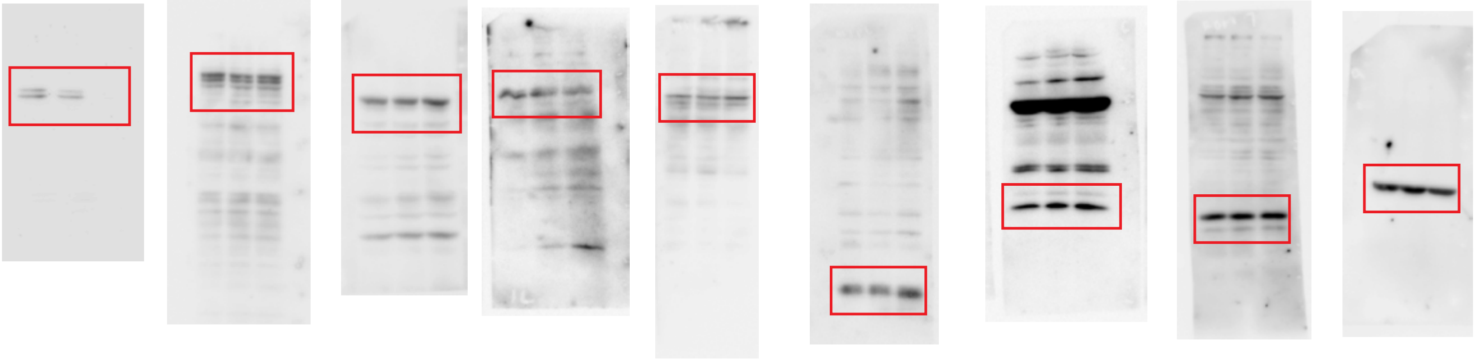

Fig. S2a

EXOSC9 actin

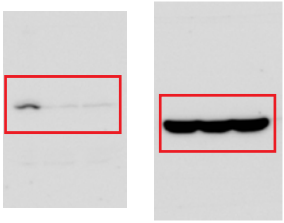

Fig. S2g

EXOSC9 actin

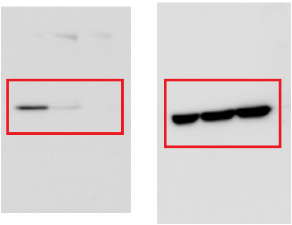

Fig. S3a

EDC4 actin

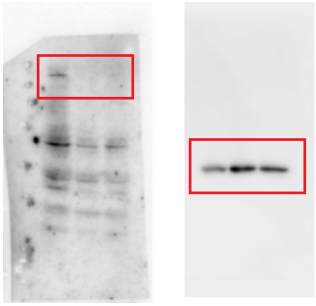

Fig. S6d

APOBEC3G actin

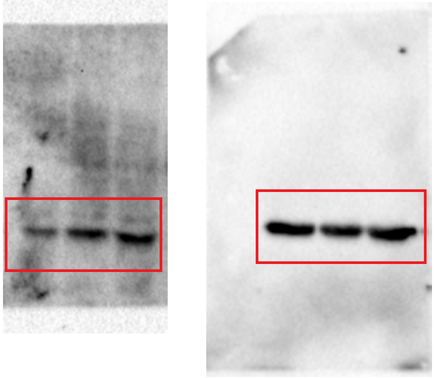

Fig. S6e

APOBEC3G actin

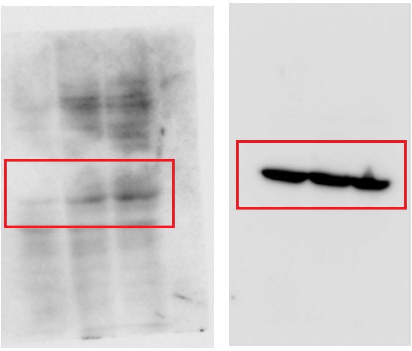

Fig. S7a

V5 APOBEC3G actin

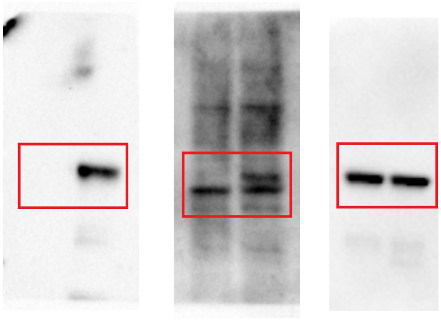

Supplementary Figure S8. Uncropped immunoblots shown in figures.

Rank	gene	mock#1 (rpkm)	mock#2 (rpkm)	mock#3 (rpkm)	WT#1 (rpkm)	WT#2 (rpkm)	WT#3 (rpkm)	MUT#1 (rpkm)	MUT#2 (rpkm)	MUT#3 (rpkm)	log2FC (WT/mock)	log2FC (MUT/mock)	adjusted p value (mock vs WT)
1	CTAG2	0.08	0.06	0.05	51.56	33.69	32.93	0.06	0.02	0.09	9.28077077	-0.160464672	4.74E-63
2	GRM8	0.03	0.02	0.01	0.08	0.17	0.16	0.01	0.02	0.02	2.772589504	-0.263034406	0.001885215
3	TEK	0.01	0.02	0.01	0.06	0.09	0.07	0.02	0.01	0.01	2.459431619	0	0.016068817
4	COX7B2	0.29	0.11	0.33	1.04	1.17	1.8	0.23	0.27	0.38	2.457633868	0.26960706	0.000199703
5	PRKG1	0.05	0.04	0.03	0.16	0.24	0.24	0.04	0.05	0.06	2.415037499	0.321928095	1.90E-08
6	KCNH5	0.13	0.05	0.04	0.24	0.59	0.34	0.02	0.05	0.07	2.410933101	-0.652076697	0.00024599
7	GPR87	0.33	0.3	0.28	1.19	1.54	1.88	0.36	0.34	0.4	2.3408283	0.273565073	1.13E-11
8	PDGFB	0.48	0.46	0.53	2.81	2.47	1.93	0.72	0.59	0.74	2.294183104	0.479807755	1.96E-25
9	CST1	13.76	14.53	15.48	95.68	58.72	50.09	16.39	14.64	12.98	2.224016005	0.007888988	5.32E-17
10	GTSF1	5.09	4.67	5.17	26.6	18.41	21.34	7.3	6.23	6.03	2.1518823	0.389692205	2.18E-29
11	NCAM2	0.11	0.05	0.07	0.29	0.37	0.36	0.1	0.06	0.07	2.148863386	0	3.43E-08
12	MMP9	0.62	0.53	0.58	3.52	2.27	1.61	0.95	0.89	0.72	2.096753233	0.565371772	1.96E-09
13	EYA1	0.02	0.03	0.02	0.07	0.12	0.1	0.04	0.03	0.03	2.050626073	0.514573173	0.008551394
14	PMEPA1	1.6	1.76	1.66	6.73	6.94	6.15	1.92	1.98	1.98	1.981197693	0.228128791	2.12E-65
15	CST4	9.52	8.7	9.18	51	30.14	26.8	9.29	7.74	7.94	1.977981794	-0.133980072	1.04E-12
16	KRTAP2-4	5.06	4.3	5.66	23.52	17.5	16.6	7.1	6.06	6.66	1.939684848	0.40007215	6.08E-20
17	KRTAP2-1	3.86	3.91	4.99	20.51	14.43	12.05	6.94	5.62	6.17	1.880725439	0.55372257	7.38E-13
18	CST2	3.94	4.39	4.32	20.24	13.78	11.38	5.11	4.65	3.05	1.843554913	0.018133091	4.24E-12
19	ZFPM2	0.19	0.28	0.14	0.5	0.8	0.88	0.2	0.21	0.34	1.837446987	0.298081353	2.14E-06
20	DHRS3	0.26	0.19	0.16	1.05	0.59	0.53	0.13	0.22	0.2	1.830813895	-0.149377624	0.000553958
21	AKR1C1	0.5	0.57	0.55	2.11	1.56	1.61	0.79	0.65	0.63	1.704544116	0.353636955	5.97E-09
22	ARHGAP44	0.28	0.31	0.31	0.82	0.98	0.96	0.34	0.25	0.34	1.61667136	0.047305715	6.99E-14
23	AKR1C2	0.16	0.13	0.17	0.59	0.33	0.49	0.21	0.25	0.21	1.615989396	0.542527234	2.41E-05
24	ADTRP	0.76	0.99	1.08	2.88	2.84	2.87	1.37	1.2	1.18	1.601856078	0.406088543	1.61E-15
25	VNN1	0.35	0.43	0.4	1.22	1.06	1.29	0.57	0.5	0.62	1.597137215	0.518236387	4.87E-14
26	MAP3K7CL	0.89	0.71	0.75	2.19	2.09	2.54	1.04	1.01	1.26	1.537110982	0.49417046	2.40E-17
27	AKR1C3	0.93	0.76	1.11	3.04	2.25	2.51	1.01	1.22	1.02	1.478047297	0.215012891	1.27E-09
28	TNFSF15	0.46	0.59	0.44	1.18	1.51	1.41	0.57	0.78	0.61	1.460311579	0.395541324	2.00E-13
29	MATK	0.18	0.26	0.21	0.75	0.5	0.53	0.28	0.35	0.27	1.453365618	0.469485283	0.000299853
30	PRAME	4.47	3.98	4.3	11.87	11.3	11.37	5.91	5	5.81	1.437770836	0.3910776	4.84E-42
31	NOS3	0.16	0.17	0.2	0.58	0.44	0.39	0.25	0.23	0.22	1.411630898	0.401362562	6.64E-06
32	RFPL4AL1	4.14	4.2	4.07	12.7	9.81	9.96	2.97	2.75	2.59	1.387604269	-0.578582733	2.83E-14
33	UCN2	20.45	22.06	22.26	72.48	51.94	44.9	26.79	24.34	21.11	1.386354745	0.157472149	9.91E-11
34	KRT34	0.29	0.37	0.22	1.05	0.49	0.74	0.42	0.47	0.3	1.373458396	0.435386145	0.01224426
35	MAGEA11	1.31	1.21	1.15	3.24	2.88	3.31	1.82	1.73	1.58	1.361477708	0.483178763	5.29E-15
36	MYO16	0.19	0.16	0.16	0.45	0.37	0.47	0.21	0.16	0.2	1.338801913	0.160464672	1.01E-07
37	RFPL4A	4.55	4.47	4.59	12.75	10.31	11.06	3.37	2.89	2.66	1.32595058	-0.609551452	8.77E-17
38	ARHGAP6	0.25	0.25	0.22	0.47	0.68	0.64	0.36	0.3	0.34	1.313890776	0.473931188	2.90E-07
39	TMEM204	0.94	0.92	0.74	2.55	1.86	2.03	1.14	0.78	0.9	1.308549065	0.117183539	1.39E-07
40	ANGPTL4	1.38	1.46	1.4	5.1	2.71	2.69	1.94	1.77	1.54	1.308253158	0.308253158	0.000178111
41	RND1	0.25	0.37	0.22	0.79	0.64	0.6	0.31	0.4	0.46	1.273018494	0.478047297	0.005641222
42	BHLHE40	8.91	9.06	8.49	21.48	22.91	19.01	9.8	10.72	9.64	1.260669779	0.188823367	2.90E-26
43	GABRB3	0.05	0.08	0.06	0.14	0.15	0.16	0.06	0.1	0.06	1.243925583	0.211504105	0.010304097
44	VCX3A	1.31	0.92	1.14	3.19	1.96	2.69	0.78	0.81	0.77	1.218105063	-0.513961732	0.000404281
45	TFF2	2.22	2.39	2.41	8	3.77	4.48	2.56	1.57	2.12	1.210896782	-0.167614841	0.002965771
46	FABP4	0.56	0.27	0.73	1.36	0.97	1.22	0.58	0.78	0.79	1.186272996	0.462790631	0.042418092
47	ZNF883	1.11	1.13	1.21	2.61	2.4	2.79	1.37	1.23	1.39	1.176877762	0.209792385	1.17E-11
48	AMH	3.82	3.35	4.23	11.54	6.81	6.93	4.87	4.14	4.55	1.148962639	0.250323354	7.73E-05
49	ACOT11	0.6	0.45	0.49	1.27	1.09	1.05	0.73	0.55	0.7	1.146841388	0.362570079	1.05E-06
50	VCX	2.52	2.03	2.99	6.3	4.96	5.4	2	1.41	1.52	1.143751972	-0.612976877	1.39E-06
51	PTHLH	0.28	0.26	0.35	0.57	0.7	0.68	0.5	0.39	0.39	1.131596883	0.524266569	0.000640848
52	SHROOM4	0.05	0.06	0.05	0.08	0.13	0.14	0.06	0.06	0.06	1.129283017	0.169925001	0.018485977
53	SPTB	0.29	0.35	0.3	0.66	0.81	0.54	0.34	0.32	0.34	1.09646284	0.089267338	5.29E-06
54	DNAJC12	0.58	0.77	0.58	1.39	1.53	1.15	0.64	0.88	0.91	1.076427947	0.332355466	0.000943572
55	SERPINF2	0.43	0.48	0.45	1.17	0.92	0.75	0.66	0.52	0.41	1.062284278	0.225420114	0.001564986
56	RAB38	6.64	6.05	5.71	14.32	11.31	12.76	7.46	6.65	7.67	1.061024794	0.243298188	2.53E-11
57	ENG	6.48	7.08	7.81	17.4	13.8	12.48	10.34	9.26	9.47	1.031385948	0.443944163	2.88E-08
58	AKAP12	24.94	26.09	21.26	29.92	64.38	53.13	22.81	29.14	27.09	1.028162128	0.128786856	0.002689417
59	CDS1	0.13	0.21	0.18	0.29	0.38	0.39	0.24	0.23	0.15	1.027480736	0.253756592	0.010860132
60	HIC1	0.3	0.32	0.34	0.78	0.6	0.57	0.48	0.5	0.36	1.022367813	0.48112669	0.001255956
61	GRM5	0.16	0.18	0.16	0.21	0.42	0.38	0.17	0.17	0.14	1.014355293	-0.058893689	0.016248543
62	SIRPB1	0.22	0.21	0.15	0.39	0.36	0.41	0.19	0.29	0.21	1	0.250543462	0.004533277
63	MYOM3	0.14	0.14	0.08	0.23	0.26	0.23	0.12	0.14	0.15	1	0.187627003	0.015855845
64	HIST1H3C	1.63	1.39	1.56	3.94	2.81	2.37	2.28	1.67	1.83	0.993686226	0.335721894	0.02629922
65	PAX9	0.94	0.82	0.66	1.36	1.81	1.62	0.63	0.8	0.82	0.985018608	-0.105082046	7.90E-05
66	BCL11A	0.66	0.59	0.68	1.17	1.36	1.23	0.76	0.77	0.84	0.962131814	0.296286212	6.18E-10
67	ASGR1	0.97	1.18	1.18	2.65	2.04	1.78	1.45	1.49	1.29	0.958243535	0.345135486	0.001291302
68	SBSN	1.82	1.92	2.3	4.98	3.49	3.14	2.89	2.5	2.2	0.942747518	0.329551336	0.000827365
69	TPGS1	2.71	3.66	3.88	8.88	5.38	5.12	3.94	3.26	3.85	0.918944661	0.10842246	0.01173938
70	LTBP3	4.5	5.21	4.81	10.62	9.22	7.41	5.62	5.08	4.98	0.908214777	0.110884106	1.82E-06
71	PRR5	0.5	0.29	0.48	0.89	0.8	0.68	0.51	0.44	0.55	0.900058562	0.240134004	0.016248543
72	CDA	6.96	8.07	8.75	19.41	12.1	12.86	10.07	8.81	9.56	0.899835838	0.25817275	0.001300566
73	WNT10B	1.42	1.35	1.37	3.22	2.17	2.29	1.7	1.56	1.65	0.891475543	0.246092257	0.000481638
74	SLC25A21	0.41	0.34	0.33	0.73	0.53	0.74	0.45	0.42	0.35	0.888968688	0.175849835	0.004292891
75	BATF3	11.4	11.44	11.65	27.37	18.22	18.16	15.09	13.29	14.31	0.886247213	0.307720034	0.000185967
76	NDRG1	14.8	15.84	14.84	30.43	30.54	23.05	17.95	17.81	16.66	0.885500532	0.204885099	5.84E-08
77	VCX2	0.94	0.94	0.94	1.87	1.74	1.59	0.71	0.46	0.45	0.882816461	-0.79970135	0.014242784
78	CARD9	0.62	0.52	0.79	1.59	1.1	0.86	0.95	0.89	0.78	0.879218177	0.440965964	0.046711409
79	CD300C	0.49	0.49	0.59	0.85	1.19	0.83	0.34	0.39	0.27	0.870286178	-0.650764559	0.035942546

80	BRICD5	0.79	0.6	0.79	1.63	1.25	1.09	1.1	0.67	0.87	0.864810872	0.276209795	0.049397465
81	VCX3B	1.77	1.35	2.38	3.88	2.96	3.14	0.97	1.02	0.98	0.859608197	-0.888968688	0.011355619
82	FSTL3	5.28	5.23	6.08	12.16	9.04	8.9	6.32	5.02	5.52	0.859449601	0.02329065	1.70E-05
83	PIK3IP1	0.37	0.37	0.33	0.76	0.63	0.55	0.39	0.34	0.27	0.858445856	-0.097610797	0.019104258
84	TMEM145	1.81	2.11	2.09	4.5	3.24	3.14	2.59	2.32	2.47	0.856241661	0.296255825	0.000663763
85	GNB3	1.16	0.84	1.57	2.51	2.01	1.94	1.48	1.22	1.31	0.855610091	0.167678162	0.017602005
86	LRRC26	3.12	3.75	4.14	9.58	5.28	5.06	3.96	2.3	3.37	0.855403179	-0.193206766	0.033880969
87	TNNI3	1.33	1.27	1.37	3.04	2.37	1.75	2.02	1.4	1.62	0.85082058	0.344284726	0.033723155
88	TIMP1	123.73	123.82	134.68	302.8	196.16	188.82	159.6	140.3	135.32	0.847506149	0.18730384	0.000852037
89	PTPRR	1.13	0.91	1.11	1.89	1.77	2	1.19	1.21	1.21	0.845450224	0.196647008	2.40E-06
90	PRSS3	25.34	26.98	28.16	61.39	40.91	40.83	35.17	31.95	30.32	0.830623882	0.275883828	0.000426953
91	DHRS2	3.26	3.26	4.07	7.4	5.84	5.56	4.43	4.35	3.97	0.828030073	0.267794658	0.000140656
92	C4orf48	12.75	14.53	13.53	34.42	17.77	19.55	16.64	12.42	17.03	0.813855035	0.175531057	0.030979619
93	BMF	0.28	0.23	0.23	0.39	0.46	0.45	0.29	0.24	0.22	0.812914447	0.019365325	0.008513871
94	ADM5	1.49	1.95	2.1	4.37	2.65	2.66	2.22	2.31	2.23	0.805121071	0.28713727	0.028970797
95	TRIML2	2.46	2.2	2.38	4.06	4.11	4.07	3.14	3.15	3.23	0.797956224	0.435386145	2.76E-07
96	G0S2	183.85	182.68	199.16	399.42	286.4	294.91	253.38	221.34	234.79	0.793844342	0.32681135	3.09E-05
97	LBH	0.99	1.1	0.98	1.63	1.92	1.76	0.75	0.99	0.89	0.790473205	-0.223175856	3.21E-05
98	MTSS1	0.34	0.3	0.28	0.42	0.54	0.63	0.26	0.32	0.23	0.789320999	-0.183711953	0.013908554
99	TFPI2	16.44	15.74	17.86	29.67	28.01	28.73	19.35	18.91	19.87	0.788116492	0.216201119	3.76E-14
100	FOSB	1.02	1.05	1.01	2.01	1.77	1.51	1.71	1.25	1.15	0.780337371	0.416208043	0.000121842
101	CTSS	2.71	2.81	2.66	4.14	4.58	5.23	3.55	3.47	3.51	0.770092374	0.364332688	3.69E-07
102	MARCKSL1	54.14	50.44	59.47	111.16	82.61	85.15	64.69	58.55	61.37	0.765715793	0.17034511	3.92E-05
103	TAGLN3	9.21	9.33	11.25	20.34	15.24	15.06	12.19	11.1	11.73	0.765449281	0.23335096	0.00030725
104	RAC3	16.16	17.22	18.73	40.21	25.18	23.16	20.65	19.61	18.17	0.764932052	0.165149035	0.013673719
105	C19orf24	27.88	26.93	33.51	70.25	42.05	37.54	34.72	29.17	31.78	0.762610727	0.115326426	0.032580656
106	RBPMS	2.1	1.92	1.96	4	3.19	2.92	2.44	2.12	2.45	0.757565608	0.22926896	0.000149417
107	ARID3A	2.66	2.64	3.03	5.34	4.63	4.06	2.86	2.64	2.74	0.752126608	-0.015672158	4.71E-05
108	LINC00458	0.91	1.29	0.99	2.12	1.54	1.68	1.46	1.48	1.35	0.743283318	0.427421224	0.0330059
109	PRR22	1.73	2.01	2.19	4.46	2.88	2.58	2.66	2.49	2.54	0.742308016	0.374951493	0.046614469
110	VGF	3.23	2.96	3.49	6.87	4.63	4.67	3.83	3.48	3.43	0.740240726	0.149915041	0.004285166
111	MAGEB2	1.12	1.11	1.01	1.71	1.82	1.87	1.49	1.58	1.48	0.736965594	0.489872732	0.002419228
112	APC2	0.5	0.47	0.5	0.95	0.77	0.72	0.5	0.46	0.46	0.731064993	-0.049925225	0.000310951
113	ABHD17A	40.14	42.17	46.94	97.44	58.62	58.42	50.48	39.98	44.27	0.730678844	0.059906847	0.017484275
114	PNPLA7	0.18	0.25	0.24	0.39	0.38	0.34	0.24	0.17	0.22	0.728326676	-0.088809267	0.048529823
115	CISH	0.73	0.88	0.71	1.55	1.2	1.09	1.01	0.92	1.02	0.726981506	0.346590149	0.021635201
116	GBP5	1.19	1.46	1.22	2.12	2.02	2.26	1.21	1.3	1.23	0.725738339	-0.049295296	3.79E-05
117	MCOLN3	0.94	0.91	0.83	1.11	1.52	1.78	1.05	1.17	1.2	0.718545655	0.351763324	0.021923963
118	LSM7	83.21	88.76	90.44	193.59	119.23	118.48	104.63	89.15	95.17	0.716869016	0.13899717	0.013801188
119	IFFO1	0.65	0.55	0.62	1.18	0.88	0.93	0.83	0.81	0.76	0.716207034	0.399095955	0.017767416
120	RNF126P1	2.04	1.74	1.78	3.32	2.7	3.09	2.04	1.47	2.01	0.712366171	-0.010416616	0.002468004
121	SPSB1	5.71	6.07	6.21	11.08	8.98	9.3	6.86	6.11	6.81	0.706656782	0.13684724	2.01E-06
122	ADAT3	2.53	3.18	3.31	6.55	4.14	3.97	3.5	2.63	3.15	0.700685765	0.040997372	0.047483629
123	CD96	0.67	0.52	0.51	0.93	0.87	0.96	0.42	0.56	0.49	0.699133521	-0.209718591	0.004003881
124	RGCC	4.45	4.45	4.7	9.06	5.82	7.18	4.45	4.8	4.66	0.697826139	0.032515768	0.007883837
125	RAP1GAP2	5.41	5.68	5.17	8.11	9.71	8.49	6.02	5.86	5.9	0.694283991	0.128928067	5.79E-08
126	HES1	6.09	6.46	5.97	10.73	9.68	9.54	7.27	7.22	6.98	0.693471904	0.213238093	2.21E-07
127	RPS15	859.15	864.38	957.21	1949.51	1171.46	1214.23	1026.42	874.57	911.8	0.69346725	0.069370545	0.019487126
128	SLC39A3	8.56	9.01	9.54	19.61	12.25	11.94	10.1	8.64	9.17	0.692105757	0.041957011	0.019295674
129	OCIAD2	55.21	52.56	57.91	101.56	75.93	89.33	66.52	65.66	62.16	0.687467352	0.230183415	4.16E-05
130	CPNE5	1.43	1.51	1.52	2.8	2.24	2.14	2.13	1.84	1.89	0.686940134	0.393856955	0.000640071
131	NDUFS7	48.51	50.24	55.71	112.55	69.56	66.46	62.83	50.8	53.47	0.686418911	0.113478457	0.028680275
132	SUN3	2.37	1.98	2.24	3.67	3.34	3.56	3.13	2.51	2.2	0.681625006	0.250575189	0.000396019
133	HIST1H2BL	7.59	8.63	7.77	14.81	11.61	11.98	11.74	11.92	10.28	0.678673153	0.500553407	0.002025465
134	JUNB	31.44	31.26	33.88	63.81	45.09	45.12	39.66	35.67	34.9	0.673321333	0.19072055	0.001868363
135	TFR2	1.62	1.7	1.91	3.38	2.58	2.38	2.07	1.69	1.83	0.673236437	0.096037337	0.0055459
136	HHIP	1.76	1.69	1.6	2.63	2.77	2.64	1.51	1.91	1.58	0.670912114	-0.014355293	2.01E-06
137	FURIN	14.34	14.48	14.54	25.58	22.6	20.69	17.46	16.21	16.58	0.66751092	0.21275884	2.81E-07
138	GPR85	0.26	0.24	0.3	0.45	0.47	0.35	0.41	0.37	0.38	0.666756592	0.5360529	0.038915525
139	HMGN3	11.28	10.94	12.34	19.17	17.76	17.86	16.76	15.97	15.76	0.664809386	0.488584036	2.34E-08
140	ARRB2	14.65	14.98	15.81	27.82	21.66	22.34	19.45	17.25	18.01	0.660422818	0.267841721	5.40E-05
141	TRIM9	0.42	0.48	0.41	0.65	0.71	0.71	0.61	0.5	0.6	0.660063956	0.384429513	0.001471101
142	BCL2A1	16.1	15.12	16.71	27.28	21.68	26.63	16.16	16.8	18.75	0.657266449	0.109514365	6.10E-05
143	DUSP2	4.02	3.94	4.55	8.29	5.43	5.98	4.6	4.18	4.75	0.65511384	0.11308005	0.015682208
144	LOC154761	0.64	0.64	0.6	0.88	1.12	0.96	0.84	0.51	0.81	0.654864514	0.20029865	0.024593877
145	BTBD2	19.46	19.4	21.26	40.91	27.97	25.72	23.16	20.21	21.55	0.653995174	0.110817991	0.013801188
146	MFSD12	22.72	24.47	24.74	47.07	33.96	32.09	27.25	24.31	25.33	0.653188516	0.096202375	0.003718725
147	SLC25A45	0.56	0.58	0.69	0.98	1.01	0.88	0.74	0.76	0.65	0.649207088	0.232493011	0.007994589
148	DOHH	13.92	14.42	15.74	29.82	20.52	18.79	19.31	14.62	14.93	0.649187702	0.14852964	0.016017801
149	CACNA2D4	3.56	3.55	3.66	6.48	5.2	5.21	4.42	3.95	3.96	0.649151078	0.19515455	3.98E-05
150	CXCL16	1.51	1.62	1.55	2.68	2.41	2.24	1.59	1.38	1.43	0.647304669	-0.089005006	0.000653656
151	RNF126	50.19	49.2	54.76	103	71.67	66.45	59.38	49.59	54.07	0.645416433	0.080891067	0.010848219
152	CDC34	43.2	45.82	47.96	89.98	61.89	61.18	52.8	44.87	48.51	0.637226786	0.093780673	0.007975195
153	QPCT	3.57	3.62	3.77	6.15	5.7	5.16	3.63	3.87	4.35	0.634135343	0.112639261	6.55E-05
154	SPHK1	20.06	20.08	22.16	41.98	27.71	26.87	24.5	20.93	23.23	0.632193513	0.140237694	0.020847052
155	SYTL3	0.83	0.97	0.9	1.41	1.52	1.25	1.08	1.08	1.12	0.630543535	0.280736408	0.007365423
156	ZNF775	1.25	1.52	1.2	2.36	2.01	1.77	1.67	1.45	1.49	0.629099648	0.215627743	0.018405655
157	CASC8	14.55	13.93	15.95	28.66	19.59	20.28	14.38	14.05	15.24	0.625201546	-0.02489161	0.009581627
158	CDKN1A	44.06	45.22	47.11	81.45	64.72	64.13	53.66	50.78	51.13	0.624710979	0.189826008	8.07E-05
159	MBD3	57.25	60.03	65.11	122.2	81.16	77.75	69.56	58.71	63.24	0.624108144	0.070393095	0.023170856
160	EFNB3	1.14	1.3	1.18	2.05	1.86	1.65	1.42	1.13	1.36	0.619095186	0.11117891	0.002830918

161	CSNK1G2	29.43	28.87	31.03	54.9	40.5	41.66	33.62	30.38	32.31	0.617590925	0.108540841	0.001170477
162	SMAD7	1.3	1.34	1.3	2.22	2.03	1.78	1.69	1.79	1.75	0.613962372	0.408615317	0.001205722
163	PLAU	105.79	110.8	108.59	175.76	161.29	159.36	116.23	112.19	114.18	0.61029365	0.075286623	1.01E-10
164	NUMBL	10.44	10.62	11.46	20.11	15.64	13.89	10.97	11.02	10.7	0.610175858	0.00752212	0.004270247
165	NR4A1	1.89	1.95	2.07	3.23	3	2.79	2.12	2.16	2.15	0.609969303	0.121660607	3.84E-05
166	R3HDM4	32.05	30.8	32.11	58.37	43.29	43.25	36.33	34.14	33.04	0.609765316	0.124378312	0.001723877
167	GSG1	0.62	0.68	0.61	1.05	0.84	1.02	0.7	0.77	0.74	0.607446515	0.210473731	0.029722324
168	TNFSF13	1.38	1.47	1.64	2.48	2.35	1.99	1.81	1.73	1.77	0.603056294	0.241996416	0.007338021
169	POLRMT	22.3	22.82	23.88	42.75	32.59	29.43	26.05	21.95	23.95	0.602557406	0.060398325	0.004663571
170	MTRNR2L6	5.37	4.53	4.73	8.13	6	8	6.2	5.44	4.56	0.597073682	0.147064044	0.011353806
171	UQCR11	51.06	53.06	53.76	100.07	67.71	70.89	60.08	48.53	52.57	0.596188812	0.029844314	0.011388067
172	PLA2G4A	1.79	2.04	1.9	2.54	2.98	3.13	2.16	1.99	2.06	0.594164994	0.116058129	0.001640586
173	EMBP1	1.45	1.29	1.47	1.92	2.13	2.3	1.58	1.68	1.72	0.592936359	0.242325509	0.000779023
174	C9orf116	5.59	5.44	6.06	10.47	7.63	7.63	5.29	5.16	5.12	0.59029906	-0.134383453	0.02399303
175	BSG	219.18	219.08	235.52	425.52	291.87	295.01	255.03	226.4	228.08	0.587429902	0.074545413	0.013673719
176	HMG20B	27.67	31.12	32.6	57.73	39.94	39.58	34.09	30.17	31.02	0.586697932	0.060137101	0.021505329
177	HAPLN3	4.4	5.37	5.02	8.92	6.94	6.33	5.94	5.25	5.75	0.585287615	0.195811822	0.015647757
178	MEX3D	10.59	10.28	10.99	18.79	14.31	14.69	11.05	9.86	11	0.584962501	0.002262342	0.00123693
179	NEDD9	0.31	0.27	0.32	0.42	0.49	0.44	0.38	0.36	0.33	0.584962501	0.24961389	0.040434293
180	HHIP-AS1	1.81	1.76	2.1	2.77	2.61	3.12	2.78	2.12	2.13	0.584114106	0.310175954	0.025874693
181	PIGL	4.34	4.01	4.65	7.23	6.05	6.18	5.55	4.78	4.63	0.582000087	0.202598552	0.002406321
182	MT2A	1261.74	1186.94	1394.58	2483.69	1629.88	1635.22	1624.89	1471.81	1485.01	0.580927752	0.253555567	0.037076793
183	MRPS2	116.23	116.83	133.9	234.57	160.1	153.73	140.64	123.09	123.33	0.579605759	0.07693441	0.031906264
184	CEBPA	2.55	2.76	2.63	4.73	3.41	3.72	3.02	2.47	2.88	0.578893097	0.076088615	0.010050509
185	RGS7	1.87	1.56	1.39	2.26	2.49	2.44	1.62	1.68	1.97	0.576958624	0.128769815	0.010050509
186	IER2	37.91	39.11	40.55	66.29	54.72	54.26	44.08	41.9	43.96	0.5760591	0.144325632	6.55E-05
187	TM4SF1	71.91	70.33	75.67	114.22	98.73	111.18	74.51	73.16	73.84	0.572840152	0.023639426	9.85E-07
188	SERTAD4-AS1	8.44	8.92	9.52	16.56	10.6	12.82	10.6	9.37	9.76	0.572745334	0.145386325	0.041309984
189	DNAJB2	5.84	6.29	6.28	10.89	8.5	7.99	7.27	6.79	6.96	0.57263282	0.191273043	0.004292891
190	DHRS7	6.83	6.86	6.53	10.85	9.48	9.72	6.94	7.32	7.71	0.571581994	0.119751873	5.07E-05
191	SGTA	82.68	81.21	91.94	152.83	115.27	111.82	97.96	85.14	86.03	0.570510209	0.073117765	0.005434029
192	HEY1	0.79	0.79	0.81	1.1	1.06	1.38	0.81	0.96	0.84	0.566738742	0.127039189	0.046522399
193	PPAP2C	9.11	9.09	9.72	16.82	11.97	12.47	11.35	10.68	10.3	0.56344488	0.211574566	0.012923899
194	MIER2	9.95	10.62	11.18	19.26	14.26	13.36	12.04	11.11	10.99	0.562215978	0.104706466	0.015413671
195	NR1D1	4.96	4.64	5.1	8.5	7.07	6.13	5.74	5.19	5.14	0.561878888	0.128553774	0.006224803
196	NRARP	7.04	7.06	7.13	10.95	9.79	10.54	7.39	7.21	7.23	0.559136141	0.040207759	9.12E-07

Supplementary Table S1. 196 overrepresented genes in WT EXOSC9-expressing EXOSC9 KD MDA-MB-231.

Rank	gene	mock#1 (rpkm)	mock#2 (rpkm)	mock#3 (rpkm)	WT#1 (rpkm)	WT#2 (rpkm)	WT#3 (rpkm)	MUT#1 (rpkm)	MUT#2 (rpkm)	MUT#3 (rpkm)	log2FC (WT/mock)	log2FC (MUT/mock)	adjusted p value (mock vs WT)
1	ALDH1A1	0.1	0.07	0.11	0	0	0	0.1	0.11	0.12	-infinity	0.237039197	0.008104523
1	RNF43	0.05	0.05	0.03	0	0	0	0.03	0.04	0.03	-infinity	-0.378511623	0.021923963
1	ZCCHC5	0.08	0.12	0.03	0	0	0	0.04	0.04	0.09	-infinity	-0.436099115	0.002364035
1	ZSCAN23	0.08	0.03	0.07	0	0	0	0.04	0.06	0.03	-infinity	-0.469485283	0.018970747
5	KLHL29	12.85	12.74	11.47	7.03	8.67	8.54	10.83	11.23	11.09	-0.612473183	-0.160854011	0.000298912
6	CELSR2	3.34	3.29	2.65	1.7	2.38	1.98	2.52	2.35	2.51	-0.614807012	-0.330503989	0.016708655
7	ZNF669	4.88	4.98	4.27	2.55	3.16	3.51	3.92	4.42	4.4	-0.61592281	-0.149396188	0.012458474
8	NRG1	10.03	10.4	9	5.94	6.74	6.51	7.27	7.94	7.78	-0.616932831	-0.356281076	1.02E-05
9	MAMLD1	8.08	8.62	7.64	4.22	6.07	5.58	7.22	7.33	7.35	-0.61702704	-0.152398298	0.009292423
10	HTRA3	1.69	1.67	1.83	1.37	1.12	0.89	1.81	1.43	1.62	-0.618711292	-0.094778225	0.043596164
11	PIR	2.99	3.35	3.43	2.42	1.94	2	2.83	3.39	3.07	-0.619331797	-0.072679966	0.013685768
12	SLCO3A1	1.95	2.15	1.83	1.25	1.32	1.29	1.6	1.58	1.57	-0.619431257	-0.320104591	0.00021271
13	NR1H3	2.49	2.85	2.86	2.08	1.68	1.56	3.07	2.67	2.4	-0.624197664	-0.010595115	0.015855845
14	OSR1	3.92	3.87	3.91	2.84	2.23	2.52	3.47	3.12	2.87	-0.624336739	-0.306596441	0.002664848
15	GNE	8.36	8.88	7.81	4.5	5.78	5.94	7	7.4	7.35	-0.627036784	-0.203795202	0.001784259
16	ARMCX2	10.07	10.66	11.73	6.78	7.33	6.83	11.17	12.76	11.83	-0.632401558	0.139683737	5.93E-06
17	TCEAL8	8.21	8.37	8.44	5.33	5.11	5.7	9.15	9.11	9.52	-0.632441211	0.15096481	5.26E-05
18	ANKRD1	469.11	457.55	490.59	269.95	309.49	334.02	423.51	439.75	449.97	-0.633680809	-0.109974656	1.48E-05
19	TMC5	1.1	1.03	1.16	0.56	0.82	0.74	0.76	0.92	0.78	-0.634023319	-0.419429268	0.02384625
20	IL2RB	3.03	3.56	3.57	2.15	2.45	1.94	3.63	3.52	3.15	-0.635537861	0.019743935	0.001911731
21	SCIN	1.19	1.25	1.04	0.7	0.74	0.8	0.93	0.99	1.01	-0.635588574	-0.248186641	0.015047984
22	CLIP4	8.47	8.5	7.36	3.89	5.83	5.94	6.3	6.87	6.79	-0.635652108	-0.2856246	0.028695056
23	SPTLC2	11.14	11.09	9.3	5.25	7.65	7.37	8.17	9.36	9.51	-0.637379081	-0.221630018	0.016042454
24	OAS3	11.79	12.26	10.75	6.44	8.41	7.51	10.61	10.99	10.56	-0.638167118	-0.1138199	0.000436835
25	CPS1	0.62	0.67	0.61	0.35	0.4	0.47	0.56	0.54	0.58	-0.639118271	-0.177538186	0.02409331
26	ZFYVE28	1.28	1.35	1.22	0.92	0.8	0.75	1.09	1.19	1.02	-0.640347404	-0.222392421	0.001471101
27	DPF3	1.2	1.35	1.16	0.79	0.83	0.76	0.95	1	1.11	-0.640457613	-0.277887534	0.001724457
28	SUSD1	6	6.25	5.86	3.71	4.07	3.83	5.13	5.81	5.12	-0.641418574	-0.173314653	8.62E-07
29	OPN3	4.77	4.84	3.95	2.5	3.26	2.93	3.4	4.03	4.14	-0.641929096	-0.228968314	0.004686538
30	PARD6B	1.54	1.51	1.29	0.85	0.91	1.02	1.39	1.31	1.3	-0.64261016	-0.117695043	0.003904483
31	NPC1	20.73	20.52	18.55	10.64	14.91	12.73	17.88	18.34	18.14	-0.643554655	-0.137600028	0.002232276
32	LAMA5	12.47	13.31	11.94	8.53	8.34	7.26	11.49	10.7	10.75	-0.64450176	-0.195489121	3.33E-06
33	CPA4	2.81	3.09	2.87	1.68	1.7	2.23	2.86	2.52	2.74	-0.644576072	-0.111097115	0.007867639
34	CADM1	9.27	9.3	8.2	4.17	6.61	6.34	7.27	8.73	8.03	-0.644934437	-0.155780491	0.032216496
35	PPAPDC1	6.94	5.89	7.51	5.21	3.83	3.96	6.18	5.37	5.85	-0.645808056	-0.225232373	0.018405655
36	P3H2	32.28	31.6	32.04	18.88	20.41	21.92	29.25	29.21	31.25	-0.648064291	-0.096562847	2.95E-08
37	EVI2A	11.99	11.88	13.48	7.77	7.49	8.56	9.88	10.67	10.49	-0.64893483	-0.266979685	8.48E-06
38	FAM133A	1.28	1.12	1.16	0.7	0.76	0.81	1.02	1.15	1.19	-0.649184944	-0.083416008	0.008625393
39	TMEM38A	6.65	6.28	7.08	4.36	3.81	4.58	5.57	4.99	5.84	-0.65022392	-0.287025352	0.000372187
40	SHC3	2.92	2.95	2.33	1.33	2.08	1.8	2.19	2.5	2.36	-0.654340537	-0.218000652	0.030979619
41	BDNF	1.22	1.33	1.31	0.73	0.8	0.92	1.29	1.19	1.29	-0.655819098	-0.034036324	0.000680567
42	TNK1	0.85	0.91	0.84	0.55	0.55	0.55	0.87	0.69	0.81	-0.656045599	-0.133624564	0.026045804
43	BMP4	10.34	10.96	12.06	8.21	6.67	6.29	11.35	10.25	10.68	-0.656098019	-0.04747871	0.00137471
44	IFIT3	23.02	23.17	22.8	14.86	13.31	15.6	19.63	20.73	19.2	-0.656444877	-0.212043507	2.16E-07
45	MTUS1	2.06	1.98	1.79	0.95	1.51	1.23	1.45	1.86	1.75	-0.659875067	-0.204358499	0.020581834
46	HIP1	2.59	2.81	2.47	1.27	2.03	1.68	2.26	2.54	2.41	-0.660217893	-0.126364376	0.0218192
47	DISC1	0.88	0.77	0.66	0.39	0.57	0.5	0.63	0.71	0.63	-0.661924483	-0.229697222	0.031914427
48	BAHCC1	3.73	3.94	3.63	2.25	2.57	2.31	3.52	3.39	3.3	-0.664348791	-0.146339906	2.08E-07
49	ADAP2	0.95	0.89	1.1	0.64	0.64	0.57	1.06	1.09	0.98	-0.668290884	0.090346502	0.033028653
50	CASZ1	0.8	0.72	0.74	0.52	0.46	0.44	0.64	0.6	0.57	-0.670431843	-0.320333075	0.000886749
51	KCNN3	0.6	0.58	0.54	0.36	0.38	0.34	0.47	0.53	0.42	-0.671377253	-0.276517635	0.000163856
52	SH3TC1	0.71	0.88	0.8	0.51	0.58	0.41	0.63	0.55	0.6	-0.672048117	-0.425133377	0.043778122
53	IDS	28.69	28.85	26.32	13.93	19.95	18.72	23.62	26.87	25.34	-0.672920031	-0.145214107	0.002386028
54	HCAR1	1.69	1.88	1.78	0.93	1.09	1.33	1.52	1.62	1.66	-0.675377796	-0.156504486	0.011869898
55	SPEG	0.36	0.33	0.38	0.26	0.19	0.22	0.29	0.34	0.31	-0.675377796	-0.186878135	0.013783708
56	MORC4	9.35	9.32	9.31	5.05	6.06	6.41	7.33	7.49	7.86	-0.675393188	-0.302975322	2.32E-05
57	GPC1	9.39	10.1	10.81	7.63	5.9	5.41	10.36	8.82	8.86	-0.677881463	-0.111831444	0.003002709
58	LINC0070	1.91	1.63	1.87	1.13	1.06	1.19	1.56	1.71	1.69	-0.678605348	-0.125288473	0.00181528
59	TRIM61	2.64	2.86	2.77	1.51	1.73	1.92	2.44	2.07	2.65	-0.680516264	-0.207927742	0.005352545
60	GAREM	0.48	0.4	0.47	0.23	0.31	0.3	0.4	0.45	0.41	-0.684498174	-0.099535674	0.034526204
61	PCNXL2	3.24	3.15	2.61	1.43	2.18	1.98	2.53	2.83	2.75	-0.687076718	-0.150223087	0.014845298
62	MX1	6.16	6.18	5.83	3.57	4.1	3.61	5.78	5.78	5.68	-0.687791352	-0.075798645	4.35E-07
63	GPRC5A	39.55	38.02	34.14	21.5	25.55	22.18	27.75	29.38	30.59	-0.690289085	-0.348780621	3.83E-06
64	TFAP2C	7.93	8.53	7.52	4.52	5.23	5.1	7.2	7.15	6.9	-0.691368728	-0.174368818	2.77E-06
65	MEGF6	2.72	2.89	2.88	1.71	1.79	1.75	2.41	2.27	2.32	-0.693447131	-0.278409632	5.13E-09
66	IQCC	0.99	1.24	1.15	0.72	0.61	0.76	1.25	1.01	0.88	-0.693520304	-0.106258687	0.03863255
67	DENND5B	2.05	2.24	1.52	0.92	1.35	1.32	1.36	1.78	1.63	-0.69455432	-0.284548897	0.036818784
68	SPTBN5	0.37	0.45	0.41	0.26	0.27	0.23	0.43	0.39	0.37	-0.694586992	-0.047696742	0.002965771
69	ID4	1.14	0.96	1.17	0.71	0.59	0.72	0.9	0.89	0.95	-0.694935343	-0.255114743	0.009263528
70	TNFRSF2	39.28	39.45	36.55	19.46	26.66	25.03	33.75	36.51	36.81	-0.696206579	-0.106587933	0.000310951

71	RNF213	3.53	3.61	2.7	1.46	2.57	2.02	2.33	2.95	2.72	-0.701723173	-0.298658316	0.049903295
72	PRDM11	0.57	0.56	0.53	0.32	0.38	0.32	0.56	0.52	0.49	-0.702614089	-0.080418682	0.000551338
73	SIDT1	2.07	1.77	1.62	0.88	1.21	1.26	1.31	1.75	1.72	-0.704739856	-0.191890333	0.010905653
74	ZCCHC24	6.73	6.64	5.93	3.5	4.26	4.07	5.32	5.51	5.12	-0.706150774	-0.275044424	8.65E-06
75	FAM107B	16.23	15.72	15.33	7.71	10.57	10.69	13.21	13.65	14.11	-0.706670351	-0.206662143	0.000663763
76	AJUBA	29.36	29.86	28.77	16.83	18.72	18.28	26.05	26.01	27.41	-0.708929146	-0.146929227	2.33E-12
77	WNT5A	0.89	0.91	0.74	0.37	0.62	0.56	0.68	0.76	0.8	-0.712560281	-0.181329765	0.044335242
78	SNTB1	1.71	2.16	1.79	1	1.34	1.11	1.39	1.77	1.67	-0.714205691	-0.228778864	0.003904483
79	ANKEF1	3.43	3.43	3.01	1.58	2.13	2.3	2.4	2.86	3	-0.715685094	-0.256908303	0.003717286
80	CELSR1	3.05	3.42	2.55	1.55	2.24	1.7	2.46	2.62	2.39	-0.716321284	-0.27201919	0.008280919
81	PIR-FIGF	1.37	1.46	1.48	0.98	0.87	0.77	1.22	1.62	1.35	-0.718121058	-0.040737625	0.005241058
82	ERBB3	1.13	1.27	1.16	0.51	0.88	0.77	1.01	1.07	1.02	-0.720845929	-0.199609026	0.034712221
83	PYROXD2	2.21	2.12	2.29	1.46	1.37	1.18	1.85	1.93	1.92	-0.72322898	-0.215869298	0.001502991
84	ID2	2.91	2.78	3.4	2.03	1.7	1.77	3.13	3.03	2.74	-0.724848676	-0.030474958	0.004603773
85	FAM109B	2.45	2.74	3.05	1.99	1.44	1.55	3.09	2.61	2.62	-0.726498595	0.013939191	0.007255598
86	EEF1A2	20.44	20.93	22.81	16.63	11.2	10.94	21.81	18.34	18.95	-0.727183055	-0.118965659	0.0080412
87	TGFB2-OT	5.89	5.75	7.66	3.37	3.45	4.83	5.91	5.88	6.1	-0.728270893	-0.10944746	0.021585935
88	ESRP2	1.13	1.11	1.19	0.85	0.55	0.67	1.06	1.1	1.09	-0.728577809	-0.077768858	0.014281083
89	CPT1A	7.26	7.55	6.93	3.93	4.65	4.48	5.81	6.36	6.03	-0.735197043	-0.25641349	5.49E-08
90	CECR2	0.24	0.25	0.21	0.12	0.15	0.15	0.16	0.23	0.19	-0.736965594	-0.271302022	0.016042454
91	PPP1R3C	1.01	0.8	0.89	0.49	0.5	0.63	0.67	0.75	1	-0.736965594	-0.15795236	0.036704437
92	GLTSCR1L	4.36	4.56	3.75	1.72	3.01	2.87	3.27	4.01	3.75	-0.737345201	-0.199983734	0.0330059
93	CPT1C	1.28	1.32	1.45	0.97	0.74	0.71	1.47	1.69	1.36	-0.74291486	0.158400865	0.00779262
94	MITF	12.8	12.69	11.92	5.62	8.59	8.1	10.1	11.68	11.11	-0.745733452	-0.185774958	0.002367926
95	GPR137C	1.07	1.07	0.97	0.49	0.79	0.57	0.85	0.92	0.87	-0.74938931	-0.236376651	0.021665655
96	ACSS1	3.04	3.24	3.35	1.95	1.97	1.79	2.53	2.86	2.52	-0.754045052	-0.283858103	2.03E-07
97	NUP210	19.31	19.58	17.12	8.67	13.45	11.06	17.18	18.23	17.59	-0.755370542	-0.079692069	0.002041407
98	AMPH	12.64	12.41	12.8	7.07	7.61	7.69	11.58	12.23	12.15	-0.758728044	-0.073900279	3.91E-13
99	TLR2	1.28	1.28	1.42	0.79	0.83	0.73	1.11	1.2	1.06	-0.760107674	-0.240019839	0.000404281
100	MOXD1	1.86	1.95	1.95	0.91	1.07	1.42	1.63	1.76	1.6	-0.760534065	-0.207028996	0.008433798
101	SAMD4A	14.48	15.16	11.87	5.5	10.3	8.64	10.56	13.3	12.51	-0.764214647	-0.190710006	0.034175451
102	SUSD5	2.78	2.64	2.48	1.07	2	1.57	1.94	2.22	2.41	-0.767727848	-0.265959283	0.02648858
103	IFIT2	9.88	9.65	8.23	4.31	5.75	6.22	7.11	8.12	7.28	-0.769906868	-0.302441511	0.001034871
104	TNS3	13.02	13.53	10.99	5.24	9.37	7.4	9.99	10.71	10.06	-0.770269505	-0.287373147	0.017619392
105	MYBL1	8.72	8.33	7.29	3.06	5.91	5.25	5.66	7.5	7.44	-0.775407703	-0.240684831	0.037076793
106	PXDN	46.64	48.33	39.75	19.02	32.87	26.79	38.16	42.19	39.38	-0.775895181	-0.170179359	0.010344345
107	SOGA1	6.93	7.32	5.37	2.6	4.95	3.89	4.96	5.66	5.32	-0.778237989	-0.299673412	0.040045182
108	CACNB4	1.08	1.04	0.91	0.39	0.76	0.61	0.65	0.89	0.92	-0.783742365	-0.300659478	0.037487186
109	PNMA2	3.99	3.97	3.45	1.63	2.62	2.34	2.77	3.05	3.28	-0.791948421	-0.326360341	0.003831472
110	PER3	0.84	0.7	0.59	0.39	0.44	0.4	0.59	0.6	0.62	-0.792195115	-0.234863733	0.004135451
111	SYTL2	1.96	2.2	2.11	1.04	1.28	1.3	1.88	1.98	1.91	-0.792475746	-0.119894124	1.82E-05
112	SYNE1	0.56	0.54	0.39	0.22	0.34	0.3	0.35	0.48	0.4	-0.792903766	-0.276654015	0.010050509
113	SLC12A7	2.86	3.09	2.88	1.85	1.8	1.44	2.94	2.78	2.61	-0.794747782	-0.084096942	6.72E-06
114	PDE7B	0.98	1	0.85	0.52	0.54	0.57	0.65	0.87	0.87	-0.795930089	-0.243791435	0.000175603
115	TIMP3	16.41	15.42	15.47	9.25	9.09	8.89	12.86	13.48	12.78	-0.796643201	-0.273933813	2.12E-17
116	SCN5A	2.54	2.66	2.37	1.5	1.48	1.37	2.34	2.31	2.26	-0.799277899	-0.13160759	7.71E-10
117	CRABP2	16.73	17.48	18.33	13.19	8.51	8.47	22.39	18.07	16.91	-0.800301504	0.126880318	0.00492919
118	CDC42BPA	8.57	8.7	7.08	3.11	5.83	5.01	5.94	7.31	6.85	-0.80365665	-0.276726271	0.021821617
119	CCBE1	4.97	4.78	4.73	2.32	2.95	3.02	3.82	4.53	4.26	-0.804617596	-0.199493327	2.40E-06
120	NTN4	14.91	14.17	14.48	6.6	8.96	9.36	10.51	11.59	12.07	-0.805699886	-0.350273706	6.62E-05
121	APOL6	1.79	1.71	1.4	0.69	1.19	0.92	1.17	1.44	1.39	-0.807354922	-0.292781749	0.011869898
122	STXBP2	3.27	3.63	4.25	2.56	1.99	1.82	5.17	4.02	4.18	-0.807678433	0.261955755	0.002853259
123	PLEKHA2	2.47	2.37	1.99	1.03	1.53	1.34	1.66	2	1.88	-0.808411455	-0.301999602	0.001302201
124	FAM174B	2.68	2.56	2.73	1.38	1.55	1.61	1.93	1.93	2.27	-0.811887427	-0.37869265	4.46E-06
125	JDP2	0.47	0.57	0.54	0.37	0.27	0.26	0.31	0.46	0.37	-0.811927652	-0.470890734	0.030176625
126	PCSK5	0.45	0.46	0.41	0.19	0.3	0.26	0.32	0.39	0.41	-0.815575429	-0.237039197	0.003144886
127	KCNIP3	3.77	3.92	3.77	2.95	1.86	1.69	3.84	2.99	3.13	-0.818095421	-0.202389397	0.013673719
128	ANXA8L1	1.6	1.79	1.92	1.07	1	0.94	1.31	1.4	1.7	-0.818948374	-0.267933205	0.00057818
129	DAPK1	1.16	1.23	1.07	0.53	0.71	0.72	1.25	1.45	1.35	-0.819918384	0.22714987	0.000291904
130	RAB27B	1.58	1.62	1.38	0.62	1.04	0.93	1.06	1.43	1.19	-0.8223955	-0.315641832	0.006248113
131	TNS1	1.73	1.89	1.77	1.02	1.08	0.94	1.72	1.79	1.72	-0.826213949	-0.043474327	8.97E-10
132	HES2	0.41	0.45	0.47	0.26	0.23	0.26	0.42	0.31	0.33	-0.826463745	-0.327361981	0.014088308
133	DDX60	1.3	1.27	1	0.53	0.77	0.71	0.87	0.99	1	-0.828728573	-0.319908927	0.002996398
134	EREG	8.21	8.53	7.47	3.05	5.23	5.35	6.17	7.49	7.27	-0.828817517	-0.210030768	0.010050509
135	CFH	1.19	1.08	1.25	0.51	0.72	0.75	0.93	1.13	1.1	-0.830074999	-0.15565087	0.002853259
136	LOC10050	0.91	1.19	1.11	0.57	0.67	0.56	0.74	0.68	0.78	-0.834576391	-0.545069774	0.033367169
137	DLX3	1.68	1.53	1.73	0.94	0.99	0.84	1.68	1.44	1.42	-0.834625066	-0.121818744	0.000177752
138	RASD1	1.45	1.56	2.04	1.19	0.86	0.78	1.59	1.22	1.38	-0.835481335	-0.269333144	0.031461214
139	SALL2	1.88	1.65	1.56	0.76	1.07	1.02	1.9	1.67	1.77	-0.836703737	0.069174086	0.000436835
140	DIP2C	1.76	1.91	1.51	0.73	1.15	1.02	1.25	1.53	1.44	-0.836899198	-0.295709099	0.002561344
141	PCNX	7.36	7.5	5.15	2.41	4.93	3.85	4.45	6.01	5.49	-0.838511131	-0.327164743	0.046614469
142	TBC1D12	3.93	3.85	3.25	1.59	2.31	2.26	2.9	3.16	3.06	-0.840430535	-0.274327061	0.000534788
143	ZNF813	0.68	0.63	0.59	0.26	0.48	0.32	0.48	0.68	0.63	-0.841935154	-0.086039831	0.042971585

144	C4BPB	2.61	2.87	2.9	1.81	1.15	1.7	3.01	2.57	2.41	-0.846620289	-0.068754741	0.008454808
145	COL8A1	10.25	10.43	9.04	4.14	6.83	5.55	9.01	9.52	8.7	-0.847220429	-0.126237133	0.001471101
146	LAMA3	2.36	2.4	1.83	0.9	1.53	1.23	1.78	1.97	1.82	-0.848434817	-0.242601138	0.00651738
147	PLCE1	3.48	3.41	2.84	1.29	2.29	1.82	2.26	2.73	2.47	-0.849480398	-0.383264175	0.005780552
148	NALCN	2.81	2.69	2.33	1.04	1.77	1.51	1.75	2.14	1.95	-0.857980995	-0.423043938	0.003002709
149	FGF13	3.82	4.07	4.11	2.01	2.33	2.28	3.05	2.87	3.07	-0.858131284	-0.416641385	3.82E-09
150	MAN1A1	1.63	1.61	1.37	0.53	0.98	1.03	1.23	1.51	1.45	-0.859938254	-0.137816507	0.02873461
151	ADRBK2	0.77	0.81	0.71	0.39	0.46	0.41	0.65	0.75	0.84	-0.861923865	-0.031848866	3.53E-06
152	ELOVL7	1.58	1.7	1.36	0.65	0.88	1.01	1.41	1.75	1.73	-0.869296308	0.07570966	0.002232586
153	ZSCAN18	2.24	2.33	2.43	1.52	1.19	1.12	2.8	2.38	2.54	-0.87001053	0.141245925	7.01E-05
154	TRERF1	8.03	7.88	6.56	2.96	5.12	4.17	6	6.75	6.35	-0.875218375	-0.234427486	0.002747545
155	MBP	10.23	10.28	9.12	3.88	6.38	5.89	7.18	8.53	8.31	-0.875524461	-0.302822475	0.001074721
156	APH1B	0.86	0.8	0.8	0.33	0.47	0.54	0.66	0.79	0.75	-0.876425315	-0.161154792	0.004932428
157	CSPG4	2.17	2.4	1.96	1.28	1.2	1.07	2.15	2.05	1.99	-0.879263967	-0.077143582	1.50E-07
158	ZNF594	0.8	0.88	0.73	0.3	0.43	0.58	0.8	0.84	0.88	-0.879466335	0.064390587	0.02384625
159	RAB9B	1.38	1.35	1.26	0.71	0.62	0.83	0.99	1.31	1.27	-0.885357434	-0.160464672	0.00010372
160	ISM1	1.84	1.61	1.45	0.72	0.86	1.07	1.5	1.5	1.48	-0.88678939	-0.129283017	0.002395981
161	PARD3B	0.97	0.84	0.89	0.32	0.67	0.47	0.65	0.83	0.77	-0.886991038	-0.263034406	0.025627712
162	ARID5B	1.31	1.23	1.09	0.63	0.71	0.62	1.04	1.15	1.05	-0.889115894	-0.163975735	3.34E-07
163	LYST	0.71	0.73	0.58	0.25	0.46	0.38	0.45	0.53	0.48	-0.890027158	-0.468386924	0.009581627
164	NDRG2	2	2.15	2.06	1.41	0.91	1.03	2.19	2.03	2.11	-0.890432173	0.027612231	0.000880982
165	FRAS1	1.8	1.91	1.29	0.55	1.18	0.96	1.12	1.41	1.24	-0.894321922	-0.407363571	0.039868013
166	HIST2H2B	3.26	3.49	3.2	1.55	1.94	1.86	2.67	2.4	2.31	-0.895157634	-0.431075709	0.010905653
167	RDM1	1.23	1.17	1.1	0.84	0.57	0.47	0.91	1.07	1.1	-0.89662226	-0.184424571	0.046224293
168	KANSL1L	0.48	0.56	0.46	0.16	0.3	0.34	0.43	0.55	0.41	-0.906890596	-0.109877618	0.04608645
169	TMEM229	1.06	0.94	0.96	0.65	0.47	0.45	0.82	0.85	0.88	-0.914832617	-0.215099929	0.001068721
170	ADM	17.56	17.22	18.96	10.94	8.52	9.04	23.77	19.38	20.91	-0.915034402	0.253427477	2.84E-08
171	APOBEC3	1.35	1.79	1.35	0.84	0.79	0.75	1.47	1.15	1.11	-0.915753871	-0.267539814	0.002896664
172	SERINC2	2.62	2.74	3.18	1.9	1.32	1.3	3.41	3.05	2.89	-0.917913297	0.130730295	0.00042979
173	CHST4	1.14	1.38	1.25	0.76	0.69	0.54	0.88	0.98	0.9	-0.921796093	-0.449896256	0.001911731
174	STYK1	1.31	1.33	1.13	0.55	0.71	0.73	1.13	1.18	1.39	-0.921796093	-0.027039253	0.000368742
175	MYOF	104.34	103.18	87.54	36.63	64.28	54.58	70.78	84.21	78.34	-0.924186555	-0.338636543	0.001300566
176	GSTM1	10.06	10.43	10.71	6.67	4.74	5.01	11.32	9.72	9.74	-0.926091902	-0.019552797	9.88E-06
177	GPR1	0.82	0.79	1.02	0.46	0.46	0.46	0.65	0.59	0.8	-0.930394533	-0.366493647	0.002948985
178	RAB37	0.61	0.61	0.44	0.38	0.28	0.21	0.56	0.39	0.43	-0.932095935	-0.266514975	0.036987866
179	ARNT2	4.31	4.37	3.9	1.69	2.71	2.19	3.55	3.64	3.54	-0.932781552	-0.229481846	0.000101879
180	RELN	4.65	4.69	3.6	1.47	2.77	2.52	3.31	3.92	4.1	-0.936742466	-0.191689756	0.007200677
181	RUNX2	5.53	5.29	4.89	1.8	3.28	3.11	4.23	5.06	4.47	-0.939747824	-0.191202711	0.002960291
182	PAG1	2.13	2.05	1.53	0.57	1.32	1.08	1.13	1.72	1.5	-0.943027815	-0.392475345	0.037012215
183	LOC10049	0.47	0.45	0.37	0.17	0.24	0.26	0.42	0.41	0.39	-0.945138065	-0.080489918	0.006207759
184	TGFBR3	4.93	5.23	4.04	1.49	3.3	2.58	3.2	4.21	4.18	-0.946154405	-0.293010363	0.019777106
185	AIM1	5.91	5.59	4.45	1.77	3.38	3.12	3.73	4.52	4.67	-0.947597189	-0.303950354	0.008146389
186	C10orf35	1.42	2.03	1.22	0.95	0.82	0.65	1.53	1.69	1.49	-0.948415502	0.01230451	0.046702478
187	FAM20C	6.84	7.77	7.85	4.73	3.57	3.32	6.42	5.99	5.76	-0.950747859	-0.305799508	8.73E-06
188	COLGALT	2.07	1.91	1.72	0.86	1.08	1	1.4	1.4	1.37	-0.955145764	-0.450914536	1.45E-07
189	CREB5	2.07	2.28	1.84	0.73	1.27	1.19	1.49	1.88	1.83	-0.956382985	-0.251427786	0.001784259
190	NMNAT2	2.48	2.41	1.98	1.08	1.37	1.09	2.21	2.08	2.01	-0.956560739	-0.12495827	2.14E-06
191	NEK11	0.76	0.74	0.83	0.36	0.39	0.45	0.7	0.81	0.86	-0.957295549	0.024557104	0.000787209
192	PAOX	1.53	1.5	1.33	1.07	0.64	0.52	1.04	1.16	1.38	-0.967284425	-0.284368548	0.027308833
193	C4orf19	1.11	1.1	0.88	0.51	0.51	0.56	0.96	0.96	1.01	-0.96768228	-0.076706173	6.89E-05
194	GPR64	0.71	0.64	0.57	0.26	0.35	0.37	0.5	0.57	0.55	-0.970252657	-0.245112498	0.001204394
195	FAT1	21.64	22.03	14.13	5.86	13.36	10.26	13.25	16.64	15.1	-0.971332968	-0.361465126	0.033653289
196	ARMCX4	0.41	0.37	0.32	0.15	0.23	0.18	0.38	0.45	0.43	-0.974004791	0.19592021	0.005842095
197	GPC4	2.67	2.59	2.62	1.14	1.59	1.28	2.73	3	2.91	-0.974593393	0.132835683	4.92E-07
198	SATB1	0.82	0.85	0.67	0.28	0.48	0.43	0.61	0.83	0.61	-0.975546956	-0.19088462	0.003124523
199	GPR37	0.42	0.44	0.44	0.24	0.19	0.23	0.42	0.53	0.32	-0.977973694	-0.033683126	0.005780552
200	DENND2A	2.5	2.51	2.55	1.27	1.26	1.29	2.24	1.77	1.83	-0.984813596	-0.372417865	1.94E-11
201	C8orf46	0.63	0.64	0.67	0.32	0.32	0.34	0.4	0.53	0.57	-0.985202998	-0.371094152	0.000492473
202	HFE	1.96	2.02	1.53	0.63	1.13	1.02	1.62	1.5	1.56	-0.986967436	-0.235543789	0.005723495
203	AHNAK	28.02	30.32	17.86	7.08	17.46	13.59	17.24	21.98	20.18	-0.998864467	-0.359328067	0.047888028
204	FSIP2	0.14	0.11	0.09	0.04	0.06	0.07	0.09	0.11	0.08	-1	-0.280107919	0.016248543
205	PTPRS	6.61	6.47	5.9	2.98	3.34	3.17	5.38	5.46	5.28	-1	-0.235628248	3.97E-17
206	SAA2	0.51	0.57	0.64	0.27	0.26	0.33	0.55	0.55	0.56	-1	-0.051225323	0.016459946
207	NTNG2	0.68	0.61	0.62	0.38	0.26	0.31	0.59	0.55	0.51	-1.00757322	-0.211106614	0.002876875
208	GPR116	16.76	17.01	14.04	4.87	10.16	8.71	12.11	15.03	14.54	-1.009992471	-0.197957128	0.005014023
209	ZNF114	2.38	2.28	2.71	1.27	1.27	1.1	2.16	2.39	2.62	-1.017726169	-0.0396915	1.06E-07
210	MST1R	0.68	0.76	0.85	0.36	0.4	0.37	0.72	0.66	0.62	-1.019024826	-0.195347598	1.66E-05
211	DYNC1H1	77.31	81.15	56.31	26.82	44.46	34.53	52.09	59.13	56.81	-1.021316504	-0.354073651	0.000481181
212	ILDR2	1.1	1.13	1	0.37	0.66	0.55	0.78	0.92	0.88	-1.031609607	-0.324163099	0.00035164
213	CMPK2	0.52	0.59	0.59	0.27	0.29	0.27	0.47	0.46	0.39	-1.034351505	-0.364996817	0.000352633
214	ABI3BP	1.2	1.34	1.25	0.46	0.6	0.79	1.06	1.31	1.37	-1.034672578	-0.019159578	0.00038639
215	L1CAM	0.66	0.79	0.59	0.38	0.33	0.28	0.62	0.45	0.53	-1.043068722	-0.350497247	0.000310951
216	ANK1	2.19	2.32	2.03	0.91	1.22	1.04	1.7	1.81	1.67	-1.044807795	-0.336338538	2.43E-09

217	PTPRJ	8.48	8.67	6.91	3	4.7	3.96	5.36	6.43	5.96	-1.045068854	-0.438817618	1.21E-05
218	UTRN	5.19	5.67	3.64	1.24	3.23	2.53	2.95	4.2	3.76	-1.050626073	-0.410401799	0.033723155
219	VAMP8	19.54	20.3	23.28	12.5	9.14	8.82	24.85	22.13	22.47	-1.051181264	0.137877489	1.57E-06
220	CRIM1	92.66	91.81	73.92	22.69	55.64	46.21	60.11	74.32	72.74	-1.052941053	-0.318735134	0.014633364
221	OBSCN	0.25	0.28	0.2	0.12	0.13	0.1	0.22	0.2	0.2	-1.060541542	-0.235628248	3.42E-06
222	MYLK	3.54	3.64	2.89	1.15	2.02	1.62	2.64	2.87	2.66	-1.071966122	-0.3016557	0.000151118
223	PTPRM	7.48	7.7	6.37	2.48	4.03	3.73	5.69	6.47	6.03	-1.073472154	-0.244542326	2.26E-05
224	LHFPL4	0.58	0.69	0.74	0.36	0.27	0.32	0.72	0.68	0.62	-1.081196083	0.007159792	8.02E-05
225	CADPS2	0.6	0.7	0.5	0.23	0.29	0.33	0.56	0.61	0.65	-1.08246216	0.015941544	0.000290473
226	FHDC1	0.19	0.18	0.16	0.07	0.09	0.09	0.11	0.16	0.11	-1.084064265	-0.479992941	0.024974271
227	APBA1	0.21	0.22	0.23	0.08	0.14	0.09	0.15	0.16	0.17	-1.090197809	-0.459431619	0.014845298
228	PCDH7	0.2	0.21	0.21	0.07	0.1	0.12	0.22	0.18	0.22	-1.096215315	0	0.00303984
229	HEG1	17.02	17.92	13.06	4.45	9.93	8.03	10.73	13.81	12.96	-1.098891757	-0.35614381	0.004906209
230	FAM49A	2.09	2.22	2.04	0.91	1.13	0.92	1.74	1.99	1.73	-1.101159416	-0.217855641	7.74E-11
231	SAA1	2.54	2.46	1.83	1.21	0.97	1	2.4	2.77	2.52	-1.102858813	0.17109802	0.005014023
232	EPAS1	5.18	5.45	4.81	1.95	2.9	2.32	4.21	4.91	4.68	-1.106627729	-0.162004486	4.60E-08
233	KAZN	0.94	1.07	1.17	0.48	0.54	0.45	0.86	1.05	0.95	-1.11321061	-0.153011619	1.83E-09
234	SUCLG2-A	2.21	2.28	1.87	0.75	1.07	1.12	1.49	1.55	1.54	-1.11321061	-0.473679167	1.37E-05
235	ATP10A	2.02	2.07	1.78	0.86	0.96	0.89	1.39	1.29	1.38	-1.115067652	-0.531880776	1.81E-12
236	VSTM2L	0.93	1.06	1	0.52	0.39	0.47	1.13	0.95	1.2	-1.115477217	0.13355033	0.000544343
237	CXCR4	4.87	4.68	5.4	2.61	2.09	2.16	3.99	3.88	4.63	-1.123865003	-0.25821739	2.98E-10
238	IL11	40.15	41.73	44.35	23.33	17.01	17.58	45.17	40.31	42.04	-1.123921316	0.01466871	6.15E-10
239	LHPP	1.05	1.46	1.41	0.71	0.44	0.64	1.24	0.93	1.06	-1.130894067	-0.279319489	0.00321219
240	IL7R	9.1	9.02	8.5	3.31	4.38	4.46	7.75	8.4	9.03	-1.131554257	-0.080232288	2.79E-11
241	CPE	3.65	3.74	3.97	1.62	1.66	1.89	3.64	3.43	3.41	-1.135726649	-0.116324118	1.27E-12
242	C3	4.54	4.98	4.35	1.98	2.3	1.89	5.78	6.03	5.14	-1.168625393	0.289317486	1.91E-15
243	GSTM2	2.92	2.85	3.09	1.53	1.21	1.2	2.84	2.76	2.67	-1.169111069	-0.099419369	3.46E-09
244	KCTD4	0.55	0.36	0.62	0.26	0.19	0.23	0.58	0.54	0.61	-1.169925001	0.177240385	0.032580656
245	SORL1	0.17	0.15	0.11	0.04	0.07	0.08	0.16	0.17	0.15	-1.178337241	0.158697746	0.011648345
246	CYTL1	0.98	1.35	1.32	0.48	0.6	0.53	1.18	1.24	1.4	-1.180835776	0.065676174	0.007579516
247	ARRDC4	0.89	0.97	0.93	0.26	0.41	0.56	0.79	0.75	0.81	-1.181606806	-0.247604365	0.001808953
248	NXF5	1.02	1.12	0.81	0.53	0.32	0.45	1.2	0.96	0.92	-1.182203331	0.062215396	0.00409563
249	SERPINB9	2.14	2.11	1.88	0.73	1.07	0.9	1.53	1.83	1.8	-1.182927667	-0.248516008	2.84E-08
250	FBP1	9.37	9.38	10.01	5.18	3.44	4.03	8.14	7.46	8.22	-1.184926291	-0.271890263	1.12E-08
251	OLFML2A	0.92	0.9	0.91	0.46	0.34	0.4	0.61	0.65	0.64	-1.185866545	-0.522901533	1.31E-09
252	DNAH2	1.65	1.7	1.35	0.53	0.84	0.69	1.1	1.2	1.05	-1.190016419	-0.488499661	7.68E-07
253	HLA-DMA	2.29	2.28	2.26	1.18	1	0.81	2.48	2.15	2.21	-1.191740094	0.002110747	2.41E-05
254	FAT4	0.64	0.73	0.42	0.12	0.37	0.29	0.35	0.47	0.41	-1.198413558	-0.541301272	0.036749206
255	EVI2B	21.79	19.24	22.48	9.12	8.57	9.96	17.26	18.4	16.91	-1.19970429	-0.272744035	1.39E-18
256	CRISPLD2	4.51	4.24	3.75	1.47	2.16	1.81	2.93	3.45	3.11	-1.200249538	-0.397448103	4.63E-09
257	PITPNM3	0.74	0.81	0.69	0.35	0.37	0.25	0.62	0.65	0.61	-1.20744208	-0.25276607	7.01E-07
258	CDH11	39.33	40.17	36.87	13.46	18.87	17.41	36.27	39.01	38.24	-1.226240769	-0.035772687	1.16E-12
259	CCDC180	0.32	0.33	0.31	0.1	0.17	0.14	0.36	0.31	0.29	-1.227410496	0	0.001085611
260	COL4A5	0.22	0.23	0.16	0.08	0.08	0.1	0.19	0.23	0.21	-1.230297619	0.046542586	0.004292891
261	NPR3	0.27	0.23	0.26	0.11	0.11	0.1	0.27	0.27	0.26	-1.247927513	0.074000581	8.42E-05
262	NEURL1	0.4	0.18	0.3	0.15	0.12	0.1	0.23	0.22	0.21	-1.249978253	-0.415037499	0.041308531
263	MUC15	0.44	0.3	0.31	0.14	0.12	0.18	0.35	0.39	0.43	-1.254813899	0.156119202	0.007878913
264	SP140	4.08	3.64	3.86	1.42	1.69	1.74	3.28	3.12	3.3	-1.255578601	-0.255578601	8.79E-16
265	DST	9.72	10.15	6.8	2.32	4.72	4.12	5.63	7.52	6.73	-1.256880798	-0.423900068	0.000436835
266	BMPER	2.45	2.35	2.37	0.82	1.18	0.99	2.09	2.07	2.23	-1.261827635	-0.166157188	1.65E-11
267	SELENBP	0.77	0.55	0.84	0.28	0.33	0.29	1.02	0.94	0.72	-1.263034406	0.311201688	0.002596456
268	MATN2	0.76	0.67	0.61	0.21	0.33	0.3	0.56	0.44	0.58	-1.280107919	-0.368644594	1.85E-05
269	FCGBP	0.12	0.18	0.12	0.06	0.07	0.04	0.11	0.12	0.08	-1.304854582	-0.438121112	0.003124523
270	AMIGO1	1.16	0.94	0.97	0.29	0.5	0.44	0.87	0.67	0.83	-1.31958034	-0.373351596	6.13E-05
271	KIF6	0.21	0.2	0.24	0.1	0.12	0.04	0.29	0.23	0.21	-1.321928095	0.167456746	0.032632116
272	TNFRSF1B	0.99	1.06	1.12	0.45	0.36	0.45	1.16	1.03	1.16	-1.331059107	0.079678255	8.68E-09
273	B3GALT5	0.81	0.92	0.72	0.24	0.38	0.35	0.33	0.71	0.61	-1.336725097	-0.570315725	3.66E-05
274	SLFN13	0.47	0.38	0.42	0.15	0.17	0.18	0.31	0.34	0.37	-1.344828497	-0.316259345	2.07E-08
275	KLHL4	2.4	2.27	2.19	0.63	1.06	1.01	1.45	1.76	1.69	-1.345249169	-0.485426827	6.12E-08
276	CNIH3	1.47	1.67	1.18	0.62	0.59	0.49	1.16	1.17	1.21	-1.345496566	-0.287281952	1.42E-06
277	ACOT4	0.37	0.57	0.41	0.19	0.18	0.16	0.26	0.37	0.28	-1.348895142	-0.569020957	0.030499796
278	CTSW	0.6	0.66	0.87	0.4	0.3	0.13	0.51	0.6	0.44	-1.359670189	-0.458585215	0.043708
279	USP44	0.46	0.41	0.4	0.14	0.18	0.17	0.43	0.36	0.31	-1.373974843	-0.207324973	3.00E-05
280	ZDHHC22	0.49	0.47	0.37	0.21	0.22	0.08	0.51	0.46	0.5	-1.382857094	0.144389909	0.008876575
281	SLC52A3	0.74	0.5	0.67	0.21	0.27	0.25	0.66	0.47	0.53	-1.387604269	-0.202389397	0.000231483
282	SEMA3B	1.77	2.01	2.22	0.96	0.64	0.67	1.63	1.53	1.48	-1.402270203	-0.370837695	1.63E-08
283	SLC16A2	1.64	1.84	1.8	0.66	0.67	0.66	1.5	1.34	1.31	-1.407769499	-0.347426593	1.81E-17
284	CFB	0.49	0.57	0.54	0.16	0.2	0.24	0.41	0.43	0.33	-1.415037499	-0.451563375	0.000222482
285	FAM65C	0.7	0.92	0.78	0.32	0.31	0.26	0.89	0.69	0.78	-1.431157165	-0.024247546	1.10E-08
286	SLAMF7	3.92	3.61	4.06	1.28	1.44	1.56	3.53	3.47	3.41	-1.437197865	-0.154910498	8.35E-20
287	ARHGEF6	0.14	0.19	0.19	0.06	0.07	0.06	0.15	0.15	0.14	-1.452512205	-0.2410081	0.003109694
288	LTB	1.64	1.46	1.76	0.86	0.61	0.3	1.74	1.48	1.15	-1.457206954	-0.153323034	0.004932428
289	ATP8A1	0.36	0.35	0.23	0.05	0.15	0.14	0.23	0.29	0.26	-1.46712601	-0.269186633	0.010050509

290	LOXL4	1.34	1.38	1.31	0.58	0.45	0.41	0.94	0.83	0.97	-1.484711027	-0.556603946	9.76E-12
291	RIMBP2	0.89	1.1	1.04	0.37	0.35	0.36	0.7	0.68	0.7	-1.488286481	-0.542734265	9.87E-15
292	SAA2-SAA	0.52	0.64	1.09	0.18	0.25	0.35	0.6	0.64	0.58	-1.528378972	-0.305986551	0.049818246
293	ZBTB7C	0.18	0.19	0.24	0.04	0.11	0.06	0.16	0.16	0.17	-1.538419915	-0.316027493	0.010657676
294	WT1	0.33	0.32	0.43	0.19	0.09	0.09	0.32	0.17	0.26	-1.545434137	-0.526068812	0.004645583
295	SCG2	0.32	0.34	0.34	0.12	0.14	0.08	0.38	0.3	0.3	-1.556393349	-0.029146346	0.002828283
296	THRB	0.48	0.52	0.45	0.13	0.17	0.19	0.32	0.42	0.39	-1.565199246	-0.359730128	4.79E-10
297	IL1RL2	0.7	0.82	0.77	0.2	0.25	0.32	0.8	0.57	0.74	-1.572417247	-0.118104599	3.27E-05
298	COL7A1	4.55	5.36	4.61	1.56	1.8	1.49	4.92	4.91	4.27	-1.581984801	-0.042346291	4.43E-28
299	KCNE1	0.31	0.33	0.35	0.1	0.1	0.13	0.26	0.33	0.27	-1.584962501	-0.203091865	4.16E-05
300	VAT1L	2.53	2.33	2.07	0.75	0.78	0.78	1.59	1.77	1.92	-1.584962501	-0.392317423	2.28E-20
301	BZRAP1	0.1	0.08	0.09	0.02	0.03	0.04	0.14	0.09	0.1	-1.584962501	0.289506617	0.010959844
302	SLC4A4	0.65	0.53	0.45	0.13	0.18	0.23	0.39	0.43	0.46	-1.593840652	-0.348728154	2.53E-07
303	PBX1	0.43	0.41	0.3	0.11	0.15	0.11	0.28	0.32	0.27	-1.623436649	-0.389946518	2.53E-07
304	SERPINF1	0.39	0.33	0.33	0.17	0.11	0.06	0.34	0.26	0.32	-1.626782676	-0.190683562	0.032646911
305	ANO2	0.26	0.19	0.18	0.07	0.07	0.06	0.16	0.18	0.17	-1.655351829	-0.304854582	0.002443006
306	CIITA	0.57	0.7	0.57	0.19	0.21	0.18	0.48	0.46	0.52	-1.665580961	-0.333737397	4.92E-10
307	GPR133	0.15	0.28	0.22	0.07	0.05	0.08	0.23	0.22	0.26	-1.700439718	0.127379306	0.001724457
308	SHANK2	0.42	0.41	0.44	0.09	0.16	0.14	0.36	0.48	0.43	-1.703282468	0	2.57E-10
309	HAS2	2.72	2.84	2.39	0.57	0.96	0.9	2.06	2.39	2.15	-1.709998547	-0.268488836	6.22E-12
310	GRIK4	0.39	0.39	0.41	0.07	0.16	0.12	0.25	0.29	0.3	-1.765534746	-0.502500341	2.90E-07
311	SPTLC3	2.54	2.57	2.74	0.54	0.84	0.9	1.76	2.19	2.07	-1.78365883	-0.382929167	6.08E-15
312	CACNA1C	0.06	0.06	0.02	0.02	0.01	0.01	0.05	0.04	0.03	-1.807354922	-0.222392421	0.039054146
313	IL24	6.8	6.85	7.25	2.21	1.77	1.97	5.57	4.41	5.04	-1.812541369	-0.476618129	1.07E-33
314	LINC0144	2.17	2.05	1.9	0.68	0.38	0.66	2.04	1.67	1.28	-1.831123088	-0.294491837	2.01E-06
315	PTGS1	0.2	0.31	0.28	0.08	0.08	0.06	0.23	0.23	0.27	-1.84434913	-0.113956189	1.38E-05
316	DAAM2	1.9	1.9	1.76	0.57	0.52	0.44	1.8	1.85	1.72	-1.86155323	-0.050162795	3.34E-30
317	RCSD1	2.84	2.91	2.47	0.72	0.75	0.77	2.01	2.16	1.97	-1.875639662	-0.420899738	2.68E-27
318	FAT3	0.63	0.52	0.51	0.09	0.17	0.19	0.4	0.48	0.43	-1.883186335	-0.34161643	2.07E-10
319	MAL2	2.83	2.34	2.32	0.56	0.74	0.7	1.85	2.29	1.98	-1.904965719	-0.291434066	4.71E-20
320	DIO2	0.34	0.45	0.43	0.09	0.11	0.12	0.36	0.39	0.34	-1.930737338	-0.162553013	9.35E-11
321	MGAT5B	2.86	2.92	3.08	0.79	0.88	0.63	3	2.3	2.55	-1.945672838	-0.174614045	1.01E-29
322	ZNF415	0.3	0.19	0.18	0.02	0.09	0.06	0.16	0.28	0.27	-1.978626349	0.083657929	0.013801188
323	TP53I11	0.45	0.7	0.51	0.15	0.2	0.07	0.4	0.44	0.43	-1.982722009	-0.386354745	2.26E-05
324	HECW1	0.29	0.34	0.23	0.03	0.09	0.09	0.22	0.25	0.23	-2.033947332	-0.296981738	6.38E-06
325	HLA-DMB	1.17	1.04	1.13	0.27	0.22	0.3	0.94	0.82	1.03	-2.079923544	-0.259582981	1.19E-08
326	SMPD3	0.32	0.32	0.29	0.07	0.07	0.07	0.26	0.2	0.21	-2.146841388	-0.473069621	1.69E-09
327	CYB5R2	1.05	1.08	0.98	0.29	0.23	0.18	1.12	1.03	0.94	-2.151487753	-0.009307742	8.14E-11
328	LOC10050	0.27	0.3	0.28	0.03	0.09	0.07	0.23	0.24	0.17	-2.161463423	-0.409390936	5.69E-06
329	MARVELD	0.45	0.61	0.33	0	0.12	0.17	0.37	0.4	0.43	-2.260960078	-0.212050477	0.047549771
330	PI3	0.68	0.34	0.68	0.16	0.07	0.1	0.84	0.57	0.57	-2.364996817	0.219965684	0.035348369
331	LINC0090	0.19	0.24	0.21	0.02	0.09	0.01	0.24	0.18	0.17	-2.415037499	-0.117356951	0.016777885
332	AR	0.41	0.48	0.31	0.07	0.07	0.08	0.3	0.36	0.25	-2.447458977	-0.399095955	5.37E-18
333	SLCO4C1	0.13	0.09	0.06	0	0.03	0.02	0.07	0.07	0.12	-2.485426827	-0.106915204	0.043088392
334	PYCARD	0.73	0.94	0.63	0.16	0.16	0.02	0.8	0.49	0.55	-2.75802721	-0.321928095	0.000663763
335	GCNT4	0.11	0.13	0.11	0	0.03	0.02	0.09	0.11	0.12	-2.807354922	-0.129283017	0.019989364
336	HLA-DPB	0.22	0.2	0.18	0.02	0.01	0.05	0.2	0.14	0.17	-2.906890596	-0.234465254	6.34E-06
337	TRIM29	0.09	0.11	0.11	0.02	0	0.02	0.14	0.07	0.06	-2.95419631	-0.199308808	0.025992991
338	RCN3	0.4	0.55	0.36	0.05	0.09	0.02	1.12	1.14	1.14	-3.033423002	1.375967935	1.09E-05
339	HLA-DPA	3.39	3.34	3.14	0.38	0.39	0.43	2.78	2.66	2.37	-3.040015679	-0.337727536	4.29E-43
340	YPEL3	0.26	0.18	0.23	0.05	0.02	0.01	0.2	0.16	0.14	-3.06608919	-0.422233001	0.004674547
341	NCKAP1L	0.34	0.38	0.33	0.04	0.03	0.05	0.4	0.51	0.33	-3.129283017	0.239950793	3.04E-11
342	HLA-DRA	1.52	1.83	1.59	0.21	0.03	0.23	1.18	0.98	1.24	-3.39377838	-0.538976295	1.27E-12
343	ZNF671	0.09	0.06	0.1	0	0.01	0.01	0.14	0.13	0.15	-3.64385619	0.748461233	0.043738368

Supplementary Table S2. 343 underrepresented genes in WT EXOSC9-expressing EXOSC9 KD MDA-MB-231.

shRNAs

shLuc (control)	GCAUCACGUACGCGGAAUACCGAAGUAUUCCGCGUACGUGAUG
shEXOSC9#1	GCAAAUACGUGUAGACCUACACGAAUGUAGGUCUACACGUAUUUGC
shEXOSC9#2	GCGUGAUCCUGUACCAUUAAGCGAACUUAAUGGUACAGGAUCACGC
shEXOSC9#3	GCCUUAUGUCAUUUCCGAAGACGAAUCUUCGGAAAUGACAUAAGGC
shEXOSC2#1	GCCUCAGUGAUUCUCGGUAACCGAAGUUACCGAGAAUCACUGAGGC
shEXOSC2#2	GCUGAAGAAUGUGAGAAUUCCCGAAGGAAUUCUCACAUUCUUCAGC
shEXOSC4#1	GGCCCUAGUGAACUGUCAAUACGAAUAUUGACAGUUCACUAGGGCC
shEXOSC4#2	GCUCCCAGAUUGAUAUCUAUGCGAACAUAGAUAUCAAUCUGGGAGC
shAPOBEC3G#1	GGACCAGGACUACAGGGUUACCGAAGUAACCCUGUAGUCCUGGUCC
shAPOBEC3G#2	GCAAUGCACUCCUGACCAAGUCGAAACUUGGUCAGGAGUGCAUUGC

siRNAs

siLuc (control)	GAUCUACUGGUCUGCCUAA
siEDC4#1	CAUAUCACCUGCUGCAGCA
siEDC4#2	CCCAUAUCCUGCAGCUGCU

Supplementary Table S3. List of shRNA and siRNA sequences used in this study

antigen	host species	company	catalgue number	dilution
EXOSC9	mouse	BIO MATRIX RESERCH	BMR00566	1:250 (WB)
EXOSC1	rabbit	Proteintech	12585-1-AP	1:1000 (WB)
EXOSC2	rabbit	Abcam	ab185235	1:1000 (WB)
EXOSC3	mouse	Santa Cruz Biotechnology	sc-166568	1:1000 (WB)
EXOSC4	mouse	Santa Cruz Biotechnology	SC-166772	1:1000 (WB)
EXOSC5	rabbit	Proteintech	15627-1-AP	1:1000 (WB)
EXOSC6	goat	Santa Cruz Biotechnology	sc-107536	1:500 (WB)
EXOSC7	rabbit	Santa Cruz Biotechnology	sc-133570	1:1000 (WB)
EXOSC8	rabbit	Santa Cruz Biotechnology	sc-84108	1:1000 (WB)
EDC4	rabbit	GeneTex	GTX115745	1:200 (IFA/cell), 1:500 (WB)
Dcp1a	mouse	Abcam	ab57654	1:400 (IFA/cell), 1:1000 (WB)
Lsm1	rabbit	LifeSpan BioSciences	LS-C97364	1:200 (IFA/cell), 1:500 (WB)
Xrn1	mouse	Santa Cruz Biotechnology	SC-165985	1:100 (IFA/cell, WB)
eIF4G1	rabbit	Bethyl Laboratories	A300-502A	1:200 (IFA/cell)
V5	mouse	Thermo Fischer Scientific	R960-25	1:5000 (WB), 1:1000 (IFA)
APOBEC3G	rabbit	CST	#43584S	1:500 (WB)
actin	mouse	Wako	0111-24554	1:2000 (WB)
EXOSC10	rabbit	Proteintech	16731-1-AP	1:1000 (WB)
DIS3	rabbit	Proteintech	14689-1-AP	1:500 (WB)
DIS3L	rabbit	AVIVA SYSTEMS BIOLOGY	OAAB03025	1:500 (WB)
HBS1L	rabbit	Proteintech	10359-1-AP	1:1000 (WB)
MPHOSPH6	rabbit	Proteintech	10695-1-AP	1:1000 (WB)
C1D	rabbit	Abcam	AB166897	1:1000 (WB)
RBM7	rabbit	Proteintech	21896-1-AP	1:1000 (WB)
Cleaved caspase-3	rabbit	CST	9665	1:100 (IFA/tissue)
Anti-rabbit IgG, HRP linked whole Ab	donkey	GE Helthcare	NA934V	1:3000(WB)
Anti-mouse IgG, HRP- linked whole Ab	sheep	GE Helthcare	NA931V	1:3000 (WB)
Anti-Goat IgG HRP antibody	rabbit	Sigma	A5420	1:5000 (WB)
Anti-mouse IgG, Alexa Fluor 488	goat	Thermo Fischer Scientific	A-11029	1:1000 (IFA/cell)
Anti-rabbit IgG, Alexa Fluor 488	goat	Thermo Fischer Scientific	A-11034	1:1000 (IFA/cell), 1:500 (IFA/tissue)
Anti-rabbit IgG, Alexa Fluor 594	goat	Thermo Fischer Scientific	A-11037	1:1000 (IFA/cell)

Supplementary Table S4. List of antibodies used in this study.

ACTB	Forward (f)	5′- GAGCTTCCGGAAGAGACCAG -3′
	Reverse (r)	5′- GGATCTCGAAGCTGTCATCG -3′
APOBEC3G	f	TTTGTTGCCCGCCTCTACTAC
	r	TTGGCTGTACACGAACTTGC
ProSELENBP1	f	AGGCATCAAAGAGAGTCTTG
	r	GTGAGTGGAGGTAGTCAGGA
ProRBM39	f	GGAAATAGTGGAGAAAAGCA
	r	CATTTTTGAAGGAACGGTAG
ProRAD50	f	CAGGCAGAGGCAAATTATAC
	r	CACATTTAAGGGCCTAGAGA
ProLACE1	f	TACCCTTCGGATACACTGTC
	r	GGTACGCACTCAAATGTTTT
Proc21orf63	f	TAACACACTTTTCCCCAGAC
	r	ACCACCTAGTACTCCAGCAA
DAPK1	f	GAGAGATTGCTCCCAGTGAGG
	r	CATCAGTCTCCAGTCCTCCAG
PYCARD	f	TCTACCTGGAGACCTACGGC
	r	TCCAGAGCCCTGGTGC
TNFRSF1B	f	CGCCCAGGTGGCATTACA
	r	GCACATCTGAGCTGTCTGGT
TNFRSF21	f	CCCAAAGGCATGAACTCAACA
	r	CCTGAAGGTTTGGGAGGGTC

Supplementary Table S5. qPCR primer sequences used in this study.

Table of data for Fig. 1b

shLuc	7.5	7.4	7.3	7.4	7.45	7.85	6.95	7.3	6.8
shEXOSC9#1	8.5	7.95	7.3	7.95	7.4	6.6	7.35	7.45	7.6
shEXOSC9#2	7.65	7.2	7.2	8.15	7.25	6.55	7.1	7.4	7

Table of data for Fig. 1c

shLuc	6.75	7.5	7.7	6.65	6.75	6.8	7.2	7.7	7.3
shEXOSC9#1	5.3	4.85	4.85	5.4	4.85	5.2	4.95	5.65	4.8
shEXOSC9#2	4.5	4.55	3.6	4.5	5.15	4.3	5	4.65	4.2

Table of data for Fig. 1d

shLuc	4.15	5.1	5.1	4.65	5.2	4.9	4.8	5.2	4.7
shEXOSC9#1	3.65	3.15	3.25	3.65	3.2	3.6	2.8	3.15	3.8
shEXOSC9#2	2.15	2.6	2.8	1.75	2.35	3.4	2.25	2.3	3.1

Table of data for Fig. 1e

shLuc	4.05	4.3	4.85	3.65	4.45	3.9	3.55	4.6	3.95
shEXOSC9#1	2.35	3.25	2.8	2.35	3.4	2.85	2.7	3.4	2.55
shEXOSC9#2	2.25	3.05	2.35	2.4	2.9	2.45	1.7	3.3	2.55

Table of data for Fig. 1f

shLuc	5.65	6.85	6.65	5.6	6.05	7.85	5.45	7.8	7.1
shEXOSC9#1	3.2	3.6	3.55	2.95	3.85	4.15	3.05	3.3	4.75
shEXOSC9#2	3.1	3.35	3.85	2.8	3.55	4.5	4	3.25	4.65

Table of data for Fig. 1h

normal media

shLuc	19	16	16	14	16	6	32	26	23
shEXOSC9#1	17	20	15	21	19	14	34	29	32
shEXOSC9#2	17	22	20	19	16	25	31	27	29

H_2O_2 (4 h)

shLuc	130	163	209	157	128	201	96	111	104
shEXOSC9#1	233	239	252	230	273	237	170	192	267
shEXOSC9#2	249	352	228	205	225	194	259	162	237

Serum free (24 h)

shLuc	114	232	159	126	129	101	94	92	118
shEXOSC9#1	447	634	436	244	243	318	262	241	141
shEXOSC9#2	922	620	348	247	283	242	148	203	237

Table of data for Fig. 2a

shLuc		shEXOSC9#1		shEXOSC9#2	
8	2	0	10	2	0
7	0	0	3	2	0
3	1	7	0	5	1
1	4	0	7	3	0
0	3	0	0	0	0
0	0	2	0	0	0
4	1	0	0	0	1
0	3	0	2	1	0
9	2	0	0	0	3
2	7	0	0	0	6
7	0	0	6	0	1
2	2	4	0	0	0
8	0	0	5	0	0
3	0	10	0	0	0
3	8	0	6	3	0
1	0	4	0	0	4
8	4	7	4	0	0
2	8	0	5	0	1
1	2	0	0	0	3
0	5	0	0	0	1
5	0	0	0	0	1
5	7	0	0	0	5
4	2	0	2	0	3
0	5	0	0	5	0
4	4	4	0	0	0
4	2	0	0	0	0
9	0	0	0	5	0
0	7	0	3	0	0
3	8	0	0	2	1

4	6	0	4	8	1
2	0	4	4	0	0
0	1	0	0	0	0
1	0	0	0	0	0
3	0	3	0	0	5
4	1	6	0	0	6
0	3	0	0	0	1
2	3	0	0	0	4
4	4	9	0	0	2
6	7	0	1	2	5
3	0	2	5	2	0
1	4	0	0	0	0
4	2	0	0	0	0
3	4	0	0	0	0
11	0	6	0	0	0
0	0	0	5	5	5
1	7	0	0	3	2
3	1	0	7	0	6
0	13	7	4	0	0
16	4	1	0	0	0
0	0	0	3	0	0

Table of data for Fig. 2b

shLuc		shEXOSC9#1		shEXOSC9#2	
0	4	2	0	3	0
0	1	1	3	0	8
14	3	0	1	4	0
1	3	0	0	1	3
0	2	0	1	0	7
0	0	3	0	0	0
0	0	0	0	5	0
2	1	7	0	0	5
1	0	1	0	0	0
6	3	0	3	0	0
4	6	0	0	0	0
0	0	0	0	0	0
0	1	0	0	0	0
0	7	0	0	0	1
2	0	0	6	0	3
7	3	0	9	0	2
0	4	0	0	0	0
0	4	1	1	2	0
13	5	0	0	2	1
0	0	0	0	0	3
0	0	0	3	4	5
5	0	0	0	0	3
2	0	1	4	0	0
0	3	2	0	0	0
0	1	0	0	0	2
0	4	0	0	3	0
5	1	1	0	0	3
0	0	8	1	3	0
3	0	0	0	0	0
0	0	0	0	0	0
6	4	3	0	0	3
4	4	0	0	0	0
0	3	5	1	0	0
4	4	3	3	1	4
0	0	10	0	0	0
0	4	1	0	0	0
0	0	3	0	0	0
4	3	0	0	0	3
0	4	0	0	0	1
10	5	0	0	5	0
2	6	4	3	0	0
1	2	0	0	0	0
2	0	0	0	0	0
0	2	3	5	0	0
0	4	0	0	0	4
0	6	0	0	0	0
3	2	0	0	0	0
2	0	0	0	0	9

1	0	1	0	2	6
6	0	1	3	4	4

Table of data for Fig. 2c

shLuc		shEXOSC9#1		shEXOSC9#2	
4	0	0	0	4	0
0	4	0	2	5	1
0	3	2	0	0	0
3	9	6	0	0	0
4	3	0	0	0	4
4	1	0	0	0	1
1	8	1	0	2	0
5	0	0	1	0	0
1	0	1	0	0	0
0	3	1	4	1	0
2	3	4	2	0	0
0	3	0	4	3	0
0	7	5	0	0	0
4	4	4	10	0	0
0	0	0	0	1	0
2	2	0	0	0	0
0	1	0	0	2	0
6	1	0	1	0	0
3	0	0	0	1	1
5	0	0	0	0	0
4	4	0	0	1	0
3	1	0	0	6	4
4	0	2	0	2	2
0	2	0	0	0	1
2	0	0	1	2	3
4	3	4	0	0	2
4	2	1	0	4	0
4	5	0	0	0	0
1	4	0	0	4	0
4	0	0	5	0	1
0	0	0	0	0	2
1	5	0	5	0	0
0	2	0	0	0	2
0	1	0	0	0	0
4	2	1	0	0	0
0	3	0	0	0	0
2	2	0	0	0	0
2	0	0	3	0	0
3	0	0	1	0	0
0	4	0	0	0	0
5	0	0	1	8	0
4	3	0	0	1	3
0	0	1	4	5	0
5	5	0	0	5	0
1	4	2	2	0	4
6	0	4	0	0	0
3	0	1	0	2	0
4	2	0	0	0	1
0	6	0	3	0	5
8	2	0	0	0	2

Table of data for Fig. 2d

shLuc		shEXOSC9#1		shEXOSC9#2	
0	0	0	0	0	6
5	7	0	0	0	0
0	0	0	9	0	3
3	0	0	0	3	0
0	3	0	0	7	0
0	0	0	0	1	0
0	3	0	0	0	0
0	0	3	0	0	0
6	2	0	2	0	1
0	6	2	0	4	1
0	5	0	0	0	0
2	2	1	0	2	0
1	0	10	0	0	4
2	2	0	0	3	0
5	4	0	1	0	1

3	0	0	0	0	0
0	0	0	0	0	0
0	4	0	4	3	0
1	4	0	0	0	0
4	0	0	0	8	0
4	5	0	5	0	0
1	0	0	0	3	6
6	3	0	0	1	0
6	0	0	0	0	0
0	0	0	4	0	1
9	0	0	0	1	0
0	2	0	0	0	6
0	4	0	0	0	2
0	2	0	0	0	1
5	3	0	2	0	0
0	2	0	0	0	0
0	2	0	0	0	0
0	0	0	0	4	2
4	6	2	0	4	6
4	2	4	0	0	3
2	1	0	4	0	0
1	2	4	3	0	2
2	5	0	0	0	0
0	7	1	0	0	0
2	2	0	0	4	3
0	2	0	0	0	0
1	0	5	0	0	0
2	0	1	0	5	3
5	0	0	4	0	4
2	0	3	0	0	0
1	2	0	0	0	2
4	2	3	1	0	0
2	1	0	4	0	0
7	2	0	0	4	0
0	0	0	0	0	1

Table of data for Fig. 3a

shLuc		shEXOSC9#1		shEXOSC9#2	
0	4	0	0	0	3
3	1	0	2	7	0
3	0	0	1	0	0
1	0	0	2	0	0
4	6	0	0	0	0
2	3	0	2	0	0
3	0	0	7	0	5
9	2	3	2	0	0
4	3	0	1	0	0
1	1	0	3	0	0
3	3	10	2	0	0
2	2	0	0	0	0
4	10	4	0	0	2
4	0	0	0	0	0
4	0	0	0	0	4
3	3	3	2	0	0
2	2	0	4	0	2
1	6	0	0	0	0
5	3	0	6	0	0
3	4	0	0	1	0
4	2	1	4	0	0
5	2	4	0	0	0
8	3	3	1	2	0
2	4	0	0	0	0
0	6	0	3	7	0
0	3	0	0	9	0
1	5	0	0	0	1
3	3	2	4	3	4
3	0	0	0	0	0
0	1	0	3	1	7
1	0	0	1	1	0
5	4	1	4	0	3
2	5	0	0	3	0
1	2	1	1	0	0
3	0	0	0	0	1

2	0	0	0	1	2
4	5	4	0	0	0
5	6	5	0	0	0
1	2	0	1	0	3
8	5	2	1	0	0
3	1	0	1	0	2
1	5	0	1	0	0
1	3	0	0	0	0
4	2	1	0	0	0
2	2	3	1	0	0
5	7	0	0	0	0
5	1	4	2	1	0
1	1	0	0	0	0
0	1	0	3	0	5
4	6	0	0	0	0

Table of data for Fig. 3b

shLuc		shEXOSC9#1		shEXOSC9#2	
1	11	3	0	1	1
5	4	2	0	0	1
3	5	0	2	0	0
6	6	2	0	0	0
0	9	3	7	0	0
3	5	0	0	0	0
0	1	0	2	0	0
0	0	0	2	0	0
9	3	0	1	0	0
6	0	0	9	0	3
0	5	1	3	0	0
3	3	6	0	4	0
5	4	0	0	0	1
0	4	0	0	0	0
6	6	0	16	0	2
2	0	0	0	3	2
7	9	5	0	0	0
3	2	0	4	0	0
1	2	1	0	1	0
2	5	0	0	0	0
2	8	4	0	2	0
12	6	0	0	2	0
8	0	1	0	1	0
3	0	1	0	0	0
5	4	3	2	0	0
8	5	0	0	0	0
0	4	0	3	0	0
6	4	0	2	0	5
0	3	0	3	2	4
0	0	0	0	3	0
4	0	5	0	0	4
3	0	0	0	0	0
6	0	0	0	0	0
9	4	0	0	0	0
0	5	0	2	0	0
9	0	0	0	0	0
1	7	0	3	0	0
0	0	0	1	1	0
0	2	0	0	0	2
0	5	0	0	0	0
3	4	0	0	0	0
0	12	3	0	3	0
4	16	0	3	0	3
0	9	0	0	0	0
7	1	0	0	0	0
4	6	0	2	0	0
2	5	0	4	0	0
11	8	0	1	3	0
0	7	0	0	0	0
6	4	0	4	0	0

Table of data for Fig. 3c

shLuc		shEXOSC9#1		shEXOSC9#2	
0	2	0	0	1	0
2	0	0	0	0	0

0	2	0	2	0	3
0	0	1	0	0	0
2	0	0	0	1	0
4	2	0	0	5	0
0	1	0	0	0	0
0	0	0	0	0	0
3	0	0	0	0	0
2	0	0	1	0	0
10	0	0	0	0	0
0	0	0	0	0	0
0	0	0	2	1	0
2	0	0	0	0	1
7	1	0	0	8	0
3	0	0	0	0	0
0	3	3	0	0	0
0	0	0	1	0	0
3	0	0	0	0	0
0	0	0	1	0	0
0	0	0	0	7	0
0	0	2	0	0	2
0	0	1	0	0	1
10	2	3	2	4	0
0	0	0	0	1	0
1	2	0	0	0	1
0	0	0	0	0	1
3	0	1	0	5	1
0	0	0	0	0	0
0	0	0	0	3	4
1	0	2	0	0	0
0	0	0	0	0	1
0	0	2	0	0	0
1	1	0	0	0	0
0	1	0	0	0	0
2	0	0	0	1	0
0	0	0	0	0	0
6	0	0	0	0	0
0	0	3	0	0	2
1	0	0	2	0	0
2	2	0	0	0	0
0	2	0	0	0	0
7	0	0	0	0	0
0	4	0	0	0	0
2	3	0	0	0	1
3	3	4	0	0	0
0	0	0	0	1	0
2	4	0	0	0	0
0	2	0	0	1	1
2	0	0	0	0	0

Table of data for Fig. 3d

shLuc		shEXOSC9#1		shEXOSC9#2	
2	2	7	0	5	0
11	13	0	4	0	2
3	4	0	0	0	0
4	4	0	0	0	0
0	2	4	0	0	0
3	0	9	2	4	0
1	7	1	0	0	0
2	4	0	0	0	0
5	0	1	0	5	5
0	4	9	2	3	0
5	3	4	0	3	0
10	9	0	1	2	0
7	6	0	3	0	7
3	1	3	2	0	3
0	0	0	0	0	0
10	4	0	0	0	0
7	4	5	8	5	0
15	2	2	0	7	0
8	5	0	0	0	4
8	11	1	0	0	0
9	4	0	0	3	0
0	0	0	6	1	3

5	6	4	0	0	2
12	5	0	0	6	5
7	3	0	4	0	1
5	8	0	1	3	1
4	31	0	7	0	7
8	4	0	7	0	4
5	8	0	4	0	0
7	3	0	0	0	2
17	5	3	2	7	0
6	7	9	0	0	3
0	0	0	0	2	0
5	7	3	3	2	3
10	0	0	9	0	0
10	3	1	2	0	0
4	8	6	0	1	0
5	7	0	0	3	8
6	9	3	14	1	0
5	3	2	0	0	0
4	5	0	0	0	4
4	5	2	12	0	0
7	7	1	4	0	1
4	4	0	0	0	0
11	4	0	0	4	0
0	22	3	0	8	1
0	5	3	0	0	0
2	5	0	0	0	0
7	12	0	2	8	4
0	35	0	0	0	0

Table of data for Fig. 3f

shLuc		shEXOSC9#1		shEXOSC9#2	
13	10	7	5	6	3
12	16	0	0	6	6
14	7	2	0	6	0
22	18	8	0	0	4
20	11	8	0	5	0
4	9	0	0	4	7
16	12	8	0	6	8
0	15	7	4	4	4
5	0	0	0	7	4
8	7	10	4	5	7
7	9	7	5	5	7
10	9	5	5	0	0
9	8	0	6	2	8
14	19	8	4	0	8
7	10	5	6	5	3
16	15	6	0	3	0
14	13	5	6	8	5
0	6	0	4	0	6
14	12	0	8	7	10
0	20	4	4	9	9
5	8	0	5	4	0
12	11	6	0	10	12
5	11	0	5	7	12
14	14	6	3	0	8
4	15	4	0	3	4
4	14	5	0	4	6
0	5	0	9	5	6
11	24	4	0	0	8
13	14	0	6	12	0
9	12	8	0	8	7
9	17	4	7	5	5
12	8	6	9	5	5
8	18	0	3	5	0
8	12	0	6	14	0
11	18	0	6	0	9
0	9	5	3	5	5
17	19	7	10	0	7
12	20	7	6	8	5
15	17	5	2	4	8
15	16	5	2	5	6
14	0	0	7	2	4
10	9	6	5	0	4

0	20	0	8	4	3
8	15	9	7	6	9
10	9	4	5	0	8
13	17	5	0	5	6
8	21	9	5	0	11
0	6	7	7	6	8
10	30	4	7	7	14
5	15	0	7	8	14

Table of data for Fig. 3g

shLuc		shEXOSC9#1		shEXOSC9#2	
0	3	10	12	7	10
23	0	8	8	0	10
18	0	9	2	10	4
0	8	8	3	8	7
4	33	8	4	7	6
17	12	8	5	9	10
3	16	4	0	8	7
22	11	5	6	14	8
8	1	7	17	0	4
5	0	4	5	44	8
3	2	5	4	6	6
16	4	7	6	6	11
8	16	0	7	6	16
6	18	4	16	11	6
13	4	8	13	13	8
4	4	0	3	7	9
10	0	11	0	6	14
11	0	10	8	3	12
1	6	0	0	8	0
7	12	8	11	6	12
26	13	8	3	5	5
7	4	5	3	9	25
12	9	10	4	5	11
10	18	6	12	3	8
1	21	9	9	9	7
8	10	0	10	6	12
11	10	6	12	5	6
0	0	16	5	8	4
0	11	0	8	5	8
16	3	8	9	14	16
0	6	6	5	16	10
6	3	4	3	13	5
10	7	6	23	14	11
0	0	4	12	14	5
8	10	16	19	8	11
8	12	10	7	14	6
5	14	4	5	7	12
20	4	10	6	10	9
12	5	3	9	5	8
14	4	5	0	8	0
9	0	8	0	6	0
12	17	5	5	24	10
8	2	10	5	0	18
7	0	8	14	16	10
8	16	0	4	8	18
12	8	6	10	11	9
12	7	8	7	0	10
6	14	8	5	12	22
15	5	3	8	4	8
8	6	15	9	7	8

Table of data for Fig. 4b

shLuc	8.2	7.3	7.2	8.2	8.05	7.45	7.3	7.65	7.45
shEXOSC2#1	5.8	5.9	6.5	6.1	5.9	6.45	6.8	6.3	6.75
shEXOSC2#2	6.2	5.15	6.5	6.3	6.3	6.3	6.8	6.3	6.45
shEXOSC4#1	4.8	5.15	6.3	4.6	4.5	5.85	4.8	4.7	5.85
shEXOSC4#2	6	5.15	4.5	6.2	4.95	5.15	5.8	4.05	4.95

Table of data for Fig. 4c

shLuc	7.2	7.65	7.45	7.6	6.3	6.3	7.8	6.9	6.1
shEXOSC2#1	3.6	4.25	3.85	4.6	4.5	5.15	4.2	3.6	4.25
shEXOSC2#2	4.8	3.15	4.05	4.6	4.7	4.5	5.6	5.4	4.25
shEXOSC4#1	2.2	2.95	4.5	2.2	2.7	4.25	3.4	3.6	4.3
shEXOSC4#2	3.6	4.05	4.05	5	2.9	3.8	4.4	2.9	3.6

Table of data for Fig. 4d

shLuc	6.8	6.95	7.9	7.6	7.95	7.45	7.2	6.9	7.65
shEXOSC2#1	4.6	5.5	5.85	5.2	4.5	5.15	4.4	4.75	4.7
shEXOSC2#2	5.2	5.25	6.05	4.8	4.5	5.15	5.6	4.5	4.95
shEXOSC4#1	2.4	3	4.7	2.8	4	4.5	3.4	4.5	4.7
shEXOSC4#2	5	4.5	4.5	4.6	3.5	3.35	4.2	3.75	3.8

Table of data for Fig. 4e

shLuc	3.6	3.25	4.3	3.4	3.5	4.25	3.2	3.75	4.4
shEXOSC2#1	1.6	1.5	2.7	1	1.75	3.15	1.4	1.75	3.35
shEXOSC2#2	2.6	2	2.25	2	1.5	2.5	2	2.5	2.25
shEXOSC4#1	1.6	1	2.05	1.8	1.5	1.8	1.6	1.25	2.45
shEXOSC4#2	1.6	2.25	1.8	1.8	0.75	1.6	2.2	1.5	1.65

Table of data for Fig. 4f

shLuc	8	7.25	5.4	6.8	9.45	6.55	7	9	6.6
shEXOSC2#1	5	4.7	4.05	5.4	3.8	3.6	5.2	2.7	3.35
shEXOSC2#2	4.4	4.95	4.5	5.2	5.85	4.25	5.8	4.95	4.05
shEXOSC4#1	4	4.5	4.05	3.2	3.8	2.95	2.6	3.6	3.85
shEXOSC4#2	4.4	4.5	4.05	5.6	3.8	3.35	4.6	4.95	3.15

Table of data for Fig. 4g

shLuc		shEXOSC2#1		shEXOSC2#2		shEXOSC4#1		shEXOSC4#2	
9	0	4	2	2	0	5	2	3	2
0	1	4	4	3	0	0	0	0	0
1	2	3	9	2	3	3	0	0	1
0	14	3	1	0	1	0	3	3	1
3	8	4	0	3	1	5	0	0	0
0	0	1	5	2	2	2	0	0	1
3	0	2	5	1	1	2	4	0	2
2	21	0	2	0	0	4	2	1	1
5	0	2	4	2	2	2	3	0	1
5	1	0	2	0	0	6	2	0	0
36	0	2	3	0	0	0	5	0	4
10	1	0	4	1	4	0	2	0	2
9	4	0	0	0	0	5	0	0	0
4	4	0	0	2	0	0	0	2	1
4	1	3	4	2	2	3	0	1	4
0	4	2	2	4	4	2	0	0	1
3	14	3	3	5	1	2	24	0	1
3	6	2	1	2	4	1	3	1	2
0	4	0	6	2	0	0	0	0	4
5	3	0	0	0	0	4	0	1	0
3	2	3	4	0	0	0	2	1	2
4	0	0	0	2	1	1	1	0	0
4	2	3	1	3	2	1	0	3	2
2	3	5	0	2	0	2	4	0	0
3	0	0	0	0	0	5	6	1	0
5	3	3	5	6	2	0	3	3	2
3	3	0	2	2	4	3	4	4	1
9	3	0	2	0	3	2	4	15	2
5	4	1	2	2	0	4	3	0	0
0	3	3	0	2	2	2	1	4	0
6	6	3	0	0	5	0	1	0	1
4	2	2	2	2	6	1	2	3	0
4	3	0	0	2	5	0	2	3	1
5	4	0	0	2	0	2	3	4	4
2	3	0	0	3	0	0	5	4	11
12	0	0	0	3	2	1	0	3	3
4	3	0	0	8	0	2	3	0	0
3	4	2	0	2	0	4	1	0	0
0	4	0	0	0	0	4	0	1	3
6	4	2	0	0	0	0	0	2	4

5	8	4	0	4	0	2	0	2	4
24	4	0	1	0	0	1	4	0	0
4	4	0	0	3	0	0	1	2	6
19	0	0	2	0	0	2	0	0	1
4	1	2	1	5	1	0	0	4	0
0	4	1	2	0	2	5	2	0	3
5	28	0	2	3	2	0	4	0	0
7	3	1	0	0	1	3	3	1	0
4	2	0	4	0	2	0	3	27	1
2	0	0	1	14	0	3	2	0	1

Table of data for Fig. 5c

mock	7.4	7	7.05	7.3	8.05	8.45	6.85	7.25	8.4
WT	6.75	7.4	7.1	6.4	7.85	7.4	7.9	7.3	8.6
MUT	7.2	7.35	8.6	8.15	7.5	7.9	7.3	7.55	8.6

Table of data for Fig. 5d

mock	3.8	4.75	5.35	4	4.85	5.15	3.65	4.5	4.8
WT	5.75	6.5	6.95	6.25	7.25	7	5.5	6.65	6.85
MUT	4	3.7	4.3	3.8	4.65	4.95	4.2	3.95	4.5

Table of data for Fig. 5e

mock	3.5	4.25	3.3	3.55	4.2	2.95	2.85	4.55	3.05
WT	6.3	6.4	4.55	5.25	6.7	4.4	6.55	7.15	4.45
MUT	3	4	3.15	3.2	5.3	2.8	3.4	4.35	3.2

Table of data for Fig. 5f

mock	3.75	3.35	3.8	3.65	3.5	3.3	3.8	3.8	3.25
WT	5.7	4.05	5.25	5.75	5.25	5.05	5.95	5.15	4.4
MUT	3.95	2.7	2.9	3.4	2.85	3.2	4.95	3.45	3.4

Table of data for Fig. 5g

mock	4.45	4.35	4.25	4.1	3.7	3.75	3.65	4.55	3.5
WT	7.35	5.15	4.85	6.2	5.85	5.75	6.4	5.85	6.15
MUT	3.75	3.3	3.75	4.35	3.25	3.8	3.6	4.25	4.4

Table of data for Fig. 5h

mock		WT		MUT	
6	0	0	0	0	3
0	6	3	5	0	1
0	0	0	5	0	0
0	0	2	0	0	0
1	0	9	3	0	0
0	5	0	0	0	0
2	4	4	3	0	0
3	5	11	2	0	0
0	2	0	1	0	0
5	2	8	4	0	0
2	4	16	0	0	6
0	0	2	0	0	0
1	4	0	5	0	0
4	0	5	0	0	0
0	5	0	4	4	0
0	0	4	2	0	5
3	3	0	2	0	0
0	8	1	0	0	0
0	0	9	8	0	0
0	0	0	0	4	2
0	4	7	6	2	0
0	1	5	0	7	0
0	0	4	1	1	0
0	0	0	0	4	9
0	0	5	0	7	0
4	4	0	0	8	3
0	0	0	5	2	1
2	0	0	0	0	3
0	3	19	2	0	0
0	0	10	2	12	0
2	0	4	1	7	1
8	0	0	7	0	0
1	1	0	5	0	0
0	3	4	0	0	3

0	0	0	0	4	1
0	0	0	3	0	0
2	3	4	0	0	2
0	3	0	0	0	0
4	4	0	2	0	0
2	4	0	6	0	0
0	3	0	2	0	0
3	4	4	1	0	0
6	0	5	1	2	0
0	1	2	0	0	1
0	0	3	5	0	0
0	2	5	7	0	0
0	0	0	11	0	0
3	0	6	2	0	0
0	0	4	6	1	0
0	0	5	5	0	0

Table of data for Fig. 5i

mock		WT		MUT	
0	5	0	5	3	4
0	0	4	8	0	0
0	0	3	3	3	1
0	2	4	6	0	0
0	7	1	1	3	0
0	0	5	0	3	0
6	0	15	5	0	0
3	2	12	10	0	4
0	8	0	5	0	0
0	3	1	0	2	0
0	0	1	8	0	2
0	0	4	3	3	7
0	0	3	5	0	0
4	4	2	0	0	0
0	0	0	7	0	5
0	0	3	1	6	4
0	0	9	0	0	0
4	0	7	3	0	4
5	6	0	0	3	4
0	0	4	1	0	0
0	0	8	0	2	0
0	2	1	0	0	4
0	2	0	2	1	0
0	10	0	0	0	0
0	4	0	0	0	1
0	6	0	2	0	8
0	10	9	0	0	7
8	1	0	3	2	0
0	2	0	0	0	0
0	5	3	0	0	0
0	0	12	0	5	2
1	0	12	1	0	0
4	5	4	14	0	0
0	0	0	0	0	1
0	2	0	6	1	0
0	0	13	9	2	0
2	0	0	0	4	0
2	2	8	6	6	2
0	0	0	11	0	0
0	1	2	1	7	4
0	0	2	16	1	1
0	3	9	0	0	0
0	6	0	8	2	0
0	0	1	19	0	0
5	0	0	10	0	3
0	3	0	0	0	4
0	3	11	3	0	1
0	0	0	0	0	0
0	0	5	0	2	0
0	0	0	3	0	0

Table of data for Fig. 5j

mock		WT		MUT	
4	0	0	2	0	0

0	0	0	6	4	0
2	0	0	2	0	0
2	0	4	4	0	1
0	0	4	0	0	0
1	0	2	0	0	0
3	5	9	0	0	3
0	3	0	0	1	0
0	0	4	0	0	0
0	1	0	0	0	0
2	0	0	0	0	1
0	0	5	0	0	0
2	1	4	3	0	0
2	0	0	0	0	0
0	1	0	0	2	0
0	0	1	2	5	0
3	0	0	0	4	0
0	1	0	4	0	4
0	2	4	1	0	5
0	0	3	4	5	0
0	0	1	0	0	0
0	0	4	0	0	0
0	4	0	3	0	4
0	0	0	0	0	0
0	0	0	0	0	0
3	0	0	0	0	3
2	0	0	0	0	0
2	0	0	0	0	0
0	0	4	7	0	1
3	4	12	0	0	0
0	0	0	0	1	0
0	3	0	2	0	0
1	0	0	0	4	1
0	0	0	0	0	2
0	0	0	0	0	0
0	0	7	0	4	0
0	0	0	0	0	0
0	0	0	1	0	3
0	3	3	5	0	0
0	1	0	9	0	0
0	0	1	5	0	0
1	0	0	2	2	0
0	1	5	2	0	0
3	1	4	0	0	0
0	0	0	0	0	0
0	1	6	0	1	0
1	0	0	3	0	0
0	0	0	3	0	5
0	0	0	0	0	0
2	0	0	2	0	3

Table of data for Fig. 5k

mock		WT		MUT	
0	0	0	0	1	6
0	1	0	0	2	0
0	0	4	5	0	0
3	0	5	0	0	1
0	0	3	0	0	0
2	0	3	3	0	0
0	0	0	6	0	0
0	0	3	0	0	0
0	0	7	4	0	0
0	0	4	7	0	0
0	0	3	0	0	0
0	4	4	3	0	0
4	0	1	3	0	0
0	0	0	0	0	0
4	4	0	0	0	0
0	0	0	1	0	0
1	0	0	3	2	2
0	2	3	0	0	1
0	0	0	6	4	0
0	0	0	2	0	1

0	0	4	0	0	0
5	2	0	2	0	0
0	0	0	5	0	0
5	0	0	5	0	0
0	0	0	0	0	0
0	2	4	5	0	0
0	0	1	0	0	0
0	0	1	0	0	0
0	4	0	0	0	0
4	4	6	1	3	0
0	0	0	8	0	0
4	0	0	0	0	0
0	0	1	4	0	3
2	0	6	0	0	1
3	1	0	0	0	5
0	2	0	0	0	0
0	0	0	2	4	0
0	6	2	0	2	8
0	1	20	5	0	0
3	0	0	0	0	3
0	1	3	2	0	4
0	0	0	6	0	0
6	0	0	7	0	0
0	0	3	0	0	0
1	5	0	0	0	0
0	0	0	0	8	5
0	3	5	0	0	0
0	0	0	4	0	0
0	0	0	13	0	0
0	0	0	0	4	0

Table of data for Fig. 6a

mock	97.26	103.60	99.14
WT	35.49	29.63	41.05
MUT	99.44	82.35	82.52

Table of data for Fig. 6c

	APOBEC3G			ACTB		
mock	0.00278	0.00253	0.00283	0.00296	0.00180	0.00194
WT	0.01007	0.00988	0.01148	0.00193	0.00222	0.00196
MUT	0.00242	0.00341	0.00351	0.00291	0.00272	0.00290

Table of data for Fig. 6d

	0	1	2	4	8 (h)
mock	130.79	87.56	90.04	43.91	24.14
	93.54	55.86	144.41	92.79	42.75
	75.67	109.25	78.90	84.00	23.50
WT	79.57	77.19	65.83	7.00	5.76
	91.93	93.00	28.20	31.31	3.59
	128.50	92.06	71.35	47.77	1.24
MUT	85.57	90.94	88.94	79.39	22.80
	106.56	111.20	72.38	111.25	34.71
	107.87	65.84	61.86	77.07	17.60

Table of data for Fig. 6f

shLuc	7.35	8.5	8.4	8.05	8.05	8.5	7.1	7.45	7.55
shAPOBEC3G#1	7.7	7.75	7.45	7.1	7.7	8	7.65	9.3	7.1
shAPOBEC3G#2	7.9	7.35	7.5	7	9.45	8.15	7.45	7.55	7.15

Table of data for Fig. 6g

shLuc	4.95	5.1	5.35	5.65	5.9	5.15	6.1	5.75	5.35
shAPOBEC3G#1	8.35	8.3	7.5	8.05	7.65	6.5	7.2	7.75	7.5
shAPOBEC3G#2	7.6	7.3	6.5	8.3	8	8.15	8.05	7.8	7.35

Table of data for Fig. 6h

shLuc	5.7	5.45	4.95	5.55	5.9	5.35	5.85	6.1	5.35
shAPOBEC3G#1	7.75	9.25	6.5	7.75	8.25	5.8	7.3	8.8	6.55
shAPOBEC3G#2	6.7	7.7	7.3	7	8.45	7.75	7.2	8	7.2

Table of data for Fig. 6i

shLuc	3.05	3.5	2.6	3.7	4.1	2.95	2.95	3.7	3.05
shAPOBEC3G#1	4.6	5.5	3.8	5.1	6.3	3.75	4.6	5.7	4.65
shAPOBEC3G#2	4.9	5.55	4.05	5.3	5.5	4.8	4.5	5.4	3.65

Table of data for Fig. 6j

shLuc	4.5	4.3	5.05	4.75	4.05	5.15	4.5	4.3	4.9
shAPOBEC3G#1	6.25	6.7	8.15	5.4	6	6.3	6.55	6.25	6.7
shAPOBEC3G#2	7.75	7.15	6.05	6.8	7.05	6.95	6.95	8.4	6.9

Table of data for Fig. 6k

shLuc		shAPOBEC3G#1		shAPOBEC3G#2	
1	0	2	3	3	3
4	3	7	7	4	1
0	0	10	4	3	8
1	0	4	3	3	3
0	2	0	1	3	2
0	0	8	4	2	3
0	2	0	0	0	0
0	2	1	6	0	5
0	4	4	3	5	2
5	0	5	2	7	9
4	4	3	0	2	8
0	0	4	2	5	0
4	0	3	6	1	2
4	1	2	1	4	0
0	0	1	0	7	9
0	0	5	5	2	0
0	3	2	0	4	2
0	0	0	2	4	7
1	0	5	1	2	4
0	3	0	2	1	8
1	0	4	0	0	7
0	4	5	1	4	2
2	0	2	4	0	6
0	0	2	1	3	3
1	0	8	8	3	14
0	3	2	1	8	3
1	0	4	3	0	5
2	0	2	2	4	7
0	1	5	0	0	4
0	0	1	0	5	5
0	4	2	2	1	3
0	0	6	6	4	3
0	0	1	4	3	6
0	5	4	11	3	1
0	1	2	0	3	3
0	0	9	4	1	4
6	0	2	1	8	6
0	6	5	0	0	4
2	3	10	11	8	9
2	1	0	4	3	1
10	3	7	0	4	3
0	3	2	0	6	0
0	4	1	0	2	3
0	0	2	0	5	3
10	0	5	0	3	3
1	0	0	1	6	3
5	0	7	1	6	1
0	0	4	4	7	2
0	0	6	0	0	5
2	0	4	1	4	4

Table of data for Fig. 6l

shLuc		shAPOBEC3G#1		shAPOBEC3G#2	
2	0	0	0	8	1
0	0	15	6	2	0
0	3	3	4	5	0
2	5	2	0	4	0
3	2	8	4	9	0
0	3	4	0	10	0
0	3	0	5	0	2

4	4	0	3	3	0
3	0	3	12	2	1
1	0	7	5	4	0
4	0	0	4	3	5
0	0	0	7	3	10
3	0	0	3	3	4
4	1	4	6	10	1
6	0	3	0	9	3
4	0	5	4	1	0
2	3	5	0	5	4
1	3	4	3	3	2
0	4	0	2	3	1
0	1	2	11	4	10
0	2	0	2	2	2
0	0	6	3	0	12
0	3	0	3	4	4
2	2	0	4	0	0
3	0	1	2	4	6
1	0	4	5	3	4
0	3	4	7	1	1
0	3	3	9	2	8
3	0	1	0	0	4
6	3	8	1	3	2
0	0	2	7	0	0
0	1	0	4	3	7
5	0	8	4	4	8
1	3	0	1	1	5
1	2	3	5	0	5
0	1	1	5	3	5
3	3	4	7	7	3
0	0	4	4	10	6
0	7	1	4	4	4
0	2	2	0	0	0
1	2	0	6	2	3
0	6	3	3	5	3
3	2	3	0	5	2
0	0	5	2	6	5
0	0	1	5	0	3
0	0	1	3	0	8
0	0	0	2	2	4
0	1	4	8	0	0
1	2	1	4	0	12
3	0	1	4	5	5

Table of data for Fig. 7b

	7	10	13	16	19	22	25 (days)
shLuc	52.06679	182.0008	359.4345	514.9783	768.9692	958.8326	1179.402
	22.10229	61.26152	85.44859	114.6882	235.8726	324.9891	458.6422
	72.41717	260.6718	521.0163	626.9756	877.1388	913.8016	1213.927
	67.89837	251.8894	417.2656	535.3892	873.8857	1104.88	1533.634
	60.26922	262.1868	585.5123	774.2688	875.2304	1115.697	1543.514
	53.55137	181.0106	326.6328	511.5433	721.2328	1026.98	1388.779
shEXOSC9#1	43.1695	49.59111	59.73757	153.6601	192.5949	308.8315	466.5113
	63.24912	131.933	179.6819	312.8265	498.9993	489.2626	745.1766
	43.09633	44.16281	60.81709	149.3394	170.333	281.0801	412.2891
	38.72555	36.11611	44.41886	117.8472	160.2493	253.5076	320.845
	17.89841	34.84888	28.04342	86.53449	125.9766	236.6217	327.8267
	57.71248	90.91446	72.9944	235.0878	332.0133	424.5544	538.361
shEXOSC9#2	34.17925	33.76	61.70494	105.5115	173.051	261.2003	366.0971
	24.65157	10.49748	8.593272	18.75346	19.15866	20.43844	39.25881
	24.02221	3.15751	5.334701	6.37445	14.3971	8.793663	22.11458
	29.602	20.70263	23.06491	58.97912	96.28325	152.3743	169.3786
	32.68698	28.15	42.65462	89.7449	98.06782	165.6036	233.7188
	12.74	2.454232	8.8218	11.2408	15.94611	30.8183	59.6781

shLuc	shEXOSC9	shEXOSC9#2
0.235	0.792	0.992
0.185	2.091	1.259
0.254	1.191	0.99
0.774	1.522	1.06
0.309	0.932	1.641
0.438	0.6	1.001

0.055	1.543	0.902
0.142	2.669	0.314
0.082	2.06	1.307
0.125	1.205	0.402
0.085	1.002	0.959
0.013	0.907	0.219
0.263	0.64	0.45
0.029	2	0.471
0.029	0.504	0.709
0.265	2.536	0.082
0.072	4.358	0.114
0.152	0.555	0.108

Table of data for Fig. 7f

	7	10	13	16	19	22	25 (days)
mock	24.23645	17.74094	20.71035	46.78701	109.2359	153.9727	169.3639
	27.36229	18.28928	46.17128	49.9753	191.785	332.4012	312.8294
	31.73615	17.6967	16.58704	42.98875	55.30383	47.57746	109.3448
	20.24756	8.825195	6.1017	16.26834	31.07707	261.1541	274.5293
	34.6357	46.97837	56.6766	167.8199	201.9809	356.911	346.1328
	25.53086	15.63581	7.961526	32.80726	63.42077	129.4015	138.8778
WT	53.77039	58.03983	91.14147	155.7957	219.4168	384.7142	571.1652
	64.84604	219.4672	193.3464	329.9115	475.8702	688.4089	1061.941
	62.5128	62.03051	169.0049	204.3116	362.3162	686.6718	905.3322
	55.9793	106.2894	183.419	629.7701	1120.201	1744.216	2128.033
	54.30407	95.1217	94.1453	170.8093	317.4303	498.8143	592
	38.19902	35.23801	91.63265	189.3984	344.64	385.1986	400.7591
MUT	33.3234	25.34515	47.7224	106.5184	170.8374	250.2636	377.4531
	26.54949	6.734988	27.06165	57.76013	118.366	174.1822	229.3018
	37.52518	38.7198	54.19632	118.3964	167.9238	268.9505	300.734
	34.09175	47.57731	74.9331	122.423	177.1629	286.5332	413.0334
	28.2437	16.4344	30.74125	80.75875	145.8583	219.3101	284.8488
	16.14225	34.56707	41.85544	98.49666	153.9187	232.8382	323.8748

Table of data for Fig. S1b

	ProSELENBP1			ProRBM39			ProRAD50			ProLACE1			Proc21orf63		
shLuc	87.35	84.01	128.64	89.42	96.34	114.24	74.65	101.88	123.46	94.20	109.45	96.35	102.36	101.16	96.48
shEXOSC9#1	110.50	187.77	153.28	141.28	129.03	130.30	184.96	169.77	248.20	135.84	172.20	145.33	101.76	117.41	70.11
shEXOSC9#2	167.13	167.37	147.41	130.29	133.96	136.68	243.68	178.68	181.63	152.35	148.77	151.78	91.24	89.98	102.46
shEXOSC2#1	741.42	492.18	508.51	246.42	324.65	333.94	705.62	723.05	863.37	246.96	316.97	244.10	140.51	109.11	139.31
shEXOSC4#1	511.30	406.30	382.76	215.89	183.22	222.03	287.30	321.42	381.34	218.09	213.19	218.47	129.80	118.71	109.75

Table of data for Fig. S2b

shLuc	7.3	6.5	7.8	8.2	7.2	7.15	8.7	7.8	6.75
shEXOSC9#1	7.9	8	6.9	8.4	7.4	8.1	8.1	6.8	8
shEXOSC9#2	8.65	8.05	7.6	8	6.95	8.05	8.15	7.95	6.95

Table of data for Fig. S2c

shLuc	7.3	6.15	6.85	7.25	7.5	6.75	7.9	7.8	7.85
shEXOSC9#1	5.6	4.25	4.75	5.45	5.45	5.7	5.25	5.7	5.75
shEXOSC9#2	5.1	4.6	5.4	5.1	5	5.75	4.4	5.75	5.2

Table of data for Fig. S2d

shLuc	6.4	5.9	5.85	5.75	6.2	6.7	6.1	6.5	5.8
shEXOSC9#1	4	5.2	4.4	4.5	4	4.95	4.6	4.55	3.95
shEXOSC9#2	4.35	3.85	4.7	3.8	4.2	5.2	4.75	5.1	4.7

Table of data for Fig. S2e

shLuc	5.85	5.5	5.65	4.6	5.8	5.2	5.5	5.35	5.95
shEXOSC9#1	3.9	3.15	3.7	3.7	3.85	4.65	3.75	3.6	3.55
shEXOSC9#2	3.75	3.55	4.65	4.3	3.65	3.55	3.45	4	3.5

Table of data for Fig. S2f

shLuc	6.05	6.85	6.1	6.7	6.4	6.45	5.75	6.6	6.35
shEXOSC9#1	4.45	4.35	4.3	4.95	4.65	5.15	5.1	4.2	4.2
shEXOSC9#2	4.8	4.6	4.8	4.9	4.8	3.95	4.65	4.8	3.9

Table of data for Fig. S2h

shLuc	6.9	6.35	7.1	6.35	7.3	7.75	6.95	6.7	8.25
shEXOSC9#1	6.4	6.1	8.15	6.5	7.5	6.6	7.6	7.2	7.4

shEXOSC9#2	6.55	7.5	8.1	6.4	7.2	8	7.5	6	7.3

Table of data for Fig. S2i

shLuc	6	6.7	7.05	6.2	7	7.95	6.05	5.2	8
shEXOSC9#1	4.15	4.2	5.05	4.45	4.8	4.55	4.35	3.65	5.3
shEXOSC9#2	3.9	3.7	4.3	4.25	4.15	5.95	4.8	3.6	5.25

Table of data for Fig. S2j

shLuc	4.3	3.9	4.9	4.15	4.85	4.95	4.55	5.05	4.7
shEXOSC9#1	2.4	3.1	2.75	2.85	3.25	3.25	3.15	3.15	2.9
shEXOSC9#2	2.55	3.15	3.55	2.4	2.95	2.8	2.85	3.2	2.8

Table of data for Fig. S2k

shLuc	4.6	4.5	4.95	4.95	4.65	5.6	5.55	4.8	6.85
shEXOSC9#1	3.1	3.3	4.3	3.15	3.35	3.75	3.75	3.2	4.15
shEXOSC9#2	3.4	2.85	4.15	2.8	3.15	4.15	2.75	2.6	3.4

Table of data for Fig. S2l

shLuc	5.15	4.65	5.4	4.75	4.75	5.3	4.35	4.65	6.05
shEXOSC9#1	3.1	2.95	3.2	2.75	3.45	3.85	2.6	2.8	3.9
shEXOSC9#2	2.75	2.75	3.15	3.1	2.65	3.4	2.9	2.75	3.55

Table of data for Fig. S3b

siLuc		siEDC4#1		siEDC4#2	
5	3	0	0	0	0
2	0	0	0	0	0
0	2	0	0	0	0
5	0	1	0	0	0
1	4	1	0	0	0
0	4	0	0	0	0
2	1	0	0	0	0
0	1	0	0	0	0
1	0	3	1	0	0
0	2	2	0	0	0
1	0	0	0	0	0
2	0	0	0	0	0
5	0	0	1	0	1
3	3	0	1	0	2
0	1	0	0	0	0
0	4	0	0	0	0
5	7	0	0	0	0
2	0	0	0	0	0
0	0	0	0	0	0
0	0	0	2	0	0
2	1	2	1	2	0
4	0	0	0	1	0
3	0	0	0	4	0
1	0	0	0	1	0
0	3	0	0	0	0
4	8	4	0	4	0
0	1	0	0	6	0
3	5	3	0	1	0
3	1	1	2	2	0
1	3	0	2	0	3
3	8	0	0	0	2
4	1	0	3	0	1
6	0	0	0	0	1
0	0	0	0	0	0
3	0	0	0	0	0
1	3	3	0	0	0
6	2	2	0	0	0
3	4	4	0	0	0
8	4	3	0	0	0
0	0	0	2	0	0
4	0	0	2	3	0
8	2	0	0	3	0
1	3	0	0	3	0
4	0	2	0	2	2
2	7	1	0	3	3
4	1	0	2	3	1
0	6	0	0	4	1
1	1	0	0	0	1
1	0	3	0	0	1

2	0	0	0	0	2

Table of data for Fig. S3c

siLuc		siEDC4#1		siEDC4#2	
0	2	0	0	0	3
0	2	1	0	0	0
1	0	0	0	0	0
1	3	0	0	0	2
0	2	0	2	3	0
2	0	0	0	0	2
0	2	0	1	0	3
0	3	0	0	1	0
7	1	0	0	0	0
1	3	0	0	2	0
0	2	3	1	0	0
4	4	0	0	0	0
0	3	3	0	1	0
6	4	0	0	0	0
4	9	0	0	1	0
0	0	1	0	2	2
4	2	0	1	0	0
2	2	0	0	0	3
2	5	0	0	0	0
3	0	3	0	0	0
2	2	0	2	1	2
0	0	1	1	0	3
0	4	0	0	1	0
5	0	2	0	0	0
2	0	3	1	2	0
15	5	0	0	3	3
2	0	0	0	0	0
4	0	2	1	0	0
0	3	1	0	2	0
0	0	0	2	3	0
2	0	0	0	0	1
0	0	1	0	0	2
1	3	0	0	1	4
1	1	0	0	0	0
1	4	0	0	3	0
0	1	1	0	2	0
1	4	0	0	0	0
0	5	0	0	0	3
2	0	0	2	0	0
2	1	0	1	1	0
1	3	0	0	0	0
0	1	0	0	0	0
0	4	0	0	2	0
4	0	2	0	0	0
3	4	2	0	3	0
2	4	4	0	0	0
3	1	1	0	0	0
7	0	0	0	0	4
3	4	0	2	0	0
4	2	0	1	0	0

Table of data for Fig. S3d

siLuc		siEDC4#1		siEDC4#2	
0	0	1	0	0	0
2	0	3	0	0	0
0	0	3	0	0	0
3	0	1	2	0	0
0	2	3	0	0	1
3	1	1	0	0	0
3	2	0	0	0	0
0	0	0	0	0	0
1	3	0	0	0	3
1	0	0	0	0	0
0	0	0	0	0	1
0	0	0	0	0	0
0	0	0	3	0	3
2	1	0	0	0	0

2	3	0	0	1	0
0	0	1	0	0	0
0	0	0	0	0	3
4	2	0	3	0	4
0	1	0	0	0	0
2	0	0	2	0	0
0	2	0	0	0	0
0	1	0	0	4	0
0	3	0	0	0	0
4	2	1	0	0	0
0	1	0	0	2	0
0	0	0	0	0	0
1	1	0	0	0	0
7	0	0	0	0	2
2	4	0	0	0	1
3	1	0	0	0	0
2	2	0	0	0	0
0	0	0	0	3	0
0	2	2	0	1	0
0	3	0	0	0	2
1	0	0	0	0	0
0	0	1	0	0	0
0	0	0	0	0	0
0	2	0	1	3	0
1	0	3	0	3	0
3	1	0	0	1	0
2	3	0	0	0	0
3	2	0	0	0	0
5	3	1	0	0	0
0	0	0	0	3	0
0	0	0	0	2	0
0	0	0	0	1	0
0	1	0	2	0	0
0	1	2	0	0	2
0	0	0	0	0	0
0	1	0	0	0	0

Table of data for Fig. S3e

siLuc	6.45	7.7	7.4	8.55	8.05	7.65	7.2	7	7.95
siEDC4#1	6.45	8.4	7.8	7.95	7	8.55	6.75	7.35	7.25
siEDC4#2	7.65	7.7	8	7.05	7.7	7.6	6.6	8.05	7.75

Table of data for Fig. S3f

siLuc	7.35	7.35	7.6	6	7.35	7.2	7.05	7.7	7.6
siEDC4#1	4.5	4.2	3.6	3.6	3.85	3.65	3.75	3.15	4.25
siEDC4#2	4.2	3.85	4.05	4.05	4.9	4.1	3.45	3.85	4.65

Table of data for Fig. S3g

siLuc	7.65	7.7	8.1	6.75	7	7.55	6	7.7	7.45
siEDC4#1	4.95	4.9	4.15	5.55	4.2	3.95	3.75	4.9	4.6
siEDC4#2	5.4	4.55	3.95	3.85	4.2	3.55	4.8	3.5	4.4

Table of data for Fig. S3h

siLuc	4.95	4.2	5.15	6.55	4.55	4.1	4.95	3.85	4.45
siEDC4#1	3.75	1.75	2.1	4.7	2.1	1.95	3.3	1.75	1.65
siEDC4#2	3.75	1.4	1.55	3.45	2.45	1.15	2.85	2.1	2.4

Table of data for Fig. S3i

siLuc	4.95	5.25	5.45	7.05	5.4	6.05	4.95	4.9	5.6
siEDC4#1	3.75	2.45	1.9	5.1	3.15	2.8	3.3	2.8	2.45
siEDC4#2	3.75	3.15	2.75	3.45	2.8	2.35	2.85	1.75	2.15

Table of data for Fig. S4a

shLuc		shEXOSC9#1		shEXOSC9#2	
0	9	0	3	0	0
15	8	0	4	0	0
0	0	0	0	1	3
4	5	4	0	2	9
6	5	5	12	2	5
12	8	0	5	0	0
11	8	3	8	0	0
4	8	0	0	0	0

3	2	0	0	0	3
0	0	4	2	0	3
13	8	3	5	3	4
6	4	0	7	3	3
10	6	3	11	2	0
0	0	3	1	0	5
5	6	6	3	6	0
7	14	6	0	0	0
8	0	4	3	0	1
12	13	9	0	0	14
0	10	5	0	5	0
0	10	2	0	5	0
5	9	2	0	1	0
7	6	0	11	0	0
5	7	0	3	11	3
10	12	2	0	0	1
0	7	0	1	9	0
9	3	3	1	1	6
5	7	0	0	0	0
6	0	1	5	7	4
7	12	6	0	0	0
5	2	8	0	0	0
5	0	0	2	4	0
12	6	1	0	5	1
7	7	2	1	2	2
0	21	0	3	2	3
6	3	6	1	4	2
4	5	0	0	0	0
7	0	0	1	0	0
0	6	1	0	2	5
0	16	8	2	5	4
12	7	4	2	5	0
5	3	3	2	0	0
10	4	0	5	0	0
17	6	4	2	1	2
14	0	3	6	3	0
8	5	2	5	0	5
0	11	12	0	6	0
12	0	0	4	2	2
7	1	2	3	3	4
14	2	0	3	3	4
6	15	0	2	2	2

Table of data for Fig. S4b

shLuc		shEXOSC9#1		shEXOSC9#2	
10	0	0	4	0	2
4	8	6	0	0	5
0	5	6	2	0	1
8	0	2	5	0	0
0	2	5	0	9	0
0	0	4	5	0	0
2	13	3	2	0	0
7	9	0	0	0	12
0	10	0	0	7	2
13	0	5	3	0	0
5	0	2	7	0	3
0	5	6	2	3	0
3	6	0	0	2	4
8	12	0	0	0	3
0	13	0	10	0	0
7	12	5	1	0	0
7	5	0	1	0	5
0	0	0	0	0	0
0	0	0	0	0	5
4	4	0	0	0	8
0	5	8	0	0	0
0	10	0	0	0	0
5	3	0	0	5	0
8	0	8	3	0	0
6	0	1	0	0	2
4	11	0	5	0	4
4	0	0	1	0	1
5	12	2	1	0	0

0	3	4	2	0	7
2	5	0	0	0	0
3	0	0	6	0	2
2	5	0	3	6	0
6	6	0	0	6	0
3	6	0	0	0	0
0	4	2	2	4	0
2	5	10	7	1	2
5	0	3	0	2	0
6	0	2	0	0	5
9	0	0	0	3	0
4	13	5	0	0	2
0	6	0	0	3	4
4	9	3	0	2	0
2	6	0	6	0	0
3	7	0	0	2	1
7	0	0	0	6	0
7	10	0	1	1	3
0	0	3	0	0	7
7	2	7	0	0	0
8	7	3	0	0	5
5	4	2	0	5	0

Table of data for Fig. S4c

shLuc		shEXOSC9#1		shEXOSC9#2	
0	0	1	0	0	5
3	0	0	3	6	3
5	0	0	0	0	0
10	0	0	0	1	0
5	3	0	0	2	1
1	7	1	5	1	3
0	4	0	1	7	1
1	5	1	0	1	0
0	0	3	0	0	0
3	0	2	1	0	0
0	8	0	5	1	0
2	2	3	1	8	0
5	0	1	3	0	0
5	6	2	0	2	3
1	5	0	0	0	4
0	2	1	0	4	0
0	1	3	4	3	0
2	3	0	0	0	3
5	4	3	1	0	2
6	5	0	0	2	0
6	0	1	0	0	0
0	3	0	0	0	0
0	8	0	1	1	1
0	4	0	2	0	7
2	6	1	3	0	0
4	2	0	0	6	0
4	3	1	2	3	5
4	4	5	2	5	4
2	0	1	0	1	1
0	5	1	0	0	0
0	6	0	0	0	0
2	8	0	5	0	2
2	4	2	2	0	3
0	6	0	3	0	0
5	0	0	2	5	7
5	0	0	1	2	0
8	2	0	0	6	8
2	11	0	3	4	1
0	0	1	0	1	0
0	0	0	0	0	1
4	5	6	0	0	1
6	0	0	0	3	0
2	1	0	0	4	0
1	3	0	0	1	0
0	9	0	0	0	0
6	5	6	3	4	1
9	0	4	5	1	0
0	0	1	0	4	4

0	6	3	2	5	0
3	0	6	2	1	1

Table of data for Fig. S4d

shLuc		shEXOSC9#1		shEXOSC9#2	
5	2	5	0	4	3
0	0	5	0	2	0
5	0	0	2	0	0
11	7	0	1	0	0
5	8	3	4	0	0
1	0	0	3	3	0
0	3	10	3	4	0
3	9	5	0	5	0
0	5	0	1	0	0
6	7	0	2	2	0
0	2	0	0	0	0
0	3	3	0	0	0
2	4	0	12	0	0
4	1	0	6	1	3
3	2	0	0	0	0
0	6	1	5	0	0
5	3	3	0	0	7
0	6	0	4	2	0
6	2	0	0	0	0
8	2	1	3	0	0
2	0	0	0	0	3
0	0	1	0	0	0
5	3	0	0	0	0
0	0	0	2	0	0
4	3	0	0	0	0
0	5	0	4	0	0
5	5	0	0	0	0
5	3	4	5	0	0
2	0	0	3	7	0
8	0	0	0	0	7
3	0	0	0	8	0
4	4	0	3	0	0
5	3	2	1	4	0
5	0	5	0	0	0
2	0	0	10	0	0
0	3	3	0	6	0
4	0	0	0	0	4
4	0	0	6	3	0
6	0	0	1	0	0
0	1	0	0	0	0
0	0	2	4	2	1
7	6	0	0	0	6
3	4	0	0	0	0
0	0	8	0	0	0
3	4	0	5	0	0
0	3	0	3	5	0
0	2	0	2	0	0
2	0	0	0	4	4
2	4	2	6	3	0
0	2	0	0	0	0

Table of data for Fig. S5a

shLuc		shEXOSC9#1		shEXOSC9#2	
11	13	6	0	0	2
13	24	4	0	8	10
10	12	0	12	4	0
12	9	3	4	0	0
14	10	5	3	1	9
29	14	8	8	7	0
14	9	0	7	0	1
16	12	0	0	4	3
6	5	0	8	0	1
4	0	7	0	5	0
3	3	2	4	5	8
3	0	0	1	9	10
9	12	2	5	0	0
9	12	0	6	0	4
19	16	0	0	0	5

16	11	7	8	1	0
17	3	2	0	10	4
23	0	0	9	2	3
5	16	1	2	6	4
2	13	4	0	0	12
16	3	24	4	0	0
8	10	0	5	0	4
15	12	6	7	3	0
0	12	2	3	0	0
19	33	0	4	9	0
17	24	0	3	5	0
17	3	4	3	1	2
11	18	0	0	0	1
15	8	0	0	7	4
6	2	1	12	0	0
6	11	0	0	13	2
3	10	3	0	3	0
15	2	5	9	10	5
8	19	3	0	0	0
5	6	4	3	3	5
4	8	4	0	3	0
0	8	0	3	7	2
12	3	2	3	0	6
14	10	4	1	6	0
12	4	0	0	6	0
7	8	2	2	4	0
4	8	4	2	4	6
9	14	4	3	5	5
5	5	7	0	8	5
8	15	0	0	0	0
8	6	0	8	0	2
7	22	0	0	1	0
14	15	0	0	0	11
16	17	0	0	0	3
4	7	0	3	4	3

Table of data for Fig. S5b

shLuc		shEXOSC9#1		shEXOSC9#2	
9	14	7	0	0	6
0	8	6	2	4	0
8	6	0	1	3	4
8	11	5	1	0	3
8	9	7	3	3	2
4	10	7	3	0	5
7	10	4	0	0	5
10	20	1	0	5	4
15	12	5	4	0	4
7	8	4	0	9	3
0	15	2	8	3	2
0	8	0	9	4	0
36	0	4	14	6	8
10	5	0	0	0	4
8	10	3	0	5	0
8	8	0	2	0	4
5	7	2	0	7	0
21	7	1	0	0	0
25	9	0	2	0	0
17	3	6	0	0	0
4	6	7	2	3	3
8	16	0	3	4	0
0	7	0	0	6	1
10	2	17	0	0	0
11	9	0	2	4	3
10	18	0	3	1	0
12	6	4	8	9	4
7	5	0	6	1	4
14	6	7	3	4	5
16	6	0	0	0	6
0	5	3	1	1	0
9	9	0	2	0	0
8	4	0	0	5	0
6	10	0	0	4	0
12	8	0	2	5	6

11	8	0	0	4	1
8	10	0	0	4	3
6	7	0	0	5	0
11	11	4	0	12	0
0	14	0	6	6	0
16	13	0	6	0	0
5	19	3	1	0	0
6	0	1	3	0	7
7	8	0	0	3	3
8	6	4	2	5	4
9	0	0	0	3	0
14	6	4	0	4	0
12	0	1	0	6	0
9	0	6	3	8	4
3	4	5	0	0	5

Table of data for Fig. S5c

shLuc		shEXOSC9#1		shEXOSC9#2	
5	2	0	0	1	2
0	2	0	0	3	2
4	6	0	3	0	0
3	8	0	3	2	0
1	2	0	3	4	0
8	6	0	2	5	0
2	4	0	3	1	3
0	6	4	1	5	0
12	6	0	0	6	2
7	2	1	0	0	0
0	6	0	2	0	5
11	1	1	0	2	0
2	1	0	0	0	0
6	0	2	1	1	1
7	3	0	2	0	0
7	7	0	3	0	4
4	9	1	0	1	0
2	4	0	0	5	0
6	0	0	0	0	0
10	0	0	0	1	0
0	4	0	0	0	0
0	2	1	0	0	2
5	5	0	0	0	4
4	0	0	0	7	5
2	3	1	0	1	0
2	4	0	0	0	0
3	7	0	1	2	0
3	4	0	0	1	0
0	3	1	1	5	4
3	2	0	0	0	0
3	5	3	0	0	1
0	8	0	8	0	0
6	0	2	1	6	0
10	0	0	1	0	2
3	7	0	0	0	0
4	0	0	0	0	0
0	6	0	0	3	2
3	0	0	0	1	3
2	1	0	0	0	0
4	7	0	1	0	0
3	3	1	0	4	3
3	4	0	0	2	0
7	4	1	0	0	0
12	0	1	0	0	0
16	5	0	1	0	0
9	0	0	3	0	0
5	8	3	1	5	0
3	6	1	1	0	2
1	2	0	1	0	2
4	1	0	0	1	3

Table of data for Fig. S5d

shLuc		shEXOSC9#1		shEXOSC9#2	
6	0	5	1	0	7
4	4	0	0	0	0

2	0	0	4	0	3
7	4	0	0	0	0
1	1	0	3	0	1
5	0	0	4	1	0
2	5	0	0	0	0
0	0	1	1	8	0
5	2	0	0	1	3
7	4	0	4	3	0
1	0	0	3	0	0
3	3	3	0	0	2
2	0	0	1	2	0
7	5	0	0	4	4
0	7	0	6	3	0
2	8	0	0	0	1
3	0	0	3	0	0
13	1	0	0	0	1
2	4	0	3	4	0
8	6	0	1	2	2
5	3	2	0	0	0
1	8	2	0	0	0
0	7	0	3	0	0
5	5	0	0	3	0
10	0	0	0	0	5
0	5	0	0	0	0
3	7	0	4	0	4
6	2	2	0	0	16
3	7	0	2	0	0
3	0	3	0	0	3
0	5	0	0	1	4
3	0	0	0	3	4
0	8	0	1	0	0
5	4	13	0	0	0
5	0	0	0	1	8
3	2	0	0	0	2
2	4	0	5	0	0
4	3	1	4	0	4
1	0	0	0	0	0
3	0	0	0	0	0
5	4	0	0	8	0
4	4	0	0	0	0
4	3	3	0	0	0
0	0	0	1	3	0
0	4	0	0	0	3
1	3	4	0	0	3
0	4	4	3	0	0
0	0	0	4	13	0
1	0	0	1	4	2
5	4	0	0	0	0

Table of data for Fig. S1b

	ProSELENBP1			ProRBM39			ProRAD50			ProLACE1			Proc21orf63		
mock	93.91	105.94	100.15	94.32	115.73	89.95	111.51	87.60	100.89	107.32	93.45	99.23	101.14	110.33	88.53
WT	53.78	67.62	37.18	72.63	59.99	67.43	58.14	47.58	57.26	67.03	52.92	54.17	114.10	103.21	102.32
MUT	119.76	75.18	79.78	98.79	100.02	100.42	94.33	89.80	105.01	116.04	113.40	119.86	115.88	114.69	106.34

Table of data for Fig. S1c

	DAPK1			PYCARD			TNFRSF1B			TNFRSF21		
mock	106.90	98.76	94.34	92.26	102.78	104.96	105.72	83.84	110.45	94.52	98.30	107.19
WT	73.03	68.57	73.96	57.94	58.42	51.41	28.20	38.71	26.60	42.78	37.34	37.10
MUT	72.96	126.39	128.90	92.83	99.94	88.37	97.42	104.54	125.85	120.28	109.06	107.26

Table of data for Fig. S6f

shLuc		shAPOBEC3G#1		shAPOBEC3G#2	
0	3	4	1	5	3
0	0	0	0	0	4
2	3	2	1	0	0
0	1	0	4	0	8
0	5	1	3	0	4
0	0	2	3	1	2
0	1	9	1	7	0
0	0	0	2	0	8
0	3	0	2	2	0
0	4	7	3	5	3

4	0	5	2	0	0
0	1	1	0	4	0
3	0	0	6	0	2
0	0	0	0	0	2
1	4	1	2	2	4
0	0	3	9	2	1
7	0	3	5	2	0
0	0	0	8	4	0
2	5	1	1	3	6
0	2	3	0	1	4
4	1	2	3	0	0
0	0	0	7	8	4
0	0	0	6	4	3
0	0	4	1	2	5
0	0	0	0	0	0
0	1	2	0	5	0
0	0	0	9	0	0
0	0	9	4	4	0
1	0	3	2	2	0
0	0	4	0	1	4
0	0	4	0	4	0
2	0	1	3	8	6
8	0	1	4	0	7
0	0	1	5	0	1
5	0	0	2	7	0
4	0	4	5	0	0
0	0	1	0	1	9
0	0	0	0	5	7
0	0	4	4	2	0
1	1	4	4	5	0
0	0	0	3	1	7
0	0	0	0	0	15
0	0	3	2	4	0
2	0	2	0	7	0
0	0	6	1	5	6
0	0	2	3	0	3
3	3	0	4	2	5
2	0	7	2	0	6
0	0	3	4	0	0
0	0	3	2	5	4

Table of data for Fig. S6g

shLuc		shAPOBEC3G#1		shAPOBEC3G#2	
2	1	0	0	1	3
1	0	4	1	0	5
0	0	4	0	1	9
2	0	0	3	6	1
0	0	1	1	2	4
3	0	0	1	1	0
1	4	6	3	2	2
0	3	3	0	1	0
0	1	0	1	1	3
0	1	0	4	4	3
4	1	2	2	0	4
5	0	0	0	3	0
0	0	2	1	0	2
4	3	0	2	1	4
0	0	0	1	0	0
3	3	3	0	3	1
0	2	1	3	2	3
0	0	3	0	0	0
0	0	3	6	0	2
3	1	0	3	4	3
0	0	3	3	0	2
0	0	4	1	2	5
3	0	1	0	1	3
0	0	1	5	6	3
0	0	8	1	4	3
0	0	0	0	0	1
3	0	3	2	0	3
1	2	0	0	0	3
2	4	3	0	2	5
0	0	3	3	0	1

2	0	0	3	4	0
6	0	3	1	3	0
2	1	1	1	0	3
0	0	1	3	3	0
4	2	0	1	1	3
1	0	3	0	0	0
0	4	0	2	0	1
3	4	1	4	0	3
0	0	3	3	1	4
0	0	0	2	4	3
3	1	2	6	3	6
0	1	2	2	2	0
1	1	3	1	2	0
5	1	0	2	2	1
2	2	0	0	3	4
0	2	4	1	3	2
0	0	2	0	0	2
3	2	0	3	4	0
0	0	5	2	1	3
4	2	0	3	0	2

Table of data for Fig. S7b

mock	6.6	7.65	8	7.45	7.55	8.2	7.95	8.95	8.75
APOBEC3G-V5	7.35	9	8.1	7.9	8.05	7.9	7.05	8.2	9

Table of data for Fig. S7c

mock	8	8.4	8.9	8	8.3	9.15	8.5	8.3	9
APOBEC3G-V5	6.55	6.8	6.4	6.75	7.9	6.65	6.6	7.25	6.8

Table of data for Fig. S7d

mock	7.45	8.5	8.4	7.85	9.2	9.7	7.65	8.8	7.5
APOBEC3G-V5	6.5	6.4	6.5	6.55	6.55	6.55	6.2	6.5	5.5

Table of data for Fig. S7e

mock	6.35	6.65	9	5.5	6.8	7.4	5.6	6.85	7.1
APOBEC3G-V5	5.3	5.75	6.6	5.35	5.35	6.2	5.2	5.45	4.9

Table of data for Fig. S7f

mock	7.15	7.95	5.4	6.65	8.4	4.3	6.9	7.95	5.5
APOBEC3G-V5	5.55	6.15	3.4	5.7	6.6	3.4	5.75	5.7	3.1

Table of data for Fig. S7g

mock		APOBEC3G-V5	
8	0	0	5
0	5	4	4
5	8	13	6
0	5	2	2
4	5	4	0
6	0	6	0
7	25	7	3
5	6	7	3
0	0	3	4
4	6	4	5
2	4	3	2
5	0	0	4
6	5	0	3
6	3	1	0
5	5	2	3
4	7	0	3
10	8	3	8
3	6	8	3
8	8	2	4
4	6	0	0
4	3	0	0
12	8	9	4
0	5	0	2
6	3	5	2
1	14	6	5
8	4	8	4
5	7	0	3
5	6	4	0
4	3	3	9
9	6	6	4

1	3	2	0
4	8	4	2
18	4	3	4
6	10	6	0
12	0	2	9
25	4	7	9
2	14	3	3
3	6	0	3
5	5	6	4
4	7	10	3
4	6	2	4
5	3	2	5
3	8	1	4
2	2	0	3
4	1	0	5
3	3	0	2
4	4	6	5
7	2	5	4
11	4	4	10
3	1	2	0

Table of data for Fig. S7h

mock		APOBEC3G-V5	
6	3	8	4
5	10	4	2
6	4	4	6
11	5	8	6
7	3	8	0
7	4	4	0
5	4	2	10
4	1	1	3
10	8	5	0
4	4	0	4
4	3	5	5
6	3	3	8
1	14	6	4
7	9	6	8
4	7	9	3
6	4	8	3
4	11	6	4
7	4	3	1
5	4	7	5
5	6	0	18
5	4	10	7
3	7	0	0
3	7	0	0
6	4	3	0
7	7	0	4
3	6	4	5
6	4	9	8
4	8	5	6
5	6	7	7
6	5	6	7
3	6	6	5
4	1	0	0
10	5	11	5
4	4	4	5
4	4	3	3
5	9	4	2
6	7	4	5
4	5	5	4
6	7	6	7
3	5	0	6
3	2	6	1
7	6	1	0
2	5	4	0
3	6	0	0
6	6	10	0
5	6	5	7
5	13	0	6
5	6	9	3
5	3	3	0
4	3	10	7

Table of data for Fig. S7i

mock		APOBEC3G-V5	
0	1	2	0
9	3	3	2
7	3	0	3
1	0	0	5
1	1	0	2
5	1	4	1
3	8	3	2
1	1	2	0
4	5	0	2
4	4	4	2
3	6	4	1
19	3	1	0
9	4	5	1
0	7	4	6
5	6	3	2
4	2	5	4
3	5	2	0
1	3	1	2
3	3	0	2
7	1	1	2
4	0	2	1
4	5	5	3
2	2	1	6
3	1	3	1
3	3	0	4
3	3	3	0
1	0	4	0
5	0	3	3
1	9	0	4
1	0	3	2
1	0	0	6
2	6	1	6
1	2	4	4
4	3	2	3
1	0	2	3
5	12	0	0
3	0	1	1
1	1	1	3
2	6	4	7
1	4	5	1
5	3	4	3
5	3	3	2
2	4	3	2
6	4	2	2
3	4	3	0
0	0	2	0
4	0	5	0
2	5	2	0
2	1	0	5
4	4	1	6

Table of data for Fig. S7j

mock		APOBEC3G-V5	
5	4	5	0
0	1	1	0
1	2	6	3
1	2	1	7
2	0	0	1
4	0	5	2
3	2	0	3
0	3	2	3
8	1	5	2
3	1	1	6
9	3	4	2
3	4	0	4
2	1	2	3
0	0	4	0
3	1	4	1
2	15	4	1
3	0	0	3

0	1	0	3
4	0	3	3
6	3	7	4
5	4	1	3
2	1	3	3
2	3	2	4
4	2	2	0
5	2	6	3
4	2	3	3
4	3	1	2
2	2	3	0
12	0	0	5
1	3	4	0
1	2	0	0
2	3	0	1
4	1	0	1
2	0	4	0
0	15	0	4
2	4	2	5
2	3	0	0
1	1	4	3
1	4	3	0
0	2	5	0
2	4	1	3
6	4	4	8
2	1	1	4
1	1	5	9
2	6	5	5
1	2	0	0
5	3	5	0
5	0	0	1
4	1	0	3
4	2	2	3

Supplementary Table S6. Original data of graphs shown in figures
